# Synthesis and Characterization of a Variety of α,ω-Bisacylpolysilanes—A Study on Reactivity and Accessibility

**DOI:** 10.1021/acsomega.2c05258

**Published:** 2022-10-13

**Authors:** Tanja Wiesner, Madeleine Heurix, Roland C. Fischer, Ana Torvisco, Michael Haas

**Affiliations:** Institute of Inorganic Chemistry, Graz University of Technology, Stremayrgasse 9/IV, 8010 Graz, Austria

## Abstract

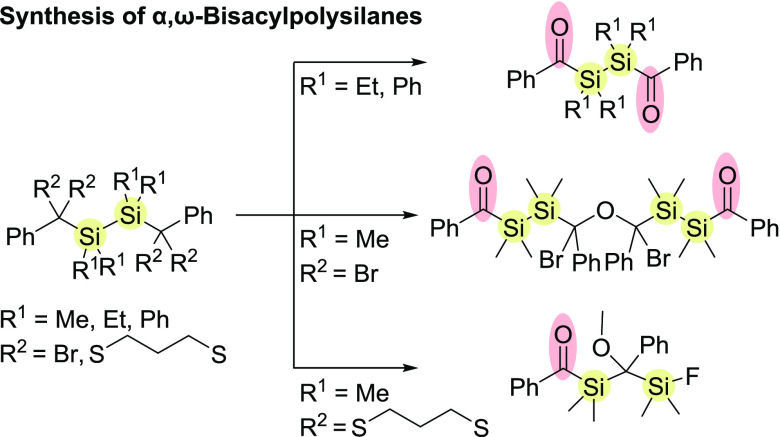

In this study, a variety of α,ω-bisacylpolysilanes were synthesized via two synthetic protocols. The first method for obtaining these compounds is based on the substitution reaction of bromine either on silica gel or by the use of silver salts. Surprisingly, instead of the expected bromine substitution product PhC(O)(SiMe_2_)_2_C(O)Ph **4a**, we found the formation of the diastereomer PhC(O)(SiMe_2_)_2_CBrPhOCBrPh(SiMe_2_)_2_C(O)Ph **4b** indicating a more complex reaction cascade. On the other hand, the phenylated compound **3b** yielded the expected bromine substitution product PhC(O)(SiPh_2_)_2_C(O)Ph **4c**. For the second protocol, we utilized the Corey–Seebach approach to isolate other representatives of this compound class. We found that the substituents at the α-silicon atoms influence the selectivity of the dethioketalization. While the ethylated and phenylated disilanes **5b**,**c** yield the expected bisacyldisilanes **6a**,**b**, the methylated disilane **4a** undergoes a BF_3_-induced Si–Si bond breakage followed by an intermolecular sila-aldol reaction. This hitherto unknown sila-aldol reaction results in the formation of the enantiomer PhC(O)SiMe_2_C(OMe)PhSiMe_2_F **6c** in excellent yields. All isolated compounds were analyzed by a combination of NMR spectroscopy, ultraviolet–visible (UV–vis) spectroscopy, single-crystal X-ray crystallography, and mass spectrometry. Furthermore, the photochemical pathways of two representative examples (**4b**,**c**) were examined.

## Introduction

**Scheme 1:**
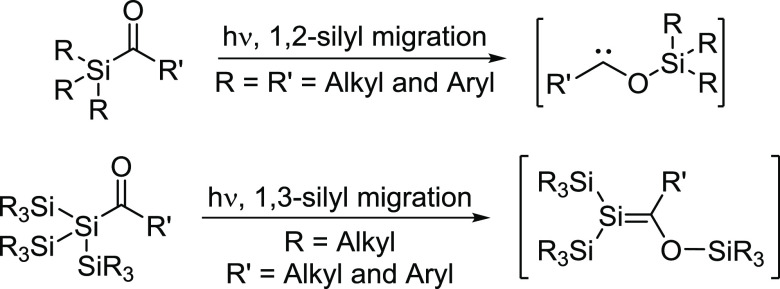
Photochemistry of Acylsilanes and Branched Acylpolysilanes

The photochemical rearrangement of simple acylsilanes, such as R_3_Si(CO)R′, mainly follows a Norrish type II mechanism. Moreover, this 1,2-silyl migration yielding a siloxycarbene is manifested in the literature and is published with several examples.^1−8^ The same holds true for branched acylpolysilanes with the general formula (R_3_Si)_3_Si(CO)R. These molecules undergo a 1,3-silyl migration and form stable or metastable silenes, depending on the residue on the carbonyl function (see Scheme 1).^9−12^

**Scheme 2:**
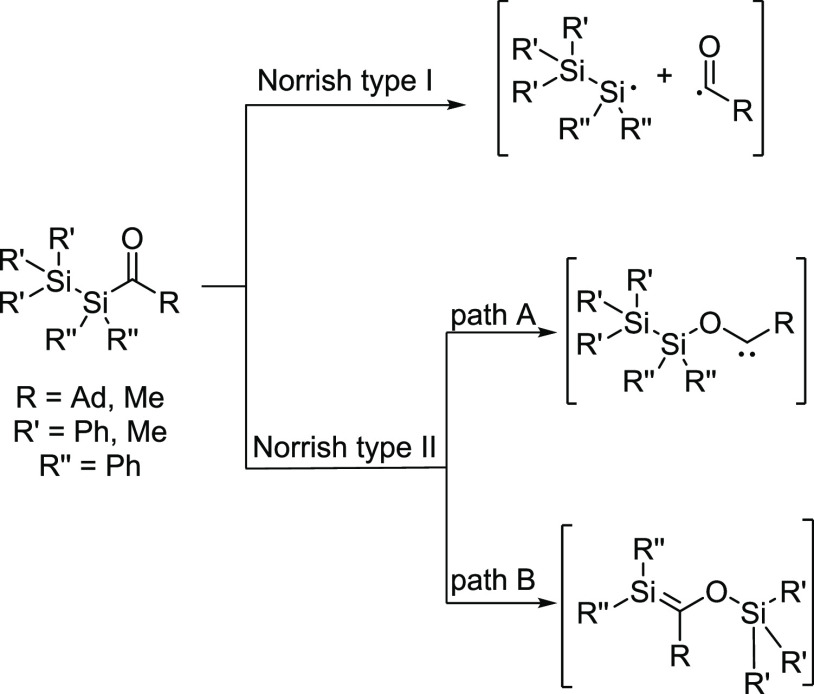
Photochemistry of Linear Acylpolysilanes

In contrast, only two publications on linear acylpolysilanes, which evaluated their photochemical pathways in greater detail, can be found in the literature.^13,14^ As outlined by Brook et al., their photochemical pathway is more complex as both Norrish-type pathways (I and II) are detectable (Scheme 2). Moreover, there are two silyl rearrangements (1,2- and 1,3-silyl migration) following Norrish type II present.

The possibility that these derivatives can undergo Norrish type I rearrangements piqued our interest. On this basis, an application as a silicon-based photoinitiator (PI) is theoretically possible. In general, silicon as the central atom would provide a harmless PI because silicon shows no element-specific toxicity. Additionally, due to its high abundance in the Earth’s crust, it can compete with the widely used but toxic phosphorus-based PIs.^15,16^

Herein, we have merged two acyl groups with a polysilane skeleton in one molecule to take advantage of the respective material properties of polysilanes as well as acylsilanes as described in the literature.^17,18^ Therefore, we set out and developed new pathways toward the promising compound class of bisacylpolysilanes as potential photoinitiators.

## Results and Discussion

To synthesize the target molecules, we used two pathways. The first approach is based on a bromine substitution (pathway I), and the second approach is the Corey–Seebach reaction (pathway II).

### Pathway I

**Scheme 3:**
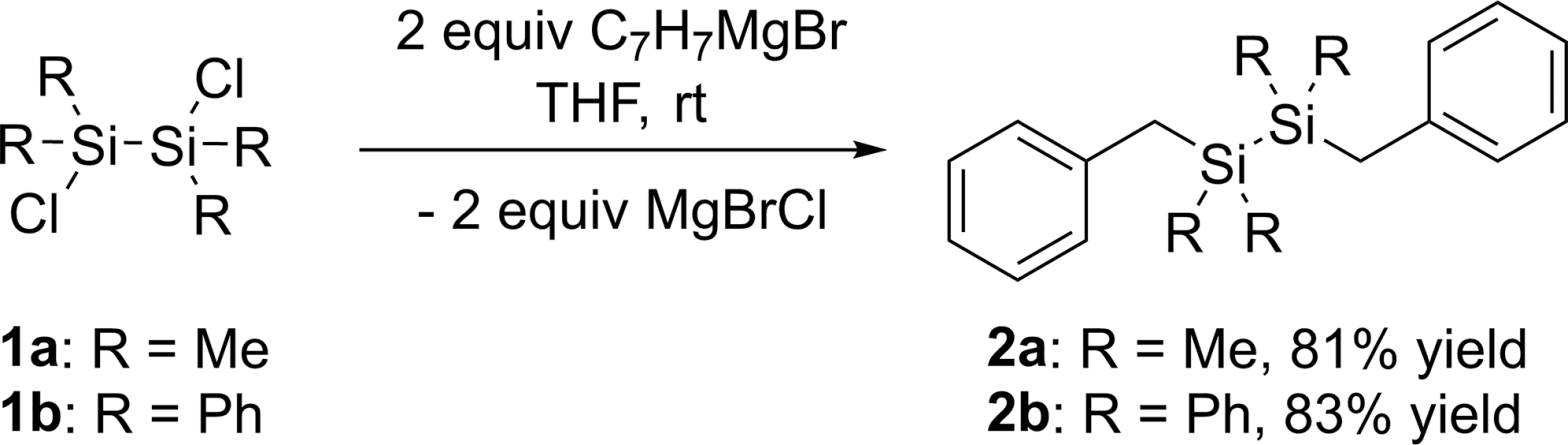
Synthesis of Dibenzyldisilanes **2a**,**b**

The starting point of our investigations is provided by the straightforward synthesis of two dibenzyldisilanes in excellent yields. Therefore, the corresponding dichlorodisilanes **1a**,**b** are reacted with a 2-fold excess of benzylmagnesium bromide in tetrahydrofuran (THF) (Scheme 3).

Analytical data are consistent with the proposed structure. Detailed characterizations for **2a**,**b** are provided in the Experimental Section. It should be noted that **2a** is an already literature-known compound; however, our approach results in the formation of **2a** with the highest yield.^19,20^ Both derivatives show nearly identical ^13^C NMR chemical shifts for the benzylic carbon atom at δ = 25.10 ppm (for **2a**) and at δ = 23.78 ppm (for **2b**), which is characteristic for benzylic carbon atoms directly substituted to silicon atoms. The ^29^Si NMR spectra of **2a**,**b** showed one resonance for the two magnetically equivalent silicon atoms at δ = −17.08 ppm (for **2a**) and δ = −19.86 ppm (for **2b**).

### Bromination

**Scheme 4:**
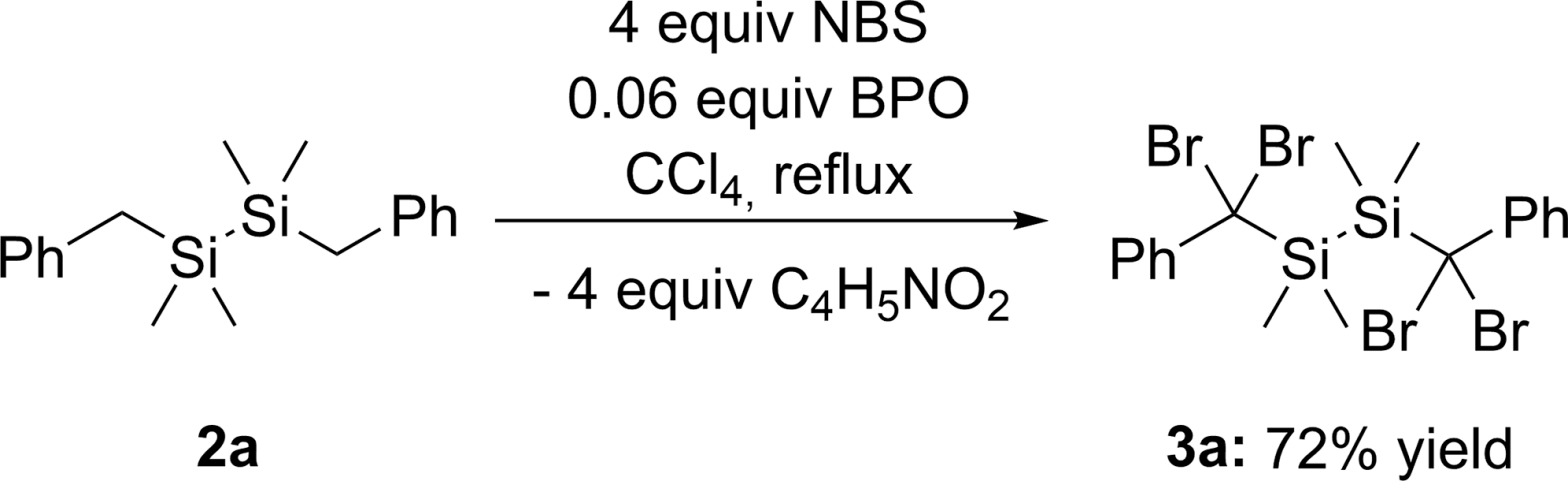
Bromination of **2a**

The bromination of compounds **2a**,**b** was adapted from literature for monosilanes.^21^ The reaction was performed in CCl_4_ solution with four equivalents of *N*-bromosuccinimide (NBS) and catalytical amounts of benzoyl peroxide (BPO) as the radical initiator. The tetrabromination of **2a** was complete within 16 h (monitored by NMR spectroscopy). The air-stable and crystalline compound **3a** was obtained with an isolated yield of 72% (compare Scheme 4). Spectroscopic and analytical data, which support the structural assignment, are summarized in the Experimental Section with experimental details. The molecular structure of **3a** was determined by single-crystal X-ray crystallography and is incorporated in the Supporting Information (compare Figure S41 and Table S1). Compound **3a** crystallized in the monoclinic space group *P*2_1_ with unexceptional bond lengths and angles. The unit cells comprise two molecules (see the Supporting Information).

**Scheme 5:**
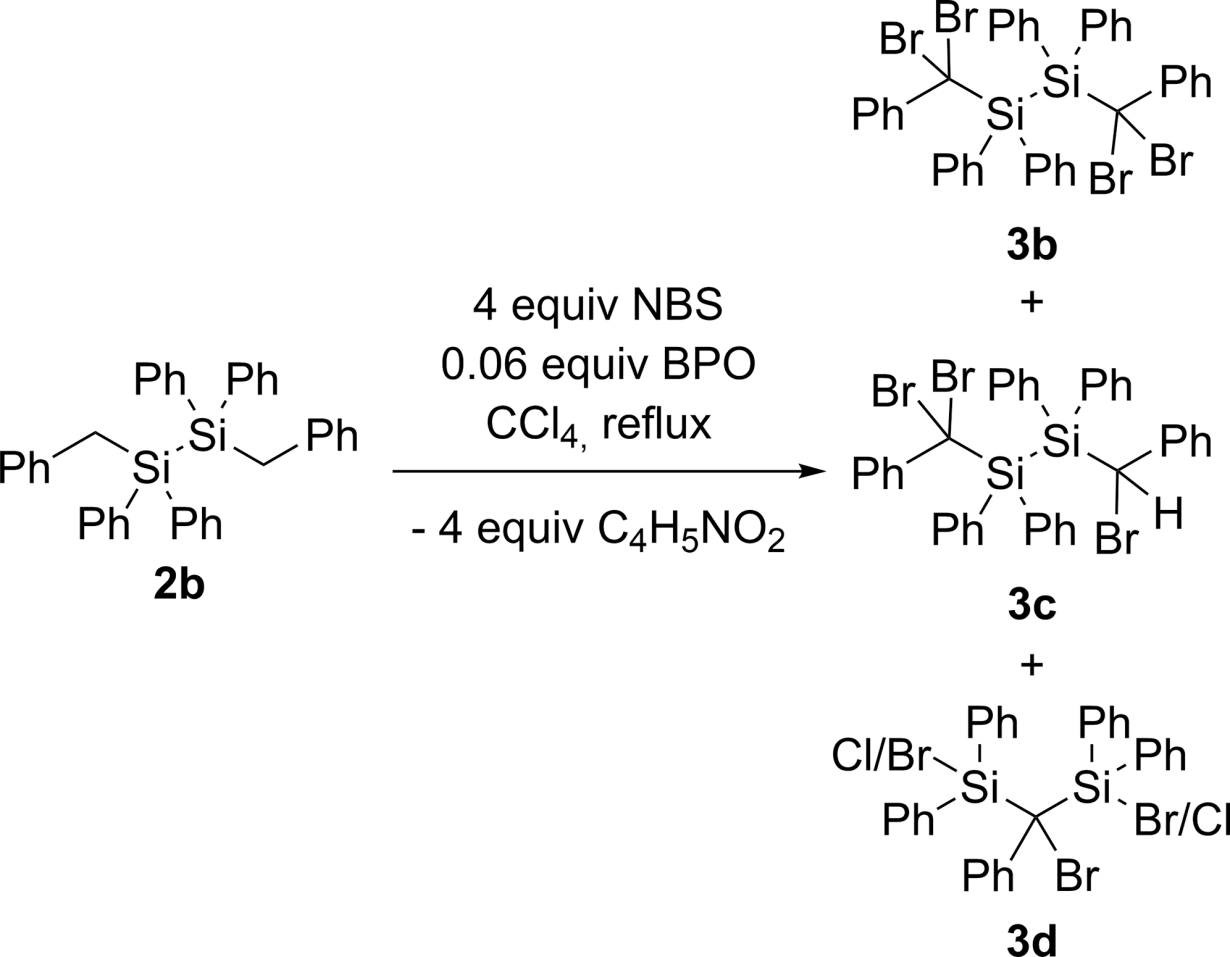
Bromination of **2b**

In contrast to the good selectivity of the bromination of **2a**, we observed that the selectivity of the bromination of **2b** was significantly lower. No complete conversion to the tetrabrominated species was found after refluxing in CCl_4_ for 16 h. This is also in agreement with the literature, which reports on nonselective bromination when phenyl groups are bonded to the silicon atom.^21^ After several attempts to optimize the synthesis and monitor the reaction progress via NMR spectroscopy, we found, besides the formation of the target compound, two additional side products (see Scheme 5). Concerning the partially brominated compound **3c**, a peak at δ = 5.37 ppm in the ^1^H NMR spectra can be assigned to the corresponding proton of the H–C–Br fragment (Figure S10). The other side product **3d** indicates extended scission of the Si–Si bond upon prolonged reaction time.

**Figure 1:**
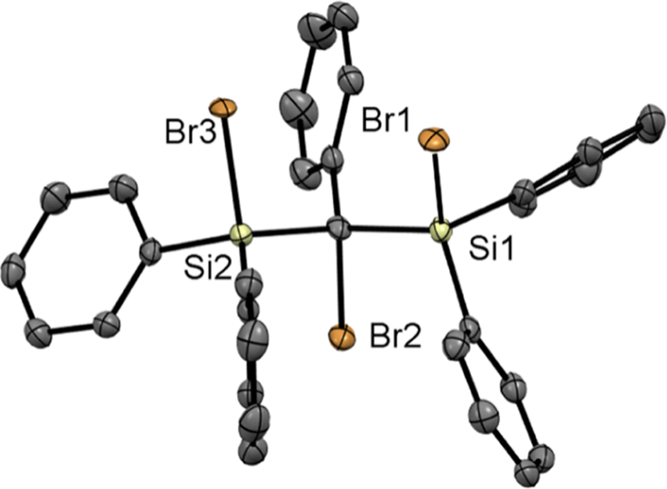
ORTEP representation for compound **3d**. Thermal ellipsoids are depicted at the 50% probability level. In compound **3d**, substitutional disorder for the halide atoms (Cl/Br) bound to silicon atoms was refined using 45/55 split positions. Hydrogen and chlorine atoms are omitted for clarity. Selected bond lengths (Å) and bond angles (deg) with estimated standard deviations: Si(1)–C(1) 1.916(2), Si(2)–C(1) 1.919(2), C(1)–Br(2) 2.006(19), Si(1)–Cl(1) 2.040(11), Si(1)–Br(1) 2.260(4), Si(2)–Br(3) 2.260(5), C(1)–Si(1)–Br(1) 108.12(4), C(1)–Si(2)–Br(3) 104.80(18), Si(1)–C(1)–Si(2) 117.63(10), Si(1)–C(1)–Br(2) 100.40(9), Si(2)–C(1)–Br(2) 102.28(9).

The molecular structure of **3d** was determined by single-crystal X-ray crystallography. Here, we could grow single crystals by cooling concentrated solutions in *n*-heptane to −30 °C (Figure 1). Compound **3d** crystallized in the monoclinic space group *P*2_1_/*n* with unexceptional bond lengths and angles. The unit cells contain four molecules (see the Supporting Information). Here the Si–Si bond was already broken. Additionally, this structure indicates that also a partial chlorination through carbon tetrachloride occurred. Prolonged reaction times, however, lead to the formation of an even more complex mixture and thus to the degradation of the desired target compound **3b**. Furthermore, fractionated crystallization was also not successful, and therefore, the raw product was used for the bromine substitution experiments without further purification.

### Bromine Substitution

The final step in this reaction pathway was the substitution of the bromines of species **3a**,**b** to obtain the corresponding acylsilanes. According to the literature, 1,1-dibromobenzyl substituted silanes can be either converted by silver salts, like silver acetate and silver trifluoroacetate (method A)^21^ or on silica gel (method B)^22^ to the acylsilanes.

### Method A

**Scheme 6:**
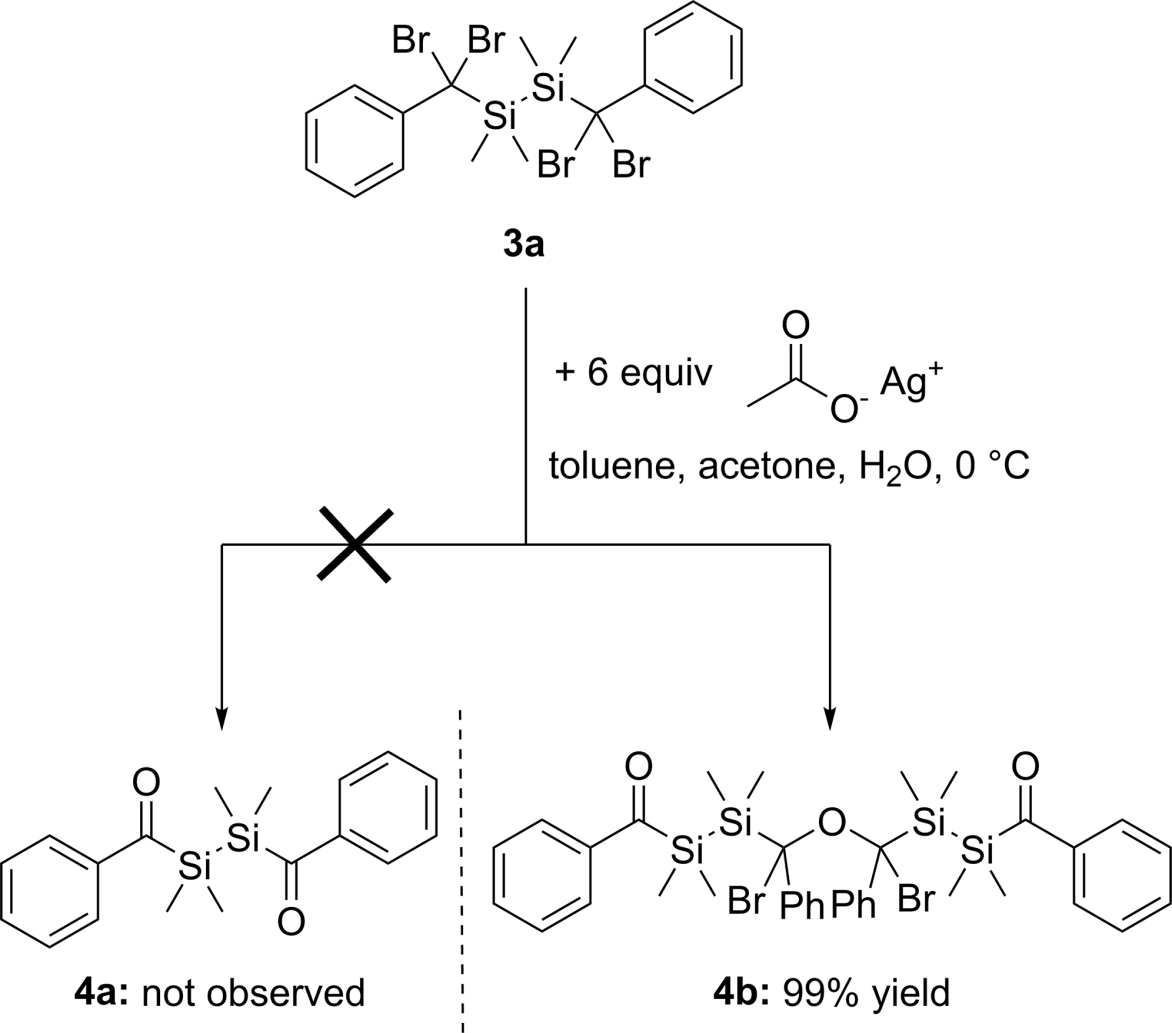
Substitution of the Bromines of **3a**

Compound **3a** and the reaction mixture of **3b−d** were reacted with an excess of silver acetate in a mixture of toluene, water, and acetone at 0 °C. Again, the result of the reaction is intensely influenced by the substituent at the silicon atoms. Surprisingly, the bromine substitution of **3a** does not lead to the expected bisacyldisilane **4a**, but instead the diastereomer **4b** was formed. Compound **4b** was obtained in isolated yields of 99% as a yellow oil (see Scheme 6).

**Scheme 7:**
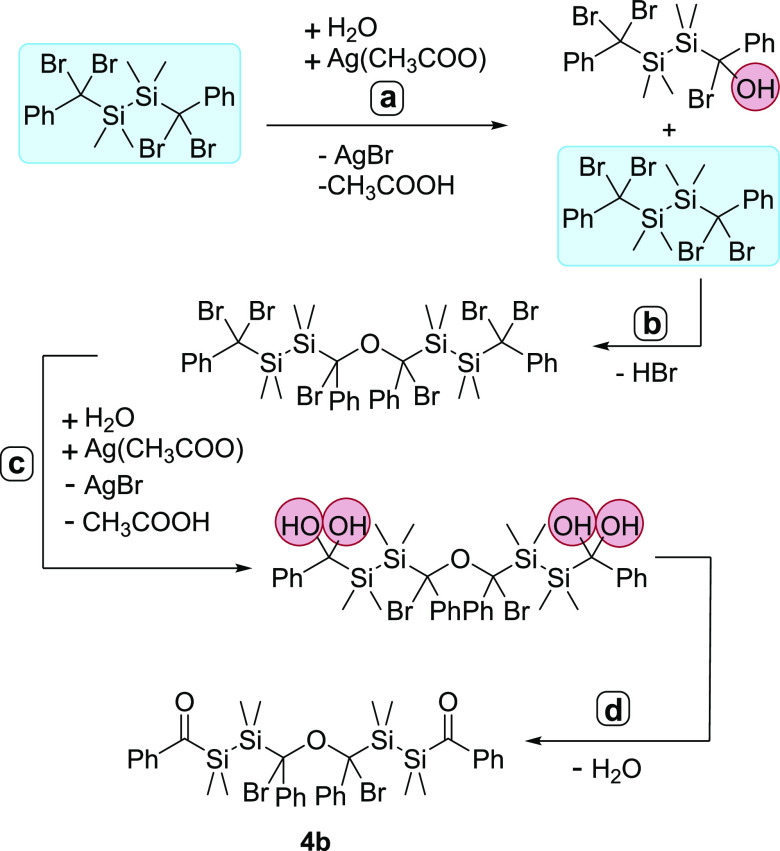
Proposed Reaction Pathway for the Formation of **4b**

The formation of compound **4b** indicates a more complex hydrolysis reaction. Scheme 7 depicts an assumption for a possible sequence. In the first step (**a**) a nucleophilic substitution takes place, resulting in the formation of silver bromide (AgBr). Subsequently, instead of the desired diol, dimerization occurs (**b**). The hypothesis is based on the circumstance that the reaction is selective and does not lead to any detectable byproducts, which would be expected in a simple stoichiometric competition with water. In the next step (**c**), the other bromines are substituted as expected and an intermediary geminal diol is formed. In step (**d**), the diastereomer **4b** is formed by the release of water. In addition, using silver trifluoroacetate instead of silver acetate, the same outcome of the reaction was observed.

### Method B

The same holds true for the use of silica gel to substitute the bromine atoms. Performing column chromatography with **3a** results in the formation of **4b** in lower yields (>75%). Due to the fact that the compound is a diastereomer, the ^1^H NMR spectrum and the ^13^C NMR spectrum of **4b** show four identical signals between 0.19 and 0.58 ppm in the ^1^H NMR spectrum and from −2.81 to 1.28 ppm in the ^13^C NMR spectra for the methyl groups on the two silicon atoms.

**Scheme 8:**
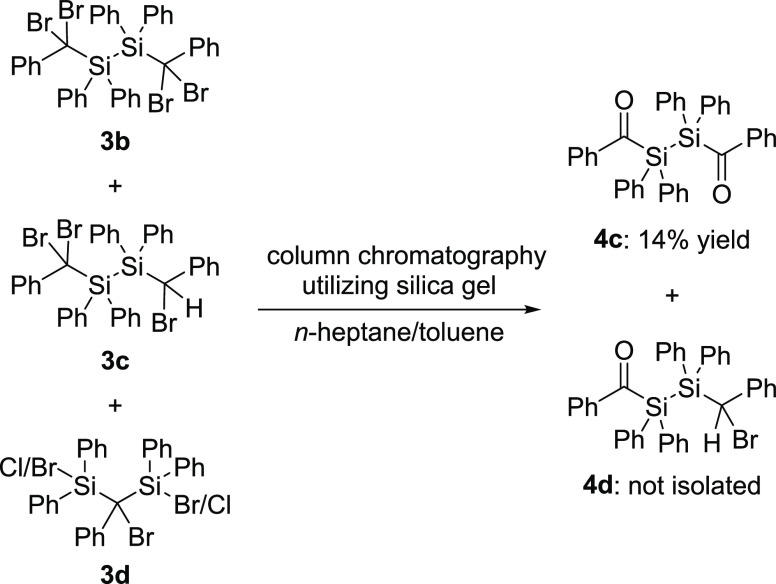
: Substitution of Bromines to Obtain **4c**

For the formation of **4c**, the product mixture of the bromination of **2b** (see Scheme 5) was treated with silica gel to simultaneously substitute the bromine and separate the product from impurities. Hereby, besides the formation of an uncharacterizable polymer and the target compound **4c**, a crystal of the expected monosubstituted byproduct **4d** could be obtained from an impure fraction (compare Scheme 8 and Figure 3). It should be pointed out that also method A leads to similar product formation. However, for the isolation of the target compound **4c**, a subsequent column chromatography is needed, and this consequently lowers the yield.

**Figure 2:**
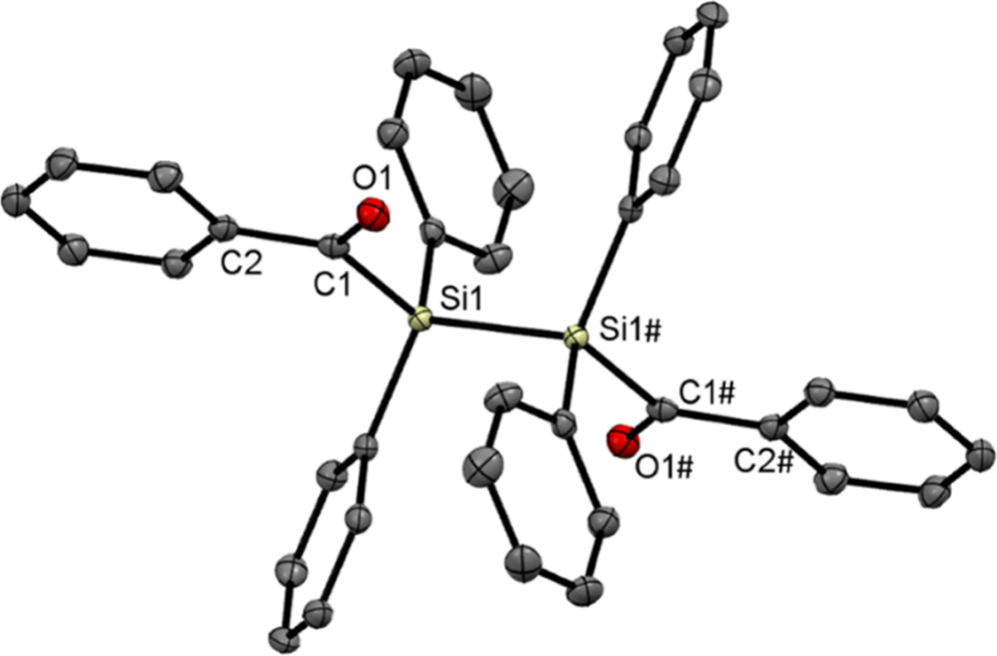
ORTEP representation for compound **4c**, whereby the co-crystalline solvent benzene is omitted for clarity. Thermal ellipsoids are depicted at the 50% probability level. Hydrogen atoms are omitted for clarity. Selected bond lengths (Å) and bond angles (deg) with estimated standard deviations: Si(1)–Si(1#) 2.358(7), Si(1)–C(1) 1.953(14), C(1)–O(1) 1.230(17), C(1)–C(2) 1.497(19), C(1)–Si(1)–Si(1#) 104.50(5), C(2)–C(1)–Si(1) 122.57(10), O(1)–C(1)–C(2) 119.85(12), O(1)–C(1)–Si(1) 117.58(10).

**Figure 3:**
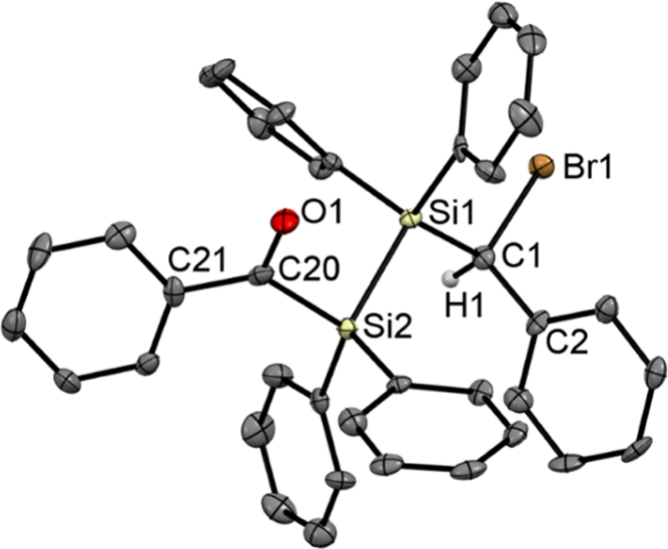
ORTEP representation for the monosubstituted byproduct **4d**. Thermal ellipsoids are depicted at the 50% probability level. Hydrogen atoms, except the hydrogen next to the C–Br, are omitted for clarity. Selected bond lengths (Å) and bond angles (deg) with estimated standard deviations: Br(1)–C(1) 1.971(7), C(1)–Si(1) 1.925(7), Si(1)–Si(2) 2.379(3), Si(2)–C(20) 1.951(7), C(20)–O(1) 1.235(8), C(20)–C(21) 1.495(10), Br(1)–C(1)–H(1) 106.2, Si(1)–C(1)–Br(1) 108.1(3), C(1)–Si(1)–Si(2) 108.4(2), C(20)–Si(2)–Si(1) 100.2(2), O(1)–C(20)–Si(2) 111.9(5), O(1)–C(20)–C(21) 120.0(6).

Single crystals of **4c** for X-ray analysis were grown in concentrated solutions of acetone at −30 °C. Additionally, as outlined above, single crystals for X-ray analysis of the monosubstituted byproduct **4d** were grown by cooling concentrated solutions in Et_2_O to −30 °C. The molecular structures are depicted in Figures 2 and 3. Compound **4c** crystallized in the triclinic space group *P*1̅ and compound **4d** in the monoclinic space group *P*2_1_/c with unexceptional bond lengths and angles. The unit cells contain one molecule for **4c** and eight molecules for **4d** (see the Supporting Information).

### Pathway II (Corey–Seebach Approach)

**Scheme 9:**
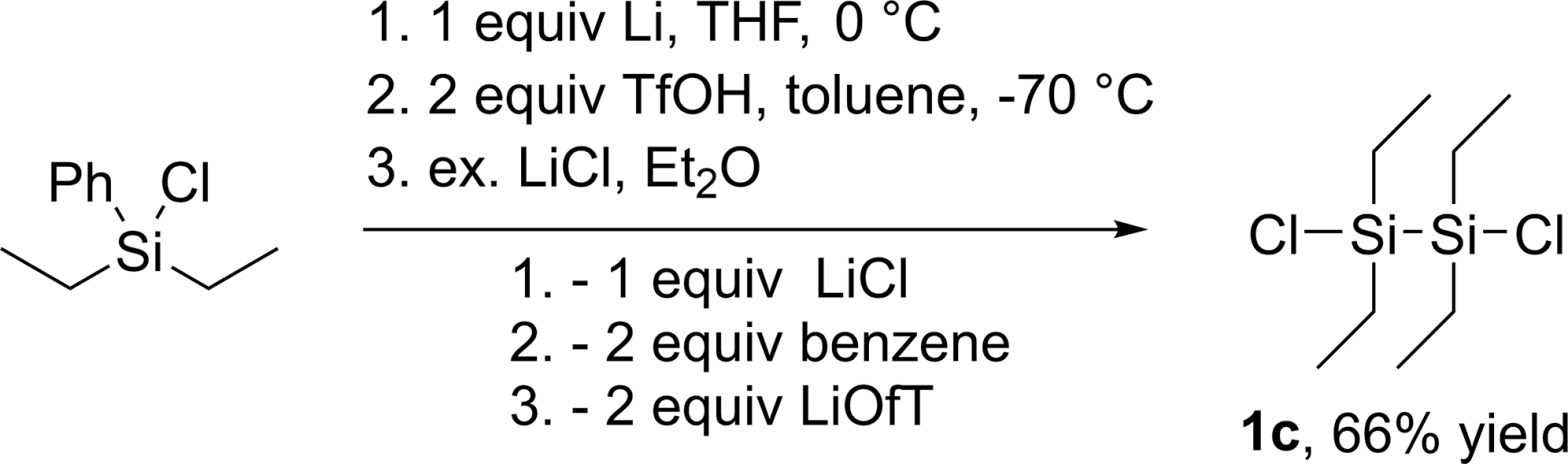
Synthesis of **1c**

**Scheme 10:**
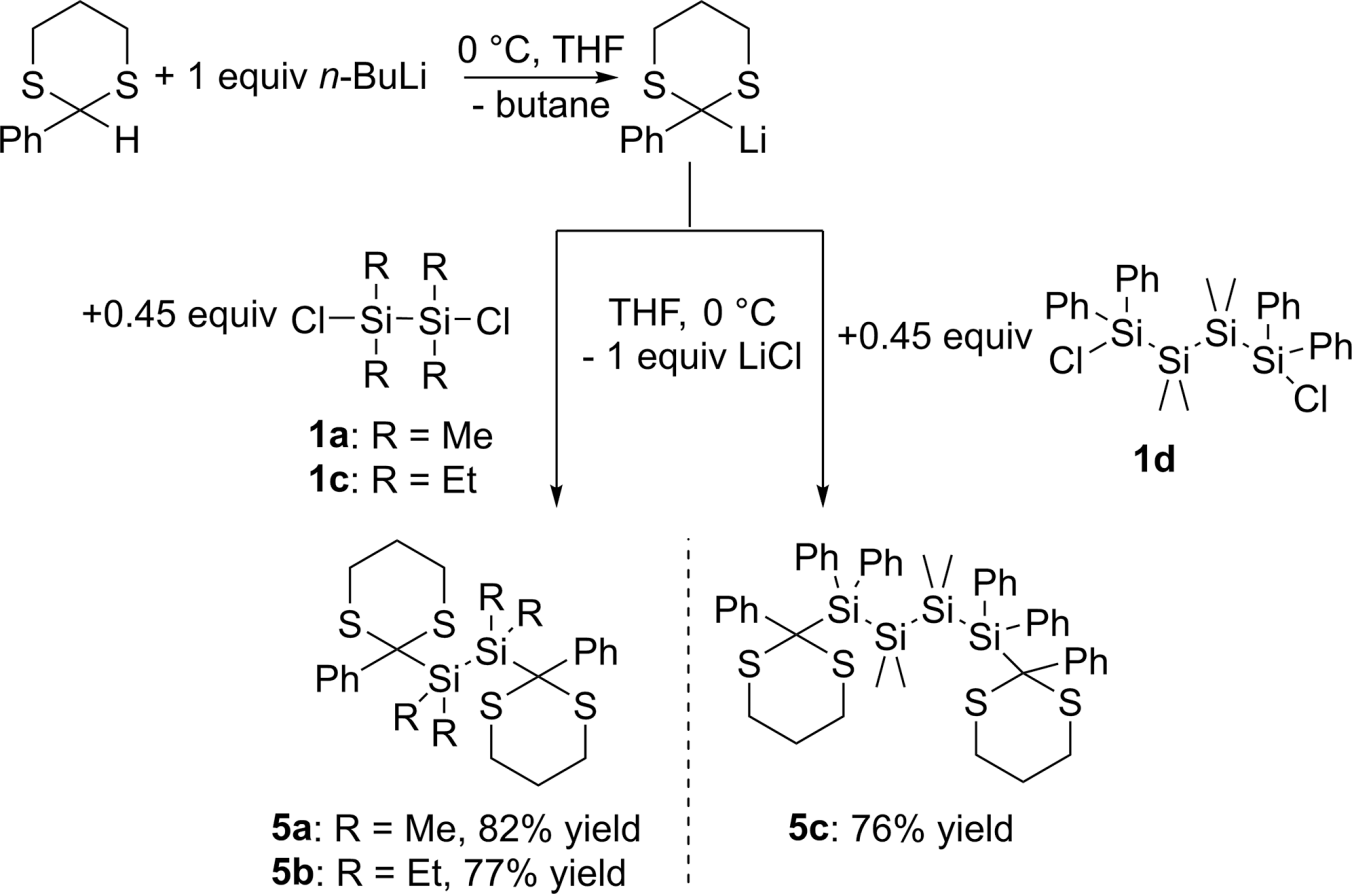
Synthesis of **5a–c**

Alternatively, the desired bisacylpolysilanes can be obtained via the well-known Corey–Seebach reaction. First, the 2-phenyl-1,3-dithiane is synthesized according to literature procedure^23^ and subsequently lithiated and reacted with α,ω-dihalogenated polysilanes **1a** and **1c**,**d** (see Scheme 10). 1,2-Dichloro-1,1,2,2-tetraethyldisilane **1c** was synthesized starting from chlorodiethyl(phenyl)silane by first converting it to the corresponding disilane through Wurtz-type coupling. Subsequently, 1,1,2,2-tetraethyl-1,2-diphenyldisilane was reacted with 2 equiv of trifluoromethanesulfonic acid (TfOH) in toluene at −70 °C to the corresponding bistriflate. This was followed by an in situ reaction with an excess of lithium chloride via nucleophilic substitution to the chlorosilane **1c** (compare Scheme 9). Compound **1d** was synthesized according to the literature procedure.^24^

To obtain compounds **5a–c**, the dithioketal is first lithiated at 0 °C in THF by *n*-butyllithium (*n*-BuLi) and then in situ reacted with the corresponding α,ω-dichlorosilanes **1a** and **1c**,**d** at 0 °C in THF for 2 h (see Scheme 10). Compounds **5a**–**c** are isolated in good yields by recrystallization from acetone at −30 °C. Spectroscopic and analytical data, which well support the structural assignment are summarized in the Experimental Section together with the experimental details. Characteristic ^13^C chemical shifts for the carbon atom attached to the dithioketal group of these compounds range from δ = 48.12 ppm to δ = 50.36 ppm.

**Scheme 11:**
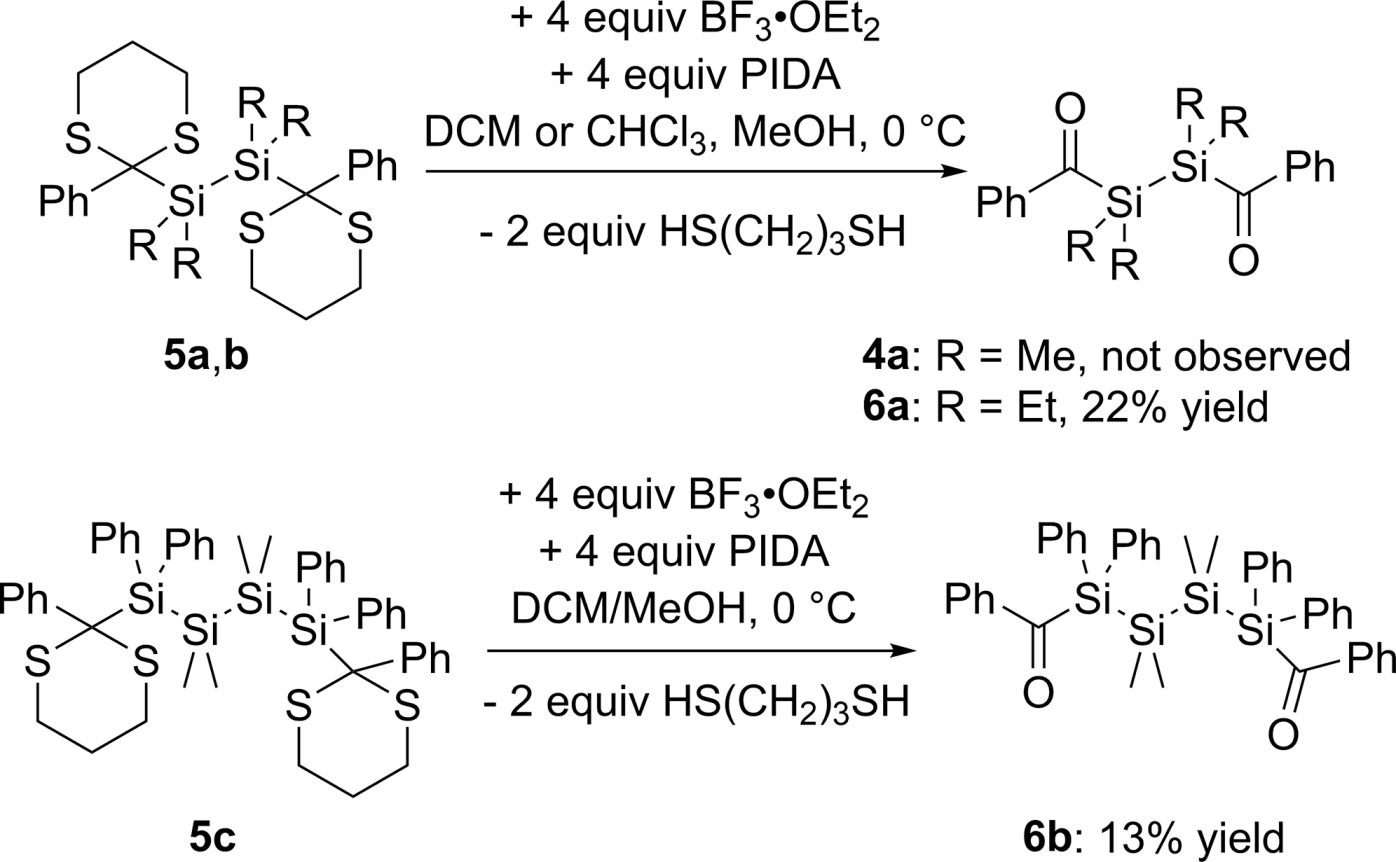
Synthesis of Bisacylpolysilanes **6a**,**b**

**Scheme 12:**
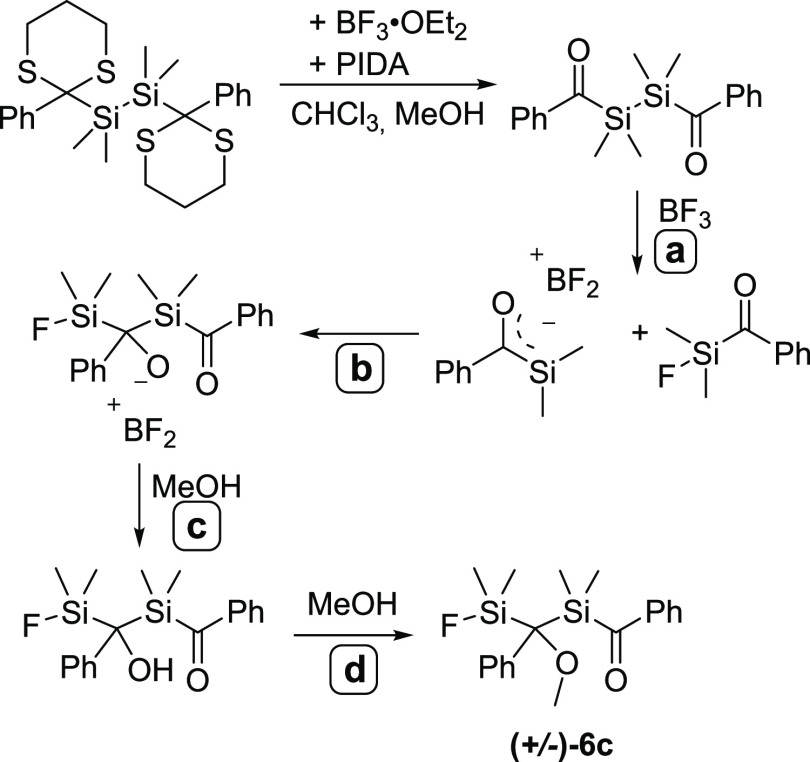
Proposed Mechanism for the Formation of **6c**

**Scheme 13:**
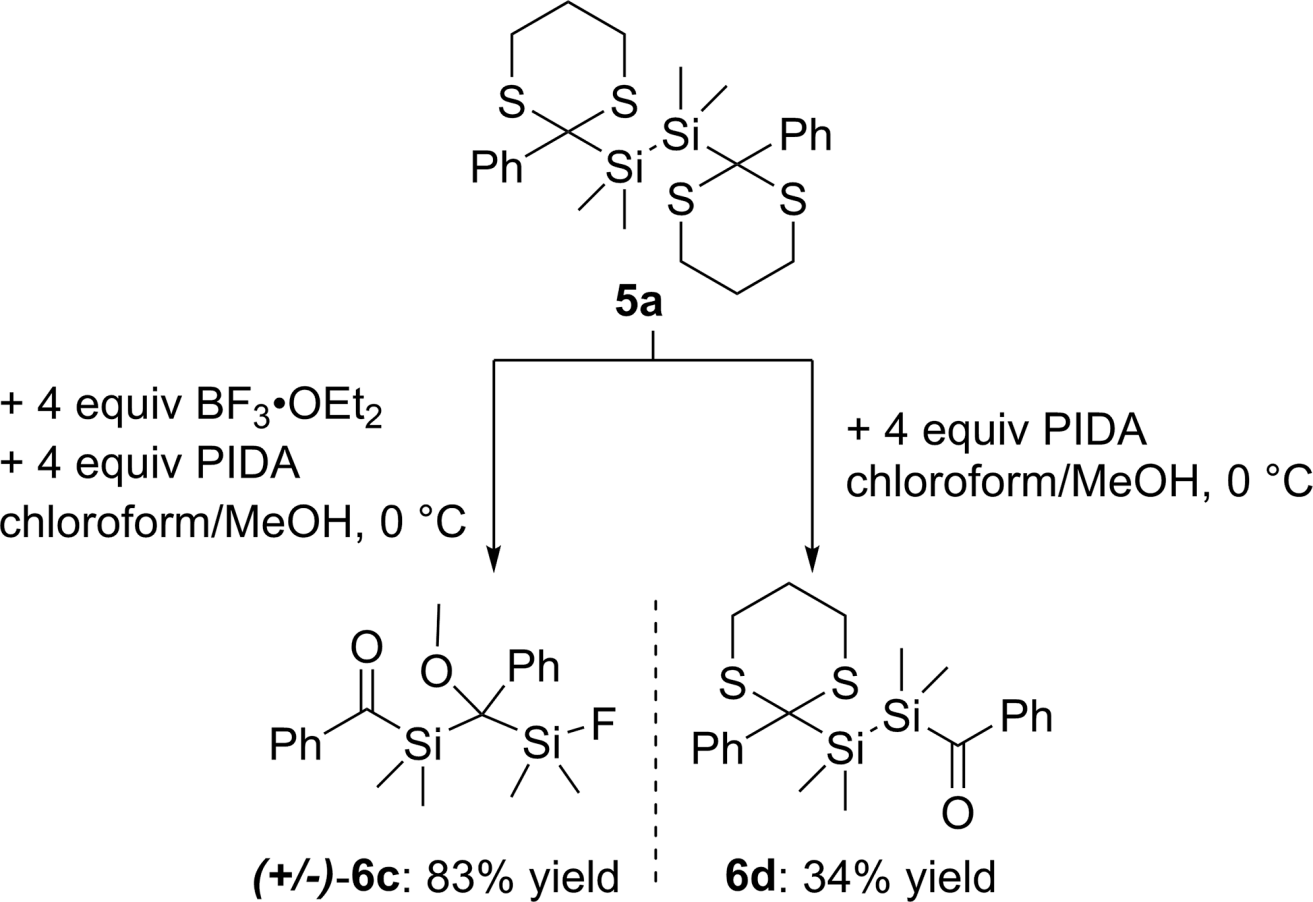
Dethioketalization of Compound **5a**

The dethioketalization is often described as the most difficult aspect of the Corey–Seebach route. After numerous different experiments, we figured out that the method described by Unterreiner, Barner-Kowollik, and co-workers^25^ is the only suitable procedure for this compound class. Utilizing this method, which was slightly adapted, compounds **5a–c** are placed in a mixture of dichloromethane and methanol and cooled to 0 °C. Subsequently, these compounds were reacted with 4 equiv of (diacetoxyiodo)benzene (PIDA) as oxidant and boron trifluoride diethyl etherate (BF_3_·OEt_2_) as catalyst (compare Scheme 11). In the case of **5b** and **5c**, the only selective products formed are the desired bisacylpolysilanes **6a** and **6b**. Since the deprotection is not particularly selective despite the optimized synthesis route, the yields are quite low. It should also be mentioned that the weak Si–Si bond is likely cleaved in this process, which reduces the yield. Spectroscopic and analytical data, which support the structural assignment, are summarized in the Experimental Section together with the experimental details. Characteristic ^13^C chemical shifts for the carbonyl-C atom of these compounds range from δ = 235.17 ppm to δ = 232.76 ppm. Surprisingly, the dethioketalization of **5a** does not lead to the expected bisacylpolysilane **4a** (compare Scheme 11). Instead, a rearrangement cascade takes place and a racemic mixture of compound **6c** is formed. Hereby, we assume that the actual product **4a** is formed, but immediately BF_3_ induces a Si–Si bond cleavage, resulting in the formation of a fluoroacylsilane as well as a silenolate (**a**),^26^ which subsequently undergo an intermolecular sila-aldol reaction (**b**). In the presence of MeOH, this aldol product is protonated to the corresponding alcohol (**c**). In step **d**, the alcohol further reacts with another equivalent of MeOH to **6c** via the elimination of H_2_O (see Scheme 12). Furthermore, our proposed mechanism is strengthened by the fact that if the catalyst is omitted, only partial deprotection occurs and compound **6d** is formed in low yield (see Scheme 13).

**Figure 4:**
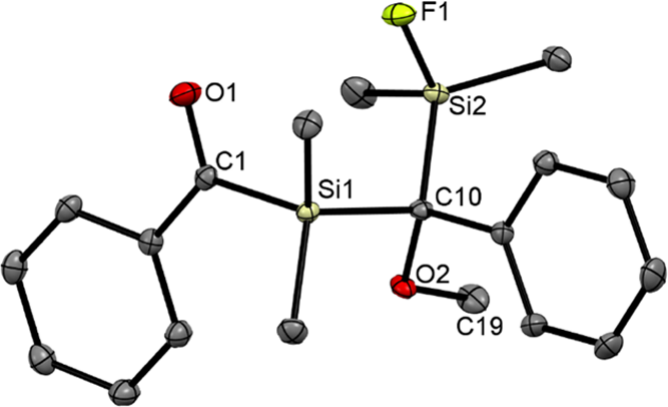
ORTEP representation for the (*S*)-enantiomeric **6c**. Thermal ellipsoids are depicted at the 50% probability level. Hydrogen atoms are omitted for clarity. Selected bond lengths (Å) and bond angles (deg) with estimated standard deviations: C(1)–O(1) 1.229(15), Si(1)–C(1) 1.946(12), Si(1)–C(10) 1.924(13), C(10)–O(2) 1.462(13), O(2)–C(19) 1.427(16), C(10)–Si(2) 1.925(12), Si(2)–F(1) 1.611(8), O(1)–C(1)–Si(1) 114.48(9), C(1)–Si(1)–C(10) 107.13(5), Si(1)–C(10)–O(2) 100.41(7), Si(2)–C(10)–O(2) 110.70(7), Si(1)–C(10)–Si(2) 111.87(6), C(10)–Si(2)–F(1) 104.90(5).

Single crystals of compound **6c** suitable for X-ray structural analysis were grown by cooling concentrated solutions of acetone to −30 °C. The compound also crystallizes as a racemic mixture, as the unit contains both enantiomers. For visualization, we depicted the *S*-enantiomer (Figure 4). The unit cells contain four molecules (see the Supporting Information).

### UV–vis Spectroscopy

**Figure 5:**
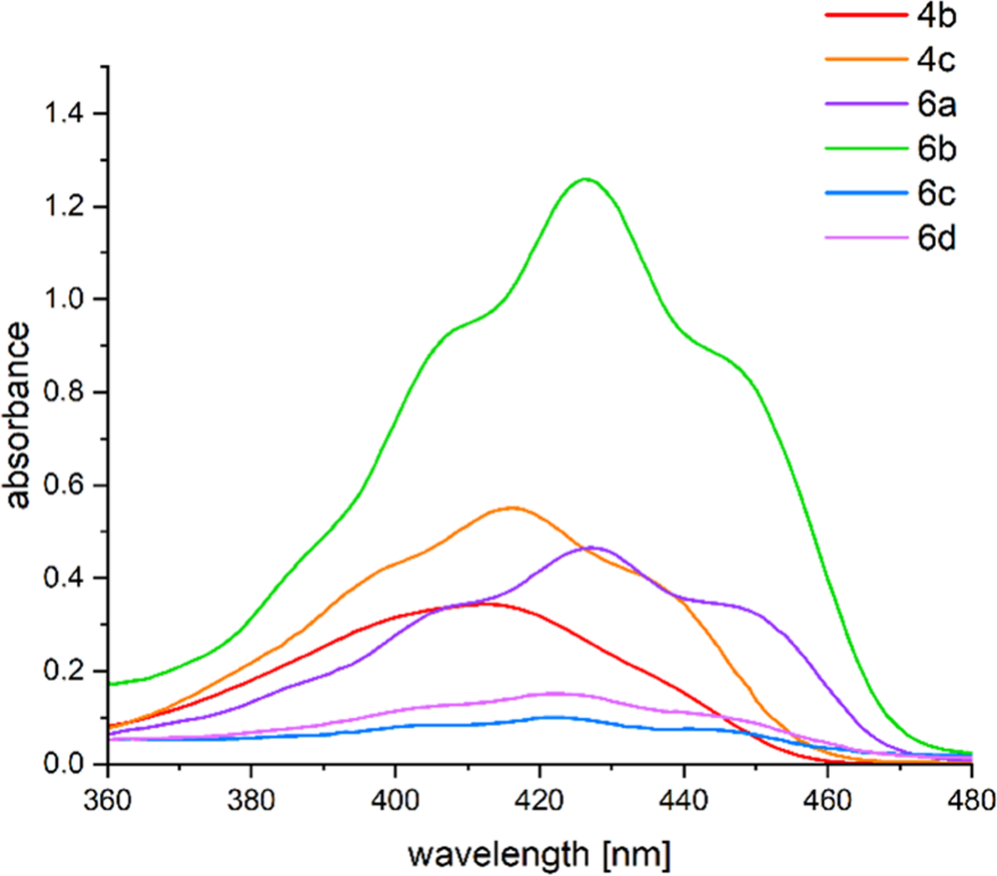
UV–vis absorption spectra of compounds **4b**,**c** and **6a–d** (measured in toluene solution, *c* = 10^–3^ mol L^–1^).

For the determination of the absorption properties of our synthesized bisacylpolysilanes and derivatives **4b**,**c** and **6a**–**d**, their UV–vis spectra were measured (see Figure 5). All derivatives have nearly identical λ_max_ values between 412 and 428 nm. These absorption bands correspond to the well-known *n*/σ-π* transitions from the carbonyl lone pairs and the Si–Si σ-bonds to both acyl moieties.^2^ Surprisingly, compound **6b** shows a significantly higher extinction coefficient compared to the other compounds.

### Photolysis Experiments

To investigate the photochemical pathway of these compounds, we selected two representatives, **4b** and **4c**, and irradiated them at a wavelength of 405 nm in the presence of methanol and triethylamine. Here, we used methanol and triethylamine to trap instable carbenes, which were formed via Norrish type II pathways. According to NMR spectroscopy and thin-layer chromatography, several products were present in the reaction mixtures after photolysis was completed. We were able to detect at least eight photoproducts for the photolysis reaction of compounds **4b** and **4c**, recognizable by the number of peaks in the thin-layer chromatography and by the signals in the ^29^Si NMR spectra as well as by the methoxy signals in the ^1^H NMR and ^13^C NMR spectra (see Figures S42–S47). However, we were unable to isolate any of these products despite several attempts of separation and purification. On the one hand, we found nearly identical *R*_f_ values of the photoproducts, and on the other hand, we detected that some of these compounds react with silica gel resulting in even more complex mixtures. Based on the NMR data after the photolysis of compound **4b**, we observed in the ^1^H NMR spectrum the presence of several chemical shifts characteristic of methoxy groups indicating Norrish type II pathways. Interestingly, we also detected the formation of benzaldehyde, which is characteristic of Norrish type I pathways. Additionally, in the ^29^Si NMR spectrum, multiple signals characteristic of Si atoms attached to oxygen are found. Furthermore, signals characteristic of the disilane moieties nearly vanished, which also indicates radical fragmentation. Contrary to this, compound **4c** shows no benzaldehyde formation, but again multiple signals characteristic of methoxy groups are present. Moreover, the ^29^Si NMR spectrum indicates that the Si–Si bond is still present, as multiple signals can be found in the characteristic region around δ = −20 to δ = −35 ppm. This shows that for **4c**, Norrish type II pathways are significantly more favored.

**Figure 6:**
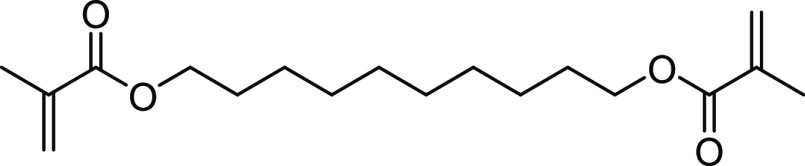
Structure of 1,10-decanediol dimethacrylate (D_3_MA).

**Figure 7:**
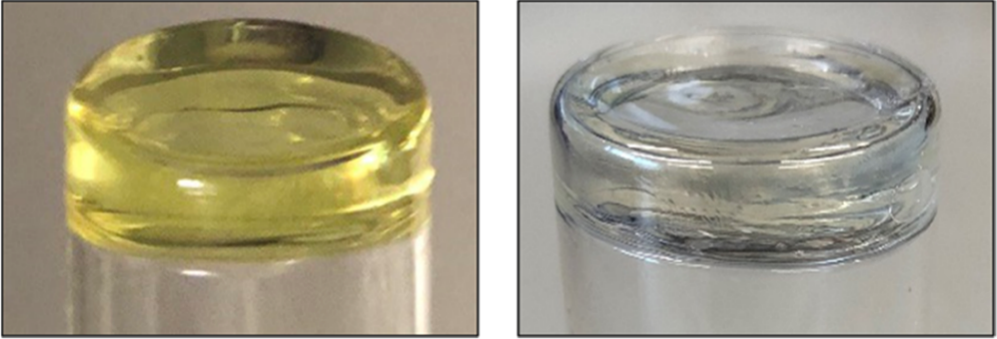
Left:1 wt % of compound **4b** dissolved in D_3_MA directly after curing. Right: several days after irradiation.

The same photochemistry was found by irradiating **4b** and **4c** in the presence of acrylates. For this purpose, we dissolved 1 wt % of each compound in 2 g of a suitable methacrylate, in our case 1,10-decanediol dimethacrylate (D_3_MA, shown in Figure 6). The samples were irradiated with blue light-emitting diode (LED) light using a so-called Bluephase lamp from Ivoclar Vivadent AG (1200 mW cm^–2^). Compound **4b** partially cured after 2 min, and another 4 min later, the polymer showed complete curing, reaching a curing depth of approximately 1 cm. After this period of irradiation, however, the polymer was still yellow, but lost its color a few days later by photobleaching, as shown in Figure 7. Compound **4c**, on the other hand, barely cured after 2 min, and even after a further irradiation, little to no complete curing was detected.

## Conclusions

In summary, a variety of α,ω-bisacylpolysilanes were synthesized via two synthetic pathways. The bromine substitution reaction was the first investigated method to obtain these compounds. The second protocol was the Corey–Seebach approach to isolate other derivatives of this kind. On the basis of this investigation, we found that the selectivity towards the target molecules is significantly affected by the substituent at the α-silicon atoms. In the case of methyl groups attached to the α-silicon atoms, hitherto unknown reactions (i.e., intermolecular sila-aldol reaction) take place that yield more complex acylsilanes **4b** and **6c**. The more sterically hindered ethyl and phenyl groups attached to the α-silicon atoms enable the formation of the expected bisacylpolysilanes **4c**, **6a**, and **6b**. All isolated compounds were analyzed by a combination of NMR, Infrared (IR), and UV–vis spectroscopy as well as mass spectrometry. Furthermore, the photochemical pathway of two representative examples (**4b**,**c**) was studied. In contrast to the recently published bisacyldigermane counterparts,^27^ which only follow Norrish type I, the bisacylpolysilanes follow both Norrish-type mechanisms, excluding them from applicability, but simultaneously paving the way for further investigations concerning potential photoinitiators.

## Experimental Section

### General Considerations

All experiments with air- or moisture-sensitive compounds were carried out under inert conditions using standard Schlenk techniques. Solvents were dried using a column solvent purification system.^28^ All chemicals from commercial sources were used as purchased from chemical suppliers. The used benzylchlorodimethylsilane,^29^ 2-phenyl-1,3-dithiane^23^ chlorodiethyl(phenyl)silane^19^ and 1,4-dichloro-2,2,3,3-tetramethyl-1,1,4,4-tetraphenyltetrasilane^24^ were produced according to the corresponding literature. ^1^H-, ^13^C-, ^19^F-, and ^29^Si- NMR spectra were recorded on a Varian INOVA 300, a 200 MHz Bruker AVANCE DPX, or a Bruker Avance 300 MHz spectrometer in C_6_D_6_ or CDCl_3_ solution and were referenced vs tetramethylsilane (TMS) using the internal ^2^H-lock signal of the solvent. Mass spectra were obtained either with a Kratos Profile mass spectrometer or with a Q-TOF Premier from Waters, Manchester, England. Therefore, the original electrospray ionisation (ESI) source of the instrument was replaced by a standard LIFDI source from Linden CMS, Weyhe, Germany. Infrared spectra were obtained on a Bruker α-P Diamond ATR spectrometer from the solid sample. Melting points were determined using a Stuart SMP50 apparatus and are uncorrected. Elemental analyses were carried out on a Hanau Vario Elementar EL apparatus. UV–vis spectra were recorded with an Agilent Cary 60 UV–vis spectrometer.

### X-ray Crystallography

All crystals suitable for single-crystal X-ray diffractometry were removed from a vial or Schlenk flask and immediately covered with a layer of silicone oil. A single crystal was selected, mounted on a glass rod on a copper pin, and placed in a cold N_2_ stream. X-ray diffraction (XRD) data collections for compounds **3a**, **3d**, **4c**, **4d**, and **6c** were performed on a Bruker APEX II diffractometer with the use of an Incoatec microfocus sealed tube of Mo Kα radiation (λ = 0.71073 Å) and a charge-coupled device (CCD) area detector. Empirical absorption corrections were applied using SADABS or TWINABS.^30,31^ The structures were solved with either the use of direct methods or the intrinsic phasing option in SHELXT and refined by the full-matrix least-squares procedures in SHELXL.^32−34^ or Olex2.^35^ The space group assignments and structural solutions were evaluated using PLATON.^36,37^ Nonhydrogen atoms were refined anisotropically. Hydrogen atoms were either located in a difference map or in calculated positions corresponding to standard bond lengths and angles. Disorder was handled by modeling the occupancies of the individual orientations using free variables to refine the respective occupancy of the affected fragments (PART).^38^ In compound **3d**, substitutional disorder for the halide atoms (Cl/Br) bound to silicon atoms was refined using 45/55 split positions. Table S1 in the Supporting Information contains crystallographic data and details of measurements and refinement for all compounds. Crystallographic data (excluding structure factors) have been deposited with the Cambridge Crystallographic Data Centre (CCDC) under the following numbers: **3a**, 2173935; **3d**, 2173933; **4c**, 2173931; **4d**, 2173932; and **6c**, 2173934.

### Synthesis

#### Synthesis of 1,2-Dichloro-1,1,2,2-tetraphenyldisilane (**1b**)

Hexaphenyldisilane (40.00 g, 77.10 mmol) was dissolved in toluene, which was then cooled to −70 °C. Trifluoromethanesulfonic acid (14.38 mL, 161.91 mmol) was added. As soon as the reaction mixture reached room temperature, it was further heated with a heat gun to achieve complete dissolution of the reactant. Once the reaction mixture reached room temperature again, 32.69 g of LiCl (771.06 mmol) were added. To dissolve the LiCl, some diethyl ether was added and an overnight stirring of the reaction mixture followed. The volatile components were removed under vacuum. To remove the salts, the product was dissolved in toluene and filtered. The volatile components were again removed under vacuum. Recrystallization from *n*-heptane at −30 °C gave the product in 89% yield (30.0 g) as a white solid. The analytical data is identical to the reported data.^39^

#### Synthesis of 1,2-Dibenzyl-1,1,2,2-tetramethyldisilane (**2a**)

##### Method A

Magnesium (14.07 g, 578.68 mmol) was added to a three-neck flask equipped with a reflux condenser and a dropping funnel. Tetrahydrofuran (300 mL) was added under nitrogen flow. In the dropping funnel, 32.96 mL of benzyl bromide (275.56 mmol) and tetrahydrofuran were added and stirred. About 10% of this mixture was subsequently added to the flask containing the magnesium/tetrahydrofuran. To start the Grignard reaction, the suspension was heated using a heat gun. The beginning of the reaction is characterized by a slight turbidity and strong heat development. The dropping speed is selected in a way to ensure that the reaction mixture remains at reflux temperature. After the end of the addition, the reflux temperature is maintained for another 2 h. The so-obtained Grignard reagent was further added to a solution of 12.90 g of 1,2-dichloro-1,1,2,2-tetramethyldisilane (68.89 mmol) in tetrahydrofuran at 0 °C. Once the addition was complete, it was stirred for several days. The reaction mixture was further aqueously worked up with a saturated NH_4_Cl solution. The organic layer was separated from the aqueous layer. Then, the aqueous layer was extracted three times with dichloromethane. After drying the combined organic phases over Na_2_SO_4_, the volatile components were removed under vacuum. Recrystallization from acetone at −30 °C gave the product in 86% yield (17.60 g) as a white solid.

##### Method B

Lithium (1.13 g, 162.82 mmol) and tetrahydrofuran were placed in a two-neck flask and cooled to 0 °C. After adding 30.08 g of benzylchlorodimethylsilane (162.82 mmol), the reaction mixture was stirred for several days at room temperature. NMR spectroscopy was performed to ensure complete conversion. The reaction mixture was further aqueously worked up with a 10% H_2_SO_4_ solution. The organic layer was separated from the aqueous layer. Then, the aqueous layer was extracted 3 times with diethyl ether. After drying the combined organic phases over Na_2_SO_4_, the volatile components were removed under vacuum. Recrystallization from acetone at −30 °C gave the product in 81% yield (39.30 g) as a white solid. Data for **2a** are as follows. *M*_p_: 39–42 °C. Elem. Anal. calcd for C_18_H_26_Si_2_: C, 72.41%; H, 8.78%. Found C, 72.65%; H, 8.69%. ^1^H NMR (300 MHz, C_6_D_6_) δ 7.14 (t, *J* = 7.6 Hz, 4H, Aryl-*H*), 6.99 (t, *J* = 7.2 Hz, 2H, Aryl-*H*), 6.91 (d, *J* = 7.3 Hz, 4H, Aryl-*H*), 2.01 (s, 4H, −C*H*_2_−), 0.04 (s, 12H, −C*H*_3_). ^13^C NMR (76 MHz, C_6_D_6_) δ 140.53, 128.59, 128.51, 124.56 (Aryl-*C*), 25.10 (−*C*H_2_), −3.85 (-Si-(*C*H_3_)_2_). ^29^Si NMR (60 MHz, C_6_D_6_) δ −17.08 (*Si*–Me_2_). High resolution mass spectrometry (HRMS): (LIFDI^+^) calcd for [C_18_H_26_Si_2_]^+•^ (M^+•^): 298.1573. Found: 298.1579.

#### Synthesis of 1,2-Dibenzyl-1,1,2,2-tetraphenyldisilane (**2b**)

Magnesium (14.07 g, 578.68 mmol) was added to a three-neck flask, which was equipped with a reflux condenser and a dropping funnel. Tetrahydrofuran (300 mL) was added under nitrogen flow. In the dropping funnel, 32.96 mL of benzyl bromide (275.56 mmol) and tetrahydrofuran were added and stirred. About 10% of this mixture was subsequently added to the flask containing the magnesium/tetrahydrofuran. To start the Grignard reaction, the suspension was heated using a heat gun. The beginning of the reaction is characterized by slight turbidity and strong heat development. The dropping speed is selected in a way to ensure that the reaction mixture remains at reflux temperature. Once the addition has ended, the reflux temperature is maintained for another 2 h. The Grignard reagent obtained was then added at 0 °C to 30.00 g of 1,2-dichloro-1,1,2,2-tetraphenyldisilane (68.89 mmol) dissolved in tetrahydrofuran. Once the addition was complete, it was stirred for several days. The reaction mixture was further aqueously worked up with a saturated NH_4_Cl solution. The organic layer was separated from the aqueous layer. Then, the aqueous layer was extracted 3 times with dichloromethane. After drying the combined organic phases over Na_2_SO_4_, the volatile components were removed under vacuum. Recrystallization from toluene/*n*-heptane at −30 °C gave the product in 83% yield (31.3 g) as a white solid. Data for **2b** are as follows. *M*_p_: 134–137 °C. Elem. Anal. calcd for C_38_H_34_Si_2_: C, 83.46%; H, 6.27%. Found C, 83.76%; H, 6.00%. ^1^H NMR (300 MHz, C_6_D_6_) δ 7.38 (d, *J* = 7.4 Hz, 8H, Aryl-*H*), 7.14–7.03 (m, 12H, Aryl-*H*), 7.00–6.91 (m, 6H, Aryl-*H*), 6.85 (d, *J* = 6.3 Hz, 4H, Aryl-*H*), 2.79 (s, 4H, −C*H*_2_−). ^13^C NMR (76 MHz, C_6_D_6_) δ 139.23, 136.77, 135.35, 129.59, 129.51, 128.49, 128.06, 125.02 (Aryl-*C*), 23.78 (−*C*H_2_−). ^29^Si NMR (60 MHz, C_6_D_6_) δ −19.86 (−*Si*–Ph_2_). HRMS: (LIFDI^+^) calcd for [C_38_H_34_Si_2_]^+•^ (M^+•^): 546.2199. Found: 546.2374.

#### Synthesis of 1,2-Bis(dibromo(phenyl)methyl)-1,1,2,2-tetramethyldisilane (**3a**)

1,2-Dibenzyl-1,1,2,2-tetramethyldisilane (20.00 g, 66.98 mmol) was dissolved in carbon tetrachloride. *N*-Bromosuccinimide (47.69 g, 267.94 mmol) and finally 0.97 g of the radical initiator, benzoyl peroxide (4.02 mmol), were added. After refluxing the mixture overnight and cooling it to room temperature, the volatile components were removed under vacuum. The reaction mixture was further aqueously worked up with a saturated NH_4_Cl solution by absorbing the residue in dichloromethane. The organic layer was separated from the aqueous layer. Then, the aqueous layer was extracted three times with dichloromethane. After drying the combined organic phases over Na_2_SO_4_, the volatile components were removed under vacuum. Washing with hot *n*-heptane gave the product in 72% yield (29.60 g) as a brownish-white solid. Data for **3a** are as follows. Mp: 148–150 °C. Elem. Anal. calcd for C_18_H_22_Br_4_Si_2_: C, 35.20%; H, 3.61%. Found C, 35.41%; H, 3.45%. ^1^H NMR (300 MHz, C_6_D_6_) δ 7.71 (d, *J* = 8.0 Hz, 4H, Aryl-*H*), 6.97 (t, *J* = 7.7 Hz, 4H, Aryl-*H*), 6.88 (t, *J* = 7.2 Hz, 2H, Aryl-*H*), 0.44 (s, 12H, −C*H*_3_). ^13^C NMR (76 MHz, C_6_D_6_) δ 142.58, 128.98 (Aryl-*C*), 66.27 (−*C*–Br_2_), 0.14 (−Si–(*C*H_3_)_2_). ^29^Si NMR (60 MHz, C_6_D_6_) δ 5.18 (−*Si*–Me_2_). HRMS: (LIFDI^+^) calcd for [C_9_H_11_Br_2_Si]^+^ (M–C_9_H_11_Br_2_Si^•^): 304.8997. Found: 306.9195.

#### Synthesis of ((Oxybis(bromo(phenyl)methylene))bis(1,1,2,2-tetramethyldisilane-2,1-diyl))bis(phenylmethanone) (**4b**)

To 3.26 g of silver acetate (19.54 mmol), some toluene was added and cooled to 0 °C. Acetone (20 mL), water (10 mL), and 1,2-bis(dibromo-(phenyl)methyl)-1,1,2,2-tetramethyldisilane (2.00 g, 3.26 mmol) were added. It was stirred at room temperature without nitrogen atmosphere for several days. The reaction mixture was further aqueously worked up with a saturated NH_4_Cl solution. The organic layer was separated from the aqueous layer. Then, the aqueous layer was extracted three times with diethyl ether. After drying the combined organic phases over Na_2_SO_4_, the volatile components were removed under vacuum. Alternatively, silver trifluoroacetate can be used instead of silver acetate, or column chromatography can be performed with silica gel to obtain the corresponding product. Without further purification, this resulted in the product formation of 99% yield (1.30 g) as a yellow oil. Data for **4b** are as follows. Elem. Anal. calcd for C_36_H_44_Br_2_O_3_Si_4_: C, 54.26%; H, 5.57%. Found C, 53.88%; H, 5.54%. ^1^H NMR (300 MHz, C_6_D_6_) δ 7.58 (dd, *J* = 15.8, 7.7 Hz, 8H, Aryl-*H*), 7.10–6.84 (m, 12H, Aryl-*H*), 0.58 (s, 6H, −C*H*_3_), 0.48 (s, 6H, −C*H*_3_), 0.36 (s, 6H, −C*H*_3_), 0.19 (s, 6H, −C*H*_3_). ^13^C NMR (76 MHz, C_6_D_6_) δ 237.25 (*C*=O), 142.03, 139.32, 132.77, 127.86, 127.64, 127.57, 127.24, 125.38 (Aryl-*C*), 52.78 (−Si–*C*–O−), 1.28, −0.85, −2.22, −2.81 (−Si–(*C*H_3_)_2_). ^29^Si NMR (60 MHz, CDCl_3_) δ 10.70, −5.66 (−*Si*–Me_2_). IR (neat): ν(C=O) 1575, 1610, 1590 cm^–1^. UV–vis: λ [nm], ε[L mol^–1^ cm^–1^] 412, 344. HRMS: calcd for [C_35_H_41_Br_2_O_3_Si_4_]^+^ (M–CH_3_^–^): 779.0500. Found: 779.0483.

#### Synthesis of (1,1,2,2-Tetraphenyldisilane-1,2-diyl)bis(phenylmethanone) (**4c**)

1,2-Dibenzyl-1,1,2,2-tetraphenyldisilane (2.00 g, 3.66 mmol) was dissolved in carbon tetrachloride. *N*-Bromosuccinimide (2.60 g, 14.63 mmol) and finally 0.05 g of the radical initiator, benzoyl peroxide (0.22 mmol), were added. After refluxing the mixture overnight and cooling it to room temperature, the volatile components were removed under vacuum. The reaction mixture was further aqueously worked up with a saturated NH_4_Cl solution by absorbing the residue in dichloromethane. The organic layer was separated from the aqueous layer. Then, the aqueous layer was extracted three times with dichloromethane. After drying the combined organic phases over Na_2_SO_4_, the volatile components were removed under vacuum. Gradual flash column chromatography (starting with *n*-heptane/toluene 4:1) gave the product in 14% yield (0.30 g) as a yellow solid. Data for **4c** are as follows. Mp: 174–176 °C. Elem. Anal. calcd for C_38_H_30_O_2_Si_2_: C, 79.40%; H, 5.26%. Found C, 79.13%; H, 5.21%. ^1^H NMR (300 MHz, C_6_D_6_) δ 7.93 (d, *J* = 7.0 Hz, 4H, Aryl-*H*), 7.82 (d, *J* = 1.8 Hz, 4H, Aryl-*H*), 7.80 (t, *J* = 2.4 Hz, 4H, Aryl-*H*), 7.06–7.01 (m, 12H, Aryl-*H*), 6.94–6.83 (m, 6H, Aryl-*H*). ^13^C NMR (76 MHz, C_6_D_6_) δ 230.89 (*C*=O), 142.56, 136.98, 133.16, 132.91, 130.02, 129.02, 128.61, 128.55 (Aryl-*C*). ^29^Si NMR (60 MHz, C_6_D_6_) δ −25.92 (−*Si*–Ph_2_). IR (neat): ν(C=O) 1572, 1607 cm^–1^. UV–vis: λ [nm], ε[L mol^–1^ cm^–1^] 400, 431; 416, 551; 434, 410. HRMS: (LIFDI^+^) calcd for [C_38_H_30_O_2_Si_2_]^+•^ (M^+•^): 574.1785. Found: 574.2210.

#### Synthesis of 1,1,2,2-Tetraethyl-1,2-diphenyldisilane

The synthesis was carried out according to the literature^19^ with slight adjustments as follows. Chlorodiethyl(phenyl)silane (32.94 g, 165.72 mmol) was dissolved in tetrahydrofuran and cooled to 0 °C. After the addition of 1.15 g of lithium (165.72 mmol), the reaction solution was stirred overnight at room temperature. The reaction mixture was further aqueously worked up with a saturated NH_4_Cl solution. The organic layer was separated from the aqueous layer. Then, the aqueous layer was extracted 3 times with diethyl ether. After drying the combined organic phases over Na_2_SO_4_, the volatile components were removed under vacuum. Distillation at 175 °C gave the product in 43% yield (11.61 g) as a colorless oil. The analytical data are identical to the reported data.^40^

#### Synthesis of 1,2-Dichloro-1,1,2,2-tetraethyldisilane (**1c**)

1,1,2,2-Tetraethyl-1,2-diphenyldisilane (11.61 g, 35.54 mmol) was dissolved in toluene, which was then cooled to −70 °C. Trifluoromethanesulfonic acid (6.63 mL, 74.64 mmol) was added. As soon as the reaction mixture reached room temperature, it was further heated with a heat gun to achieve complete dissolution of the reactant. Once the reaction mixture reached room temperature again, 15.07 g of LiCl (355.55 mmol) were added. To dissolve the LiCl, some diethyl ether was added and an overnight stirring of the reaction mixture followed. The volatile components were removed under vacuum. To remove the salts, the product was dissolved in *n*-pentane and filtered. The volatile components were again removed under vacuum. Without further purification, this resulted in the product in 66% yield (5.67 g) as a yellow oil. The analytical data are identical to the reported data.^40^

#### Synthesis of 1,1,2,2-Tetramethyl-1,2-bis(2-phenyl-1,3-dithian-2-yl)disilane (**5a**)

2-Phenyl-1,3-dithiane (11.59 g, 59.05 mmol) was dissolved in tetrahydrofuran and cooled to 0 °C. After the slow addition of 36.91 mL of 1.6 M *n*-butyllithium (59.05 mmol), the reaction mixture was stirred at room temperature for 30 min. Subsequently, it was added to a solution of 5.00 mL of 1,2-dichloro-1,1,2,2-tetramethyldisilane (26.84 mmol) in tetrahydrofuran at 0 °C, warmed to room temperature, and stirred for another 5 min. The reaction mixture was further aqueously worked up with a saturated NH_4_Cl solution. The organic layer was separated from the aqueous layer. Then, the aqueous layer was extracted 3 times with dichloromethane. After drying the combined organic phases over Na_2_SO_4_, the volatile components were removed under vacuum. Recrystallization from acetone at −30 °C gave the product in 82% yield (11.2 g) as a white solid. Data for **5a** are as follows. *M*_p_: 267–270 °C. Elem. Anal. calcd for C_24_H_34_S_4_Si_2_: C, 56.86%; H, 6.76%; S, 25.30%. Found C, 56.51%; H, 6.38%; S, 25.23%. ^1^H NMR (300 MHz, CDCl_3_) δ 7.88 (d, *J* = 8.0 Hz, 4H, Aryl-*H*), 7.35 (t, *J* = 7.7 Hz, 4H, Aryl-*H*), 7.16 (t, *J* = 7.3 Hz, 2H, Aryl-*H*), 2.69 (t, *J* = 13.2 Hz, 4H, −S–C*H*_2_–CH_2_−), 2.37 (d, *J* = 14.0 Hz, 4H, −S–C*H*_2_–CH_2_−), 1.93 (m, 4H, −S–CH_2_–C*H*_2_−), 0.24 (s, 12H, −C*H*_3_). ^13^C NMR (75 MHz, CDCl_3_) δ 140.69, 129.90, 128.27, 125.31 (Aryl-*C*), 48.12 (−S*–C*–S−), 25.53 (−S–*C*H_2_–CH_2_−), 25.11 (−S–CH_2_–*C*H_2_−), −3.00 (−Si–(*C*H_3_)_2_). ^29^Si NMR (60 MHz, C_6_D_6_) δ −5.50 (−*Si*–Me_2_). HRMS: (LIFDI^+^) calcd for [C_14_H_23_S_2_Si_2_]^+^ (M–C_10_H_11_S_2_^•^): 311.0780. Found: 311.1008.

#### Synthesis of 1,1,2,2-Tetraethyl-1,2-bis-(2-phenyl-1,3-dithian-2-yl)disilane (**5b**)

2-Phenyl-1,3-dithiane (4.50 g, 22.92 mmol) was dissolved in tetrahydrofuran and cooled to 0 °C. After the slow addition of 2.08 mL of 11 M *n*-butyllithium (22.92 mmol), the reaction mixture was stirred at room temperature for 30 min. Subsequently, it was added to a solution of 2.55 g of 1,2-dichloro-1,1,2,2-tetraethyldisilane (10.48 mmol) in tetrahydrofuran at 0 °C, warmed to room temperature, and stirred for another 5 min. The reaction mixture was further aqueously worked up with a saturated NH_4_Cl solution. The organic layer was separated from the aqueous layer. Then, the aqueous layer was extracted 3 times with dichloromethane. After drying the combined organic phases over Na_2_SO_4_, the volatile components were removed under vacuum. Recrystallization from acetone at −30 °C gave the product in 77% yield (4.56 g) as a white solid. Data for **5b** are as follows. *M*_p_: 207–210 °C. Elem. Anal. calcd for C_28_H_42_S_4_Si_2_: C, 59.73%; H, 7.52%; S, 22.78%. Found: C, 59.44%; H, 7.74%; S 22.59%. ^1^H NMR (300 MHz, CDCl_3_) δ 8.01 (d, *J* = 7.5 Hz, 4H, Aryl-*H*), 7.36 (t, *J* = 7.7 Hz, 4H, Aryl-*H*), 7.17 (t, *J* = 7.2 Hz, 2H, Aryl-*H*), 2.73 (t, *J* = 12.2 Hz, 4H, −S–CH_2_–C*H*_2_−), 2.36 (d, *J* = 13.9 Hz, 4H, −S–C*H*_2_–CH_2_−), 2.00 (q, *J* = 12.9 Hz, 2H, −S–C*H*_2_–CH_2_−), 1.83 (d, *J* = 13.5 Hz, 2H, −S–C*H*_2_–CH_2_−), 1.07–0.93 (m, 8H, −Si–C*H*_2_−), 0.89 (t, *J* = 7.4 Hz, 12H, −Si–CH_2_–C*H*_3_). ^13^C NMR (76 MHz, CDCl_3_) δ 141.42, 130.15, 128.42, 125.36 (Aryl*-C*), 49.60 (-S-*C*-S-), 25.62 (−S–*C*H_2_−), 25.02 (−S–CH_2_–*C*H_2_-), 9.25 (−*C*H_3_), 5.34 (−*C*H_2_−). ^29^Si NMR (40 MHz, CDCl_3_) δ −0.44 (−*Si*–Et_2_). HRMS: (LIFDI^+^) calcd for [C_18_H_31_S_2_Si_2_]^+^ (M–C_10_H_11_S_2_^•^): 367.1406. Found: 367.1667.

#### Synthesis of 2,2,3,3-Tetramethyl-1,1,4,4-tetraphenyl-1,4-bis(2-phenyl-1,3-dithian-2-yl)tetrasilane (**5c**)

2-Phenyl-1,3-dithiane (1.56 g, 7.97 mmol) was dissolved in tetrahydrofuran and cooled to 0 °C. After the slow addition of 4.98 mL of 1.6 M *n*-butyllithium (7.97 mmol), the reaction mixture was stirred at 0 °C for 2 h. Subsequently, it was added to a solution of 2.00 g of 1,4-dichloro-2,2,3,3-tetramethyl-1,1,4,4-tetraphenyltetrasilane (3.62 mmol) in tetrahydrofuran at 0 °C and stirred for another 2 h at 0 °C. The reaction mixture was further aqueously worked up with a saturated NH_4_Cl solution. The organic layer was separated from the aqueous layer. Then, the aqueous layer was extracted 3 times with diethyl ether. After drying the combined organic phases over Na_2_SO_4_, the volatile components were removed under vacuum. Flash column chromatography (with *n*-heptane/ethyl acetate 20:1) and subsequent recrystallization from acetone at −30 °C gave the product in 76% yield (2.40 g) as a white solid. Data for **5c** are as follows. *M*_p_: 200–203 °C. Elem. Anal. calcd for C_48_H_54_S_4_Si_4_: C, 66.15%; H, 6.25%; S, 14.71%. Found C, 66.18%; H, 6.19%; S, 14.39%. ^1^H NMR (300 MHz, C_6_D_6_) δ 7.78 (dd, *J* = 6.4, 2.9 Hz, 8H, Aryl-*H*), 7.69 (d, *J* = 7.5 Hz, 4H, Aryl-*H*), 7.15 (d, *J* = 3.8 Hz, 12H, Aryl-*H*), 6.99 (t, *J* = 7.7 Hz, 4H, Aryl-*H*), 6.85 (t, *J* = 7.2 Hz, 2H, Aryl-*H*), 2.39 (t, *J* = 12.5 Hz, 4H, −S–CH_2_–C*H*_2_−), 1.86 (d, *J* = 14.0 Hz, 4H, −S–C*H*_2_–CH_2_−), 1.71 (q, *J* = 13.0 Hz, 2H, S–C*H*_2_–CH_2_), 1.15 (d, *J* = 13.4 Hz, 2H, −S–C*H*_2_–CH_2_−), 0.61 (s, 12H, C*H*_3_). ^13^C NMR (76 MHz, C_6_D_6_) δ 140.40, 138.11, 133.78, 131.05, 129.68, 127.95, 127.55, 125.65 (Aryl-*C*), 50.36 (−S–*C*–S−), 25.62 (−S–*C*H_2_–CH_2_−), 24.94 (−S–CH_2_–*C*H_2_−), −0.28 (−Si-(*C*H_3_)_2_). ^29^Si NMR (60 MHz, C_6_D_6_) δ −11.10 (−*Si*–Me_2_), −38.75 (−*Si*–Ph_2_). HRMS: (LIFDI^+^) calcd for [C_38_H_43_S_2_Si_4_]^+^ (M–C_10_H_11_S_2_^•^): 675.1883. Found: 676.4990.

#### Synthesis of (1,1,2,2-Tetraethyldisilane-1,2-diyl)bis(phenylmethanone) (**6a**)

1,1,2,2-Tetraethyl-1,2-bis(2-phenyl-1,3-dithian-2-yl)disilane (2.56 g, 4.55 mmol) was dissolved in dichloromethane. A small amount of dry methanol was added and then cooled to 0 °C. After the slow addition of 5.86 g of (diacetoxyiodo)benzene (18.19 mmol) and 2.24 mL of boron trifluoride diethyl etherate (18.19 mmol), stirring at room temperature was continued overnight. Subsequently, *n*-pentane was added, and to remove the BF_3_·OEt_2_, the mixture was filtered through silica gel. The reaction mixture was further aqueously worked up with a saturated NH_4_Cl solution. The organic layer was separated from the aqueous layer. Then, the aqueous layer was extracted 3 times with diethyl ether. After drying the combined organic phases over Na_2_SO_4_, the volatile components were removed under vacuum. Gradual flash column chromatography (starting with *n*-heptane/toluene 5:1) and subsequent recrystallization from *n*-pentane at −70 °C gave the product in 22% yield (0.40 g) as a yellow solid. Data for **6a** are as follows. *M*_p_: 54–56 °C. Elem. Anal. calcd for C_22_H_30_O_2_Si_2_: C, 69.06%; H, 7.90%. Found: C, 68.93%; H, 7.92%. ^1^H NMR (300 MHz, CDCl_3_) δ 7.70 (d, *J* = 7.2 Hz, 4H, Aryl-*H*), 7.50 (t, *J* = 7.1 Hz, 2H, Aryl-*H*), 7.40 (t, *J* = 7.5 Hz, 4H, Aryl-*H*), 1.17–1.01 (m, 8H, −Si–C*H*_2_−), 0.98 (t, *J* = 6.1 Hz, 12H, −Si–CH_2_–C*H*_3_). ^13^C NMR (76 MHz, CDCl_3_) δ 235.17 (*C* = O), 142.76, 132.98, 128.77, 127.35 (Aryl-*C*), 8.59 (−*C*H_3_), 4.67 (−*C*H_2_−). ^29^Si NMR (40 MHz, C_6_D_6_) δ −16.17 (−*Si*–Et_2_). IR (neat): ν(C = O) 1603, 1588, 1571 cm^–1^. UV–vis: λ [nm], ε[L mol^–1^ cm^–1^] 408, 341; 428, 465; 446, 343. HRMS: (LIFDI^+^) calcd for [C_22_H_30_O_2_Si_2_]^+•^ (M^+•^): 382.1784. Found: 382.1960.

#### Synthesis of (2,2,3,3-Tetramethyl-1,1,4,4-tetraphenyltetrasilane-1,4-diyl)bis(phenylmethanone) (**6b**)

2,2,3,3-Tetramethyl-1,1,4,4-tetraphenyl-1,4-bis(2-phenyl-1,3-dithian-2-yl)tetrasilane (1.00 g, 1.15 mmol) was dissolved in dichloromethane. A small amount of dry methanol was added and then cooled to 0 °C. After the slow addition of 1.48 g of (diacetoxyiodo)benzene (4.59 mmol) and 0.57 mL of boron trifluoride diethyl etherate (4.59 mmol), stirring at room temperature was continued overnight. Subsequently, *n*-pentane was added, and to remove the BF_3_·OEt_2_, the mixture was filtered through silica gel. The reaction mixture was further aqueously worked up with a saturated NH_4_Cl solution. The organic layer was separated from the aqueous layer. Then, the aqueous layer was extracted three times with diethyl ether. After drying the combined organic phases over Na_2_SO_4_, the volatile components were removed under vacuum. Gradual flash column chromatography (starting with *n*-heptane/toluene 4:1) and subsequent recrystallization from *n*-pentane at −70 °C gave the product in 13% yield (0.10 g) as a yellow solid. Data for **6b** are as follows. *M*_p_: 147–150 °C. Elem. Anal. calcd for C_42_H_42_O_2_Si_4_: C, 72.99%; H, 6.13%. Found: C, 73.24%; H, 6.04%. ^1^H NMR (300 MHz, CDCl_3_) δ 7.60 (d, *J* = 7.1 Hz, 4H, Aryl-*H*), 7.44 (d, *J* = 6.2 Hz, 8H, Aryl-*H*), 7.35–7.17 (m, 18H, Aryl-*H*), 0.01 (s, 12H, −Si–C*H*_3_). ^13^C NMR (76 MHz, CDCl_3_) δ 232.76 (*C* = O), 142.39, 136.15, 133.89, 132.91, 129.68, 128.52, 128.41, 128.38 (Aryl-*C*), −3.98 (−*C*H_3_). ^29^Si NMR (40 MHz, CDCl_3_) δ −24.46 (−*Si*–Me_2_), −40.64 (-*Si*-Ph_2_). IR (neat): ν(C = O) 1604, 1587, 1572 cm^–1^. UV–vis: λ [nm], ε[L mol^–1^ cm^–1^] 408, 933; 426, 1259; 446, 871. HRMS: (LIFDI^+^) calcd for [C_42_H_42_O_2_Si_4_]^+•^ (M^+•^): 690.2262. Found: 690.3032.

#### Synthesis of (*+/-*)-(((Fluorodimethylsilyl)(methoxy)(phenyl)methyl)dimethylsilyl)(phenyl)methanone (**6c**)

1,1,2,2-Tetramethyl-1,2-bis(2-phenyl-1,3-dithian-2-yl)disilane (1.00 g, 1.97 mmol) was dissolved in chloroform. A small amount of dry methanol was added and then cooled to 0 °C. After the slow addition of 2.54 g of (diacetoxyiodo)benzene (7.89 mmol) and 0.97 mL of boron trifluoride diethyl etherate (7.89 mmol), stirring at room temperature was continued overnight. Subsequently, *n*-pentane was added, and to remove the BF_3_·OEt_2_, the mixture was filtered through silica gel. The reaction mixture was further aqueously worked up with a saturated NH_4_Cl solution. The organic layer was separated from the aqueous layer. Then, the aqueous layer was extracted three times with diethyl ether. After drying the combined organic phases over Na_2_SO_4_, the volatile components were removed under vacuum. Gradual flash column chromatography (starting with *n*-heptane/toluene 4:1) and subsequent recrystallization from *n*-pentane at −70 °C gave the product in 83% yield (0.59 g) as a yellow solid. Data for **6c** are as follows. Mp: 77–79 °C. Elem. Anal. calcd for C_19_H_25_FO_2_Si_2_: C, 63.29%; H, 6.99%. Found C, 63.05%; H, 7.15%. ^1^H NMR (300 MHz, CDCl_3_) δ 7.85–7.80 (m, 2H, Aryl-*H*), 7.48 (d, *J* = 7.3 Hz, 1H, Aryl-*H*), 7.41 (t, *J* = 7.3 Hz, 2H, Aryl-*H*), 7.30 (t, *J* = 7.2 Hz, 2H, Aryl-*H*), 7.17 (d, *J* = 8.0 Hz, 3H, Aryl-*H*), 3.41 (s, 3H, −O–C*H*_3_), 0.45 (d, *J* = 7.6 Hz, 3H, −C*H*_3_), 0.34 (s, 3H), 0.28 (d, *J* = 7.9 Hz, 6H, −C*H*_3_). ^13^C NMR (76 MHz, CDCl_3_) δ 233.66 (*C* = O), 142.16, 139.85, 132.50, 128.47, 128.37, 128.34, 125.44 (Aryl-*C*), 56.42 (−O–*C*H_3_), 1.14 (−*C*H_3_), 0.95 (−*C*H_3_), 0.44 (−*C*H_3_), 0.26 (−*C*H_3_), −2.87 (−*C*H_3_), −3.68 (−*C*H_3_). ^29^Si NMR (40 MHz, CDCl_3_) δ 29.81 (d, *J* = 292 Hz, −*Si*–F), −6.49 (−*Si*–Me_2_). ^19^F NMR (188 MHz, C_6_D_6_) δ −107.70 (−Si–*F*). IR (neat): ν(C = O) 1609, 1587, 1572 cm^–1^. UV–vis: λ [nm], ε [L mol^–1^ cm^–1^] 403, 83; 422, 100; 446, 71. HRMS: (LIFDI^+^) calcd for [C_19_H_25_FO_2_Si_2_]^+•^ (M^+•^): 360.1377. Found: 360.1615.

#### Synthesis of Phenyl(1,1,2,2-tetramethyl-2-(2-phenyl-1,3-dithian-2-yl)disilaneyl)methanone (**6d**)

1,1,2,2-Tetramethyl-1,2-bis(2-phenyl-1,3-dithian-2-yl)disilane (1.00 g, 1.97 mmol) was dissolved in chloroform. A small amount of dry methanol was added and then cooled to 0 °C. After the slow addition of 2.54 g of (diacetoxyiodo)benzene (7.89 mmol), stirring at room temperature was continued overnight. The reaction mixture was further aqueously worked up with a saturated NH_4_Cl solution. The organic layer was separated from the aqueous layer. Then, the aqueous layer was extracted three times with diethyl ether. After drying the combined organic phases over Na_2_SO_4_, the volatile components were removed under vacuum. Gradual flash column chromatography (starting with *n*-heptane/ethyl acetate 20:1) and subsequent recrystallization from *n*-pentane at −70 °C gave the product in 34% yield (0.28 g) as a yellow solid. Data for **6d** are as follows. Mp: 77–81 °C. Elem. Anal. calcd for C_21_H_28_OS_2_Si_2_: C, 60.52%; H, 6.77%; S, 15.39%. Found: C, 60.25%; H, 6.75%; S, 15.70%. ^1^H NMR (300 MHz, C_6_D_6_) δ 8.02 (dd, *J* = 6.5, 3.1 Hz, 2H, Aryl-*H*), 7.97 (d, *J* = 7.5 Hz, 2H, Aryl-*H*), 7.21 (t, *J* = 8.0 Hz, 3H, Aryl-*H*), 7.13 (t, *J* = 2.8 Hz, 2H, Aryl-*H*), 6.99 (t, *J* = 7.3 Hz, 1H, Aryl-*H*), 2.39 (t, *J* = 12.4 Hz, 2H, −S–CH_2_–C*H*_2_−), 1.87 (d, *J* = 13.5 Hz, 2H, −S–C*H*_2_–CH_2_−), 1.75–1.58 (m, *J* = 26.3, 13.0 Hz, 1H, −S–C*H*_2_–CH_2_−), 1.18 (d, *J* = 13.6 Hz, 1H, −S–C*H*_2_–CH_2_−), 0.73 (s, 6H, −Si–C*H*_3_), 0.23 (s, 6H, −Si–C*H*_3_). ^13^C NMR (76 MHz, C_6_D_6_) δ 234.61 (*C*=O), 142.74, 140.94, 132.47, 129.73, 128.78, 128.69, 128.18, 125.86 (Aryl-*C*), 47.79 (−S–*C*–S−), 25.40 (−S–*C*H_2_−), 25.01 (−S–CH_2_–*C*H_2_−), −1.29 (−*C*H_3_), −4.86 (−*C*H_3_). ^29^Si NMR (40 MHz, C_6_D_6_) δ −5.06 (−*Si*–C−), −23.01 (−*Si*-(CO)-). IR (neat): ν(C=O) 1607, 1588, 1571 cm^–1^. UV–vis: λ [nm], ε[L mol^–1^ cm^–1^] 405, 124; 423, 152; 444, 105. HRMS: (LIFDI^+^) calcd for [C_21_H_28_OS_2_Si_2_]^+•^ (M^+•^): 416.1120. Found: 416.1268.

## Supporting Information Available

Merged_cifs (CIF)
^1^H-, ^13^C-, and ^29^Si-NMR spectra for all isolated compounds and X-ray data of all structurally characterized compounds (PDF)

## Supplementary Material

ao2c05258_si_001.cif
