# Supplementary material for: Synthesis and Characterization of a Variety of α,ω-Bisacylpolysilanes—A Study on Reactivity and Accessibility
Source: ACS Omega. 2022 Oct 13;7(42):38025–36. doi: 10.1021/acsomega.2c05258 (PMC9609067; doi:10.1021/acsomega.2c05258)
Supplement: Supplementary file 2 — ao2c05258_si_002.pdf [file ao2c05258_si_002.pdf]

Supporting Information

Synthesis and Characterization of a Variety of α,ω–Bisacylpolysilanes – A Study on Reactivity and Accessibility

Tanja Wiesner, Madeleine Heurix, Roland C. Fischer, Ana Torvisco, Michael Haas*

Institute of Inorganic Chemistry, Graz University of Technology, Stremayrgasse 9/IV, 8010 Graz (Austria)

Table of Contents

1. NMR–Spectroscopy 3

1.1. 1,2–dibenzyl–1,1,2,2–tetramethyldisilane (2a) 3
1.2. 1,2–dibenzyl–1,1,2,2–tetraphenyldisilane (2b) 4
1.3. 1,2–bis(dibromo(phenyl)methyl)–1,1,2,2–tetramethyldisilane (3a) 7
1.4. 1,2–bis(dibromo(phenyl)methyl)–1,1,2,2–tetraphenyldisilane (3b) 8
1.5. ((oxybis(bromo(phenyl)methylene))bis(1,1,2,2–tetramethyldisilane–2,1–diyl))bis(phenylmethanone) (4b) 10
1.6. (1,1,2,2–tetraphenyldisilane–1,2–diyl)bis(phenylmethanone) (4c) 12
1.7. 1,1,2,2–tetramethyl–1,2–bis(2–phenyl–1,3–dithian–2–yl)disilane (5a) 13
1.8. 1,1,2,2–tetraethyl–1,2–bis(2–phenyl–1,3–dithian–2–yl)disilane (5b) 15
1.9. 2,2,3,3–tetramethyl–1,1,4,4–tetraphenyl–1,4–bis(2–phenyl–1,3–dithian–2–yl)tetrasilane (5c) 17
1.10. (1,1,2,2–tetraethyldisilane–1,2–diyl)bis(phenylmethanone) (6a) 18
1.11. (2,2,3,3–tetramethyl–1,1,4,4–tetraphenyltetrasilane–1,4–diyl)bis(phenylmethanone) (6b) 20
1.12. (((fluorodimethylsilyl)(methoxy)(phenyl)methyl)dimethylsilyl)(phenyl)methanone (6c) 21
1.13. phenyl(1,1,2,2–tetramethyl–2–(2–phenyl–1,3–dithian–2–yl)disilaneyl)methanone (6d) 23

2. X–ray Crystallography 25

3. Photolysis Experiments 26

3.1. NMR–Data of the Photolysis of Compound 3b 26
3.1.1. NMR–Data of the Photolysis of Compound 3c 28

1. NMR–Spectroscopy

1.1. 1,2–dibenzyl–1,1,2,2–tetramethyldisilane (2a)

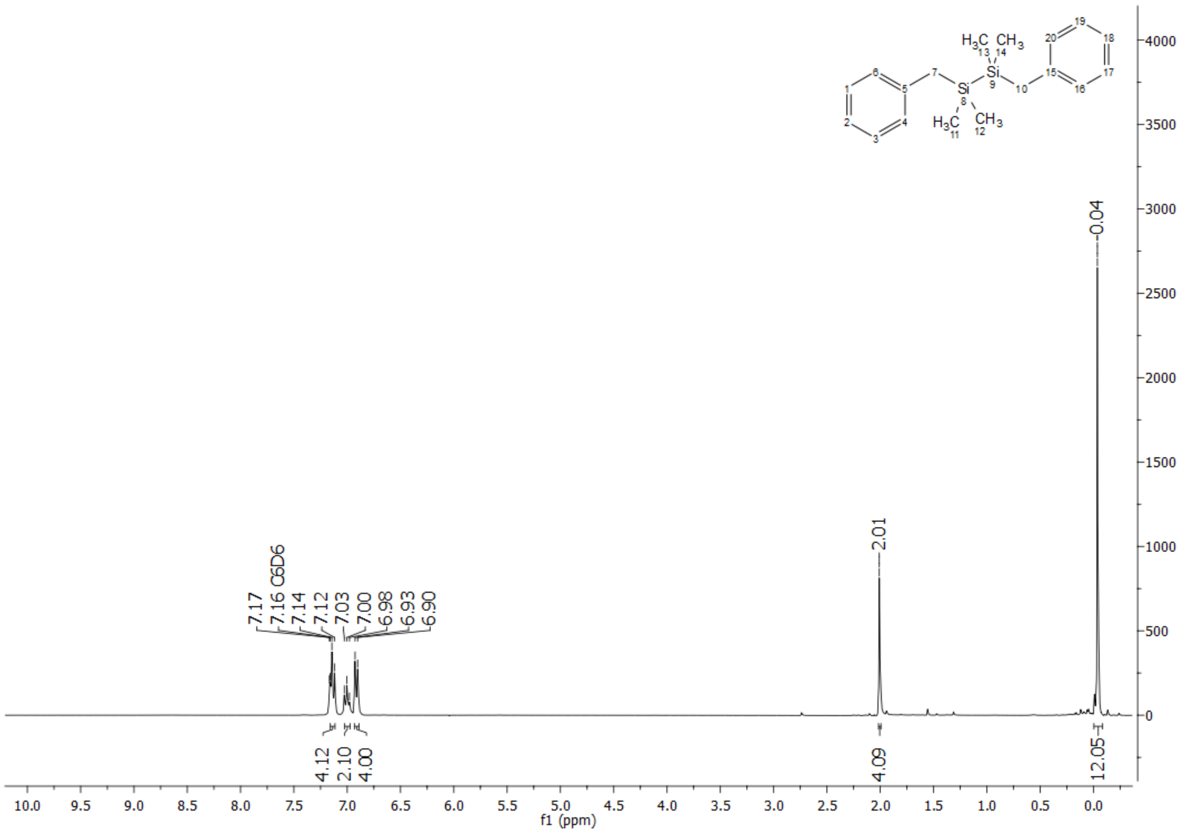

Figure S1: ^{1}H–NMR spectra (C_6D_6) of 1,2–dibenzyl–1,1,2,2–tetramethyldisilane (**2a**)

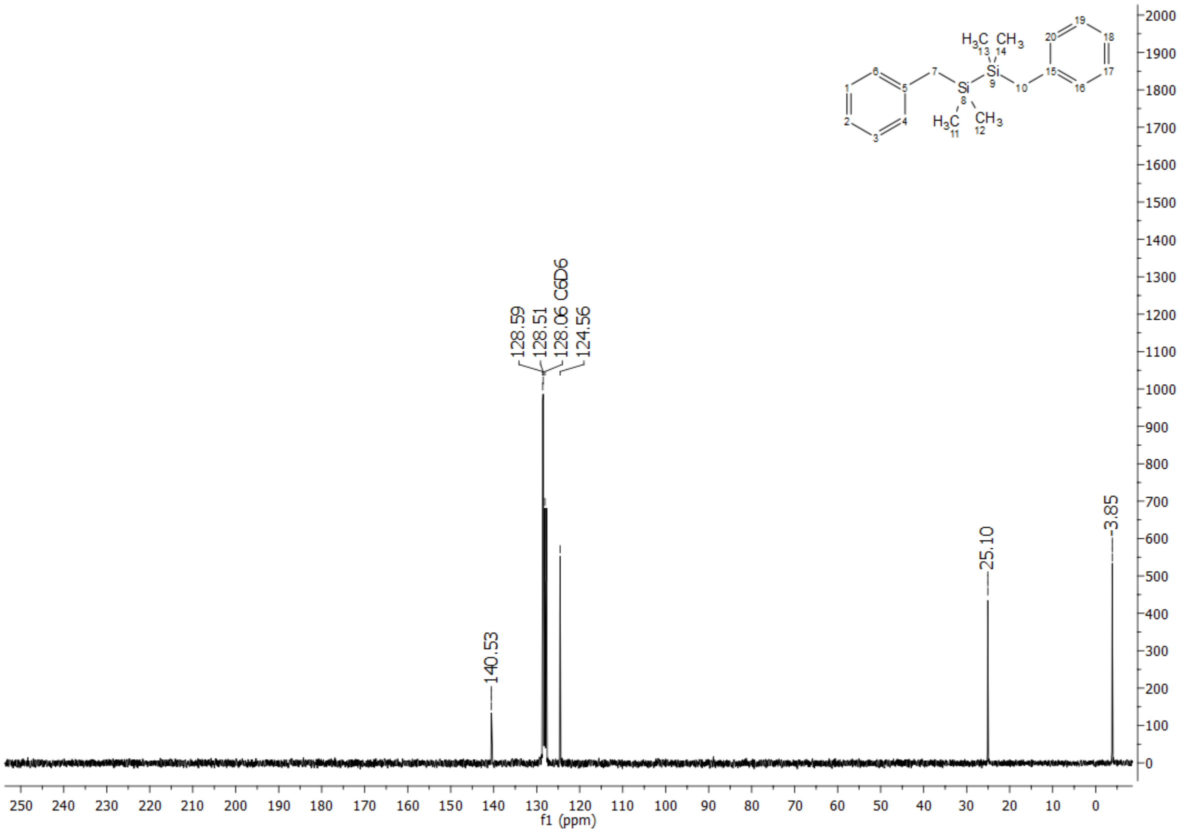

Figure S2: ^{13}C–NMR spectra (C_6D_6) of 1,2–dibenzyl–1,1,2,2–tetramethyldisilane (**2a**)

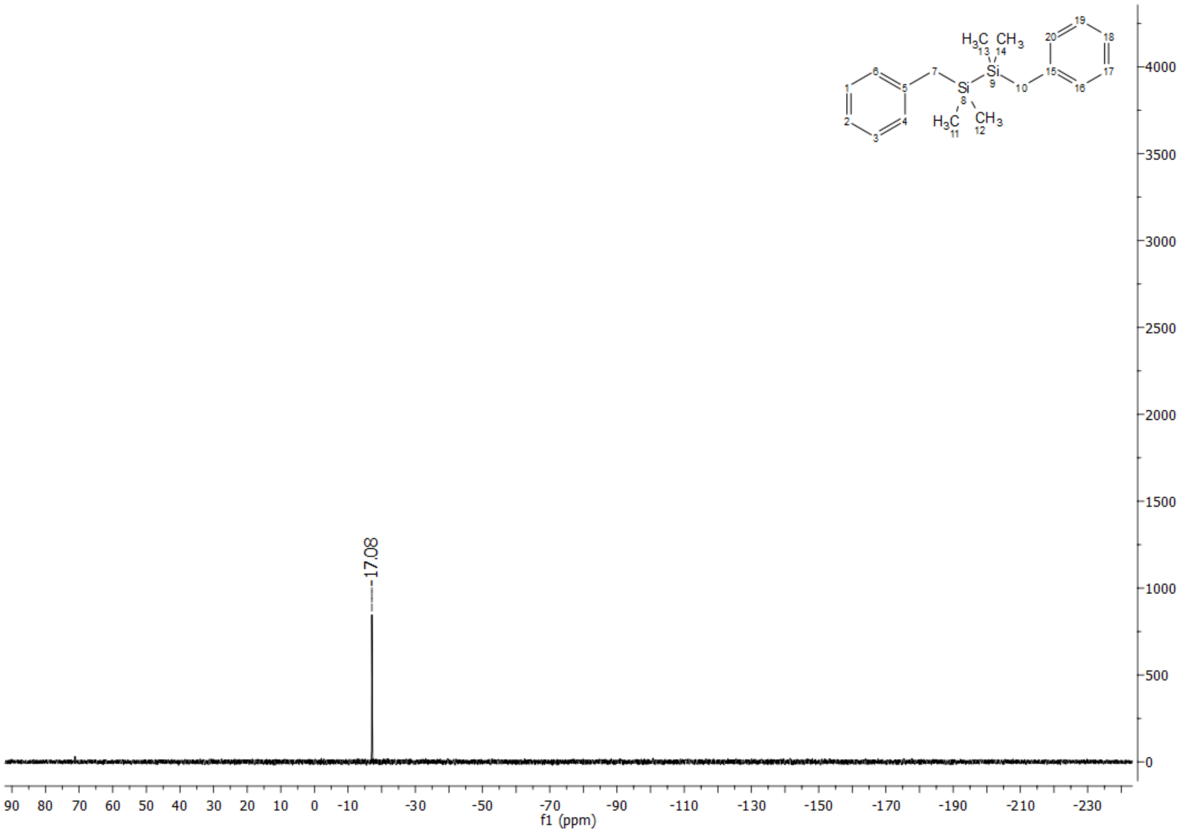

Figure S3: ^{29}Si–NMR spectra (C_6D_6) of 1,2–dibenzyl–1,1,2,2–tetramethyldisilane (**2a**)

1.2. 1,2–dibenzyl–1,1,2,2–tetraphenyldisilane (2b)

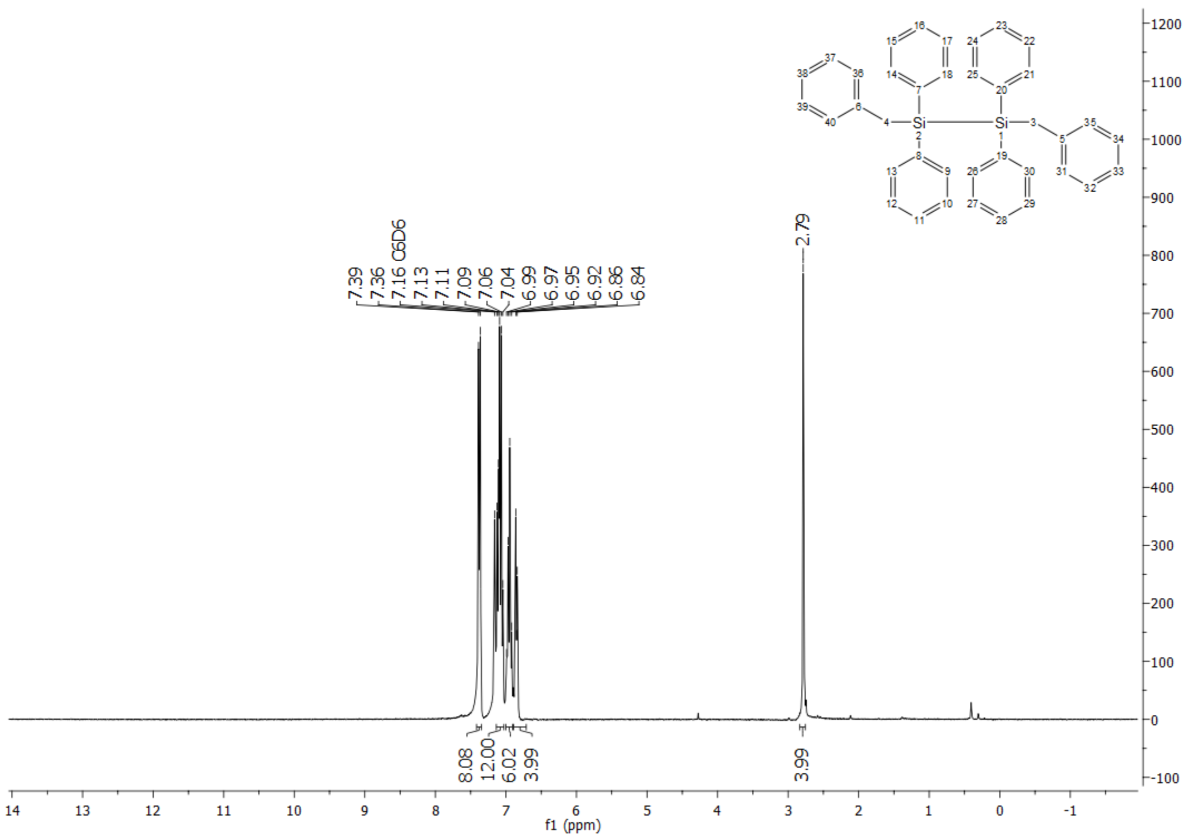

Figure S4: ^{1}H−NMR spectra (C_6D_6) of 1,2−dibenzyl−1,1,2,2−tetraphenyldisilane (**2b**)

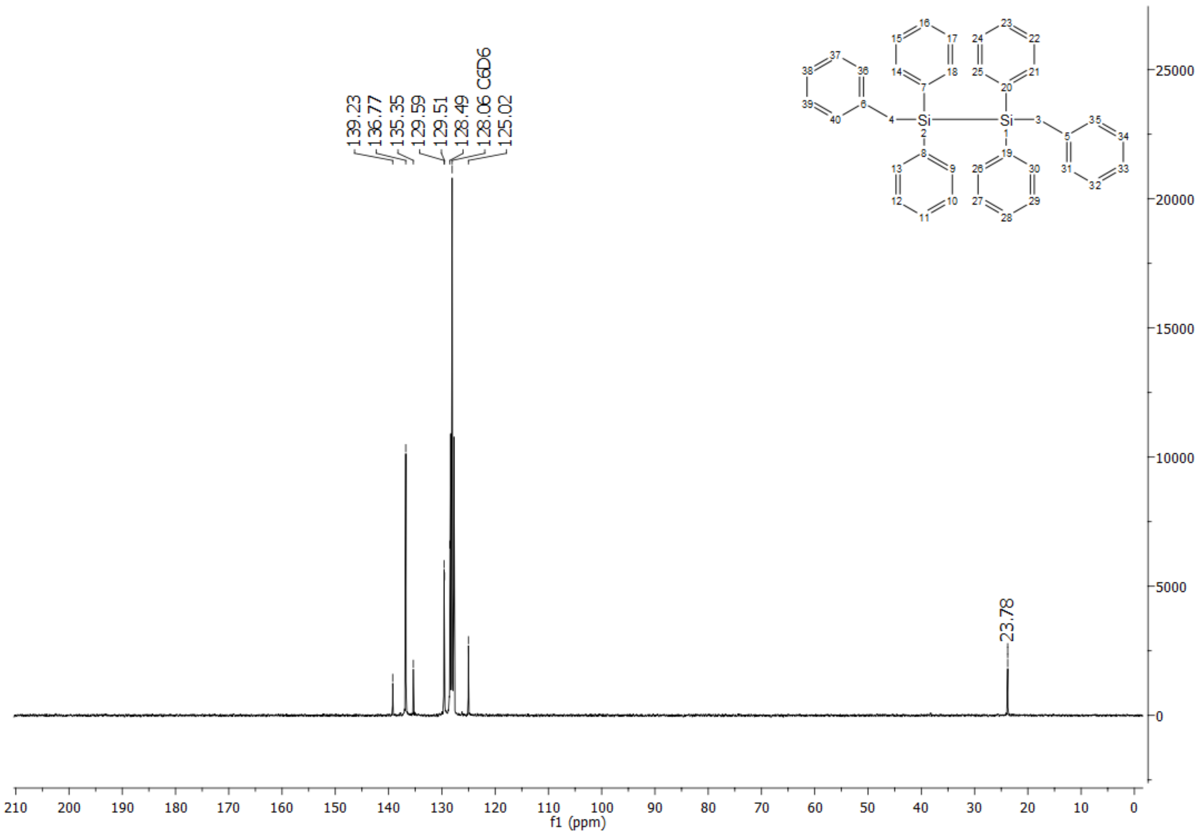

Figure S5: ^{13}C−NMR spectra (C_6D_6) of 1,2−dibenzyl−1,1,2,2−tetraphenyldisilane (**2b**)

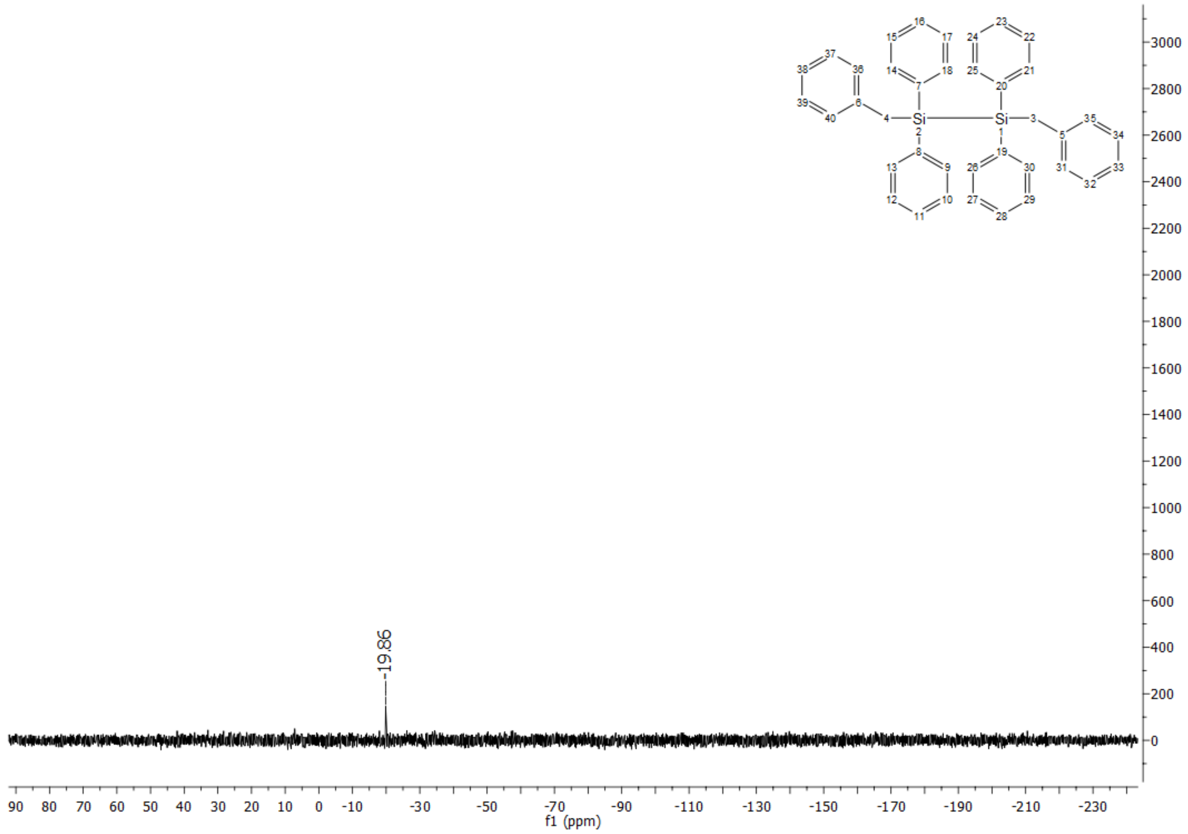

Figure S6: ^{29}Si−NMR spectra (C_6D_6) of 1,2−dibenzyl−1,1,2,2−tetraphenyldisilane (**2b**)

1.3. 1,2−bis(dibromo(phenyl)methyl)−1,1,2,2−tetramethyldisilane (3a)

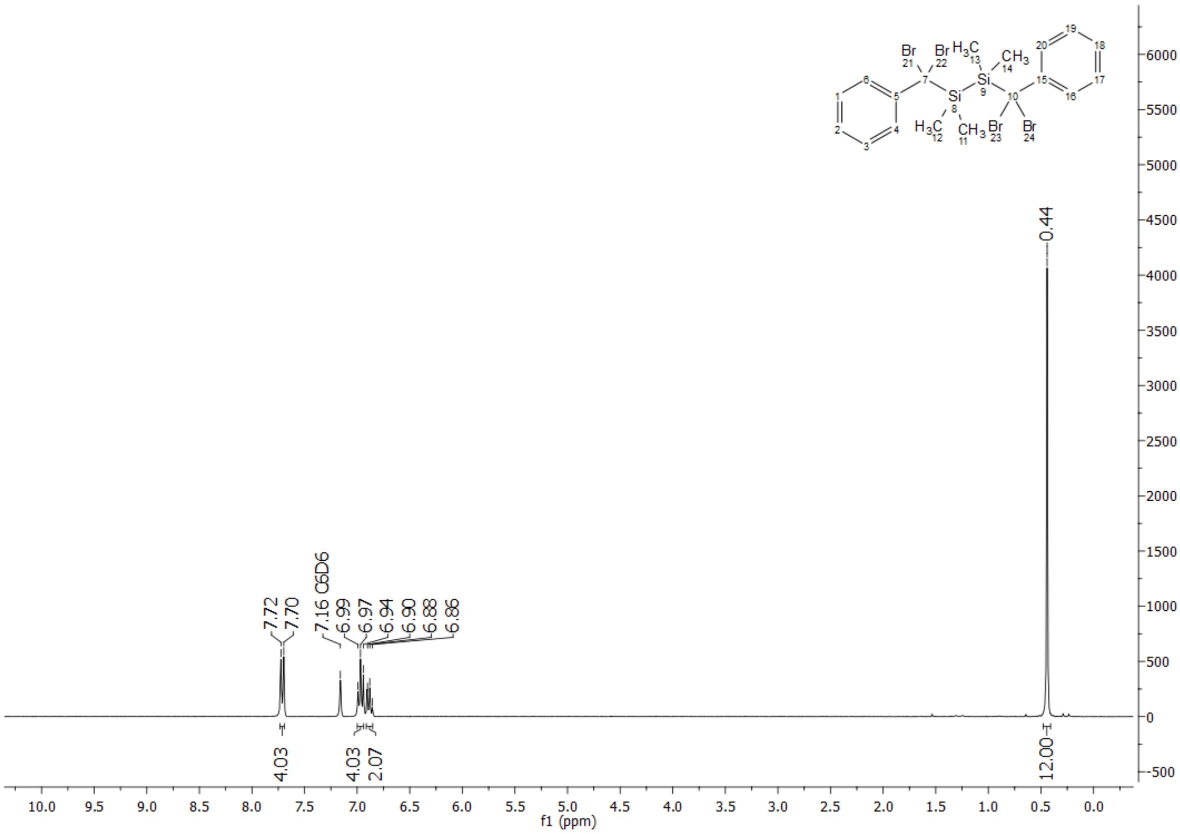

Figure S7: ^{1}H−NMR spectra (C_6D_6) of 1,2−bis(dibromo(phenyl)methyl)−1,1,2,2−tetramethyldisilane (**3a**)

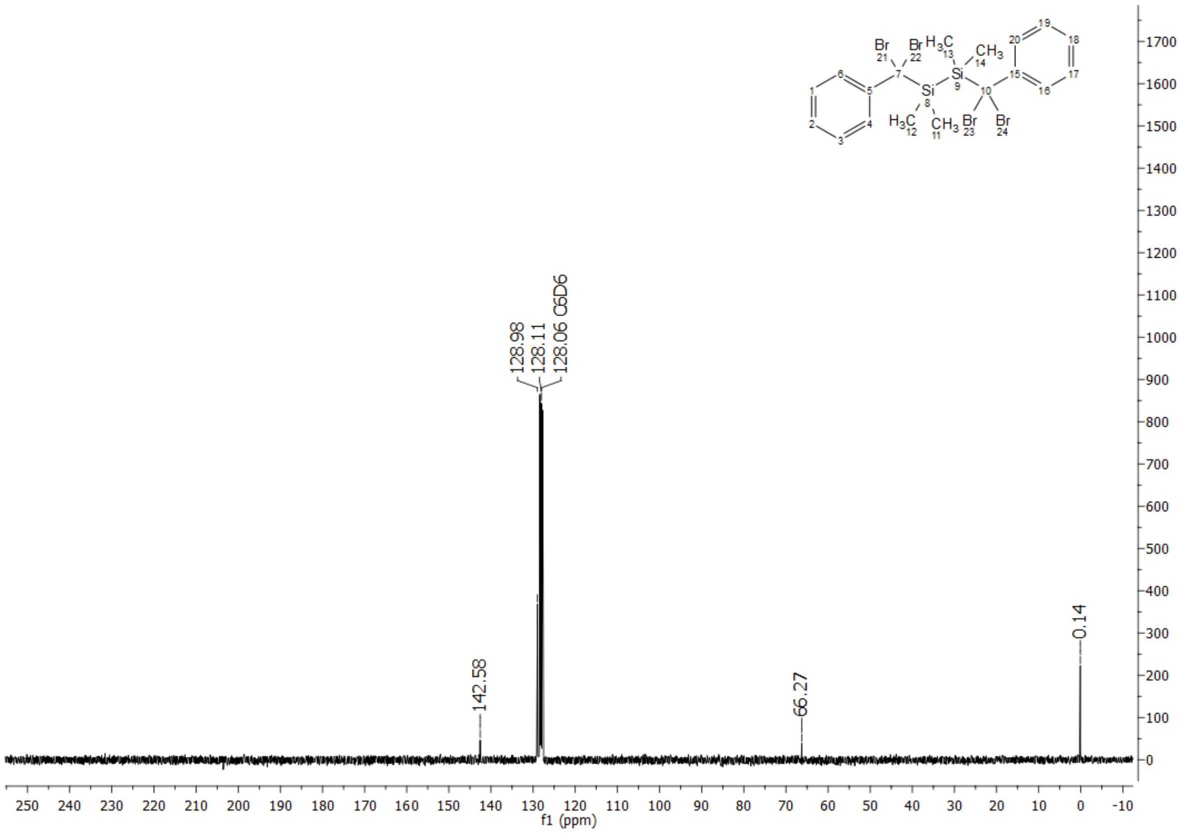

Figure S8: ^{13}C−NMR spectra (C_6D_6) of 1,2−bis(dibromo(phenyl)methyl)−1,1,2,2−tetramethyldisilane (**3a**)

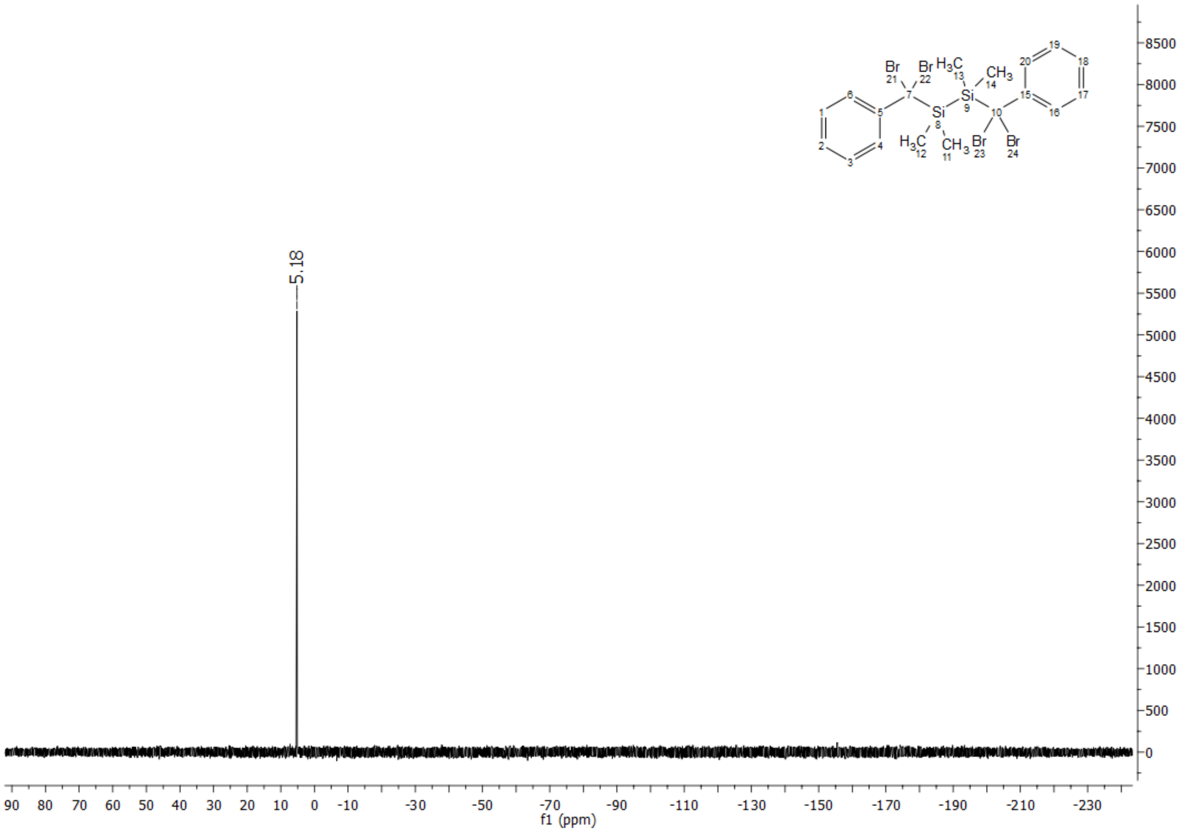

Figure S9: ^{29}Si−NMR spectra (C_6D_6) of 1,2−bis(dibromo(phenyl)methyl)−1,1,2,2−tetramethyldisilane (**3a**)

1.4. 1,2−bis(dibromo(phenyl)methyl)−1,1,2,2−tetraphenyldisilane (3b)

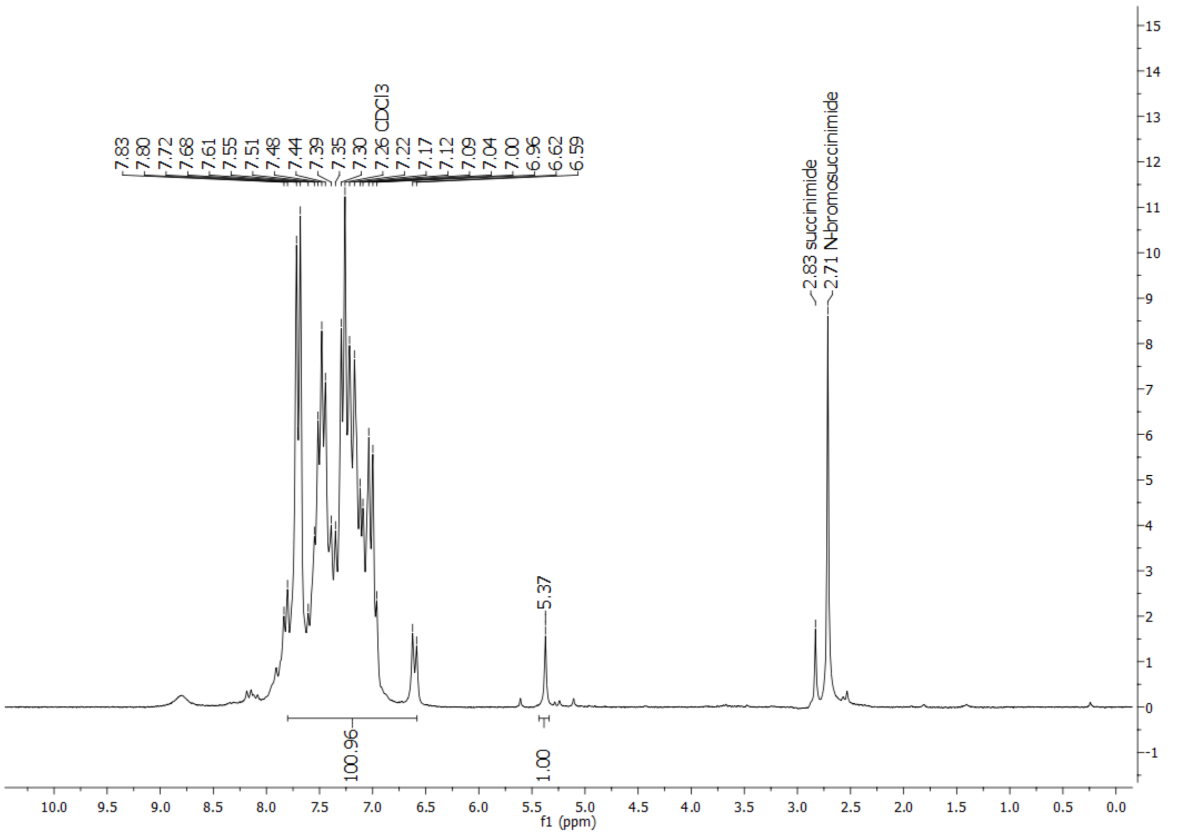

Figure S10: ^{1}H−NMR reaction spectra ($CDCl_3$) of **3b**

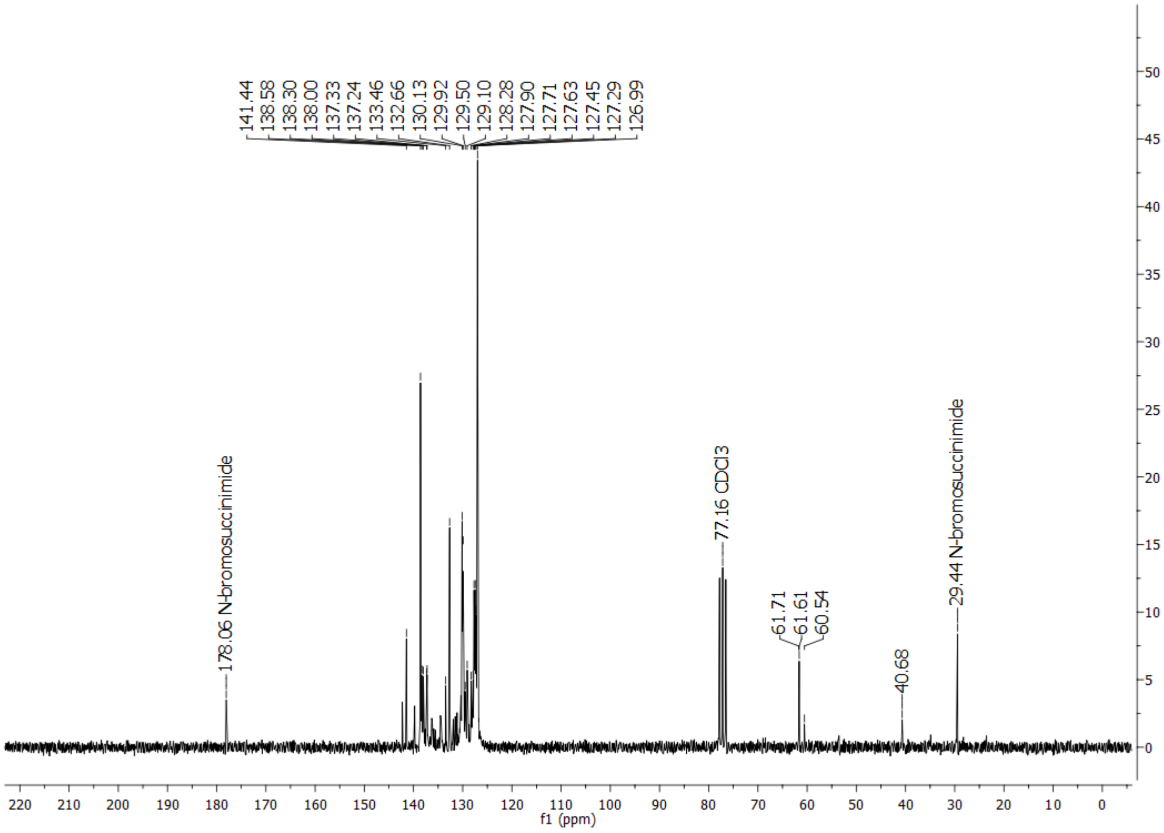

Figure S11: ^{13}C−NMR reaction spectra ($CDCl_3$) of **3b**

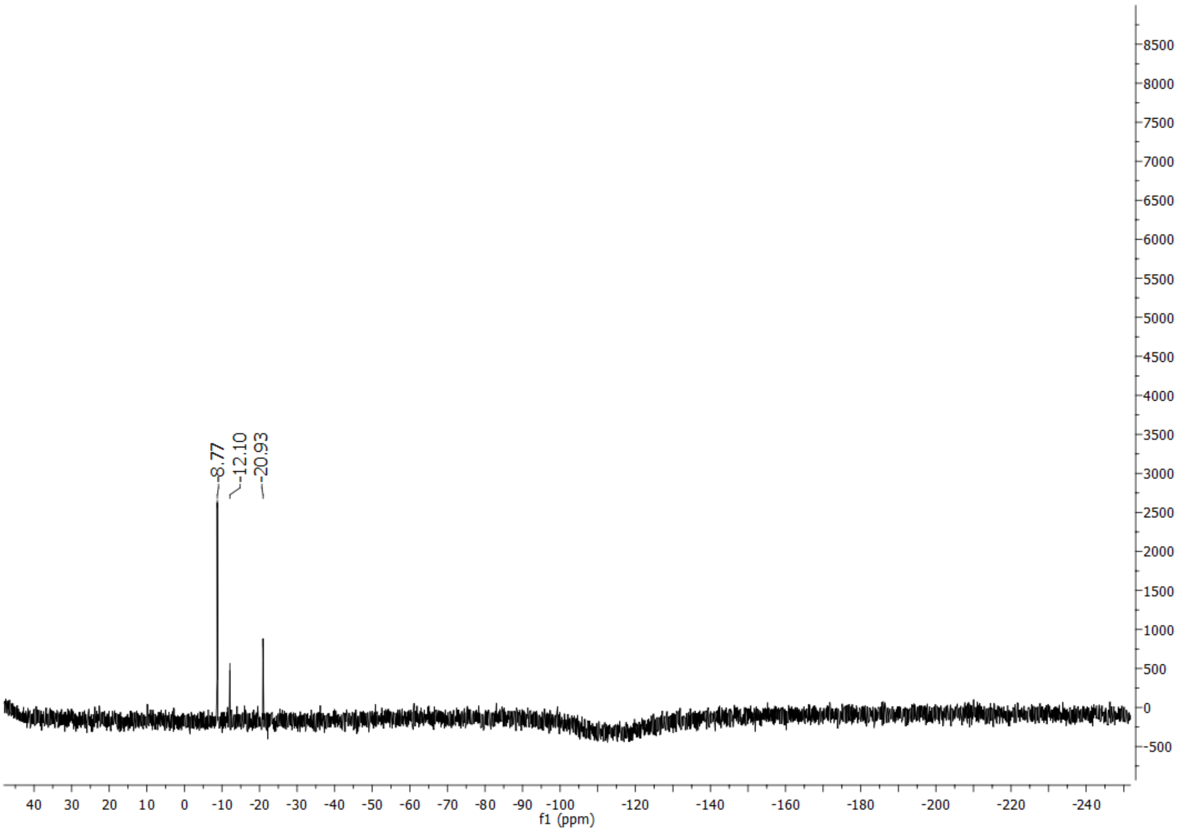

Figure S12: ^{29}Si−NMR reaction spectra ($CDCl_3$) of **3b**

1.5. ((oxybis(bromo(phenyl)methylene))bis(1,1,2,2–tetramethyldisilane–2,1–diyl))bis(phenylmethanone) (4b)

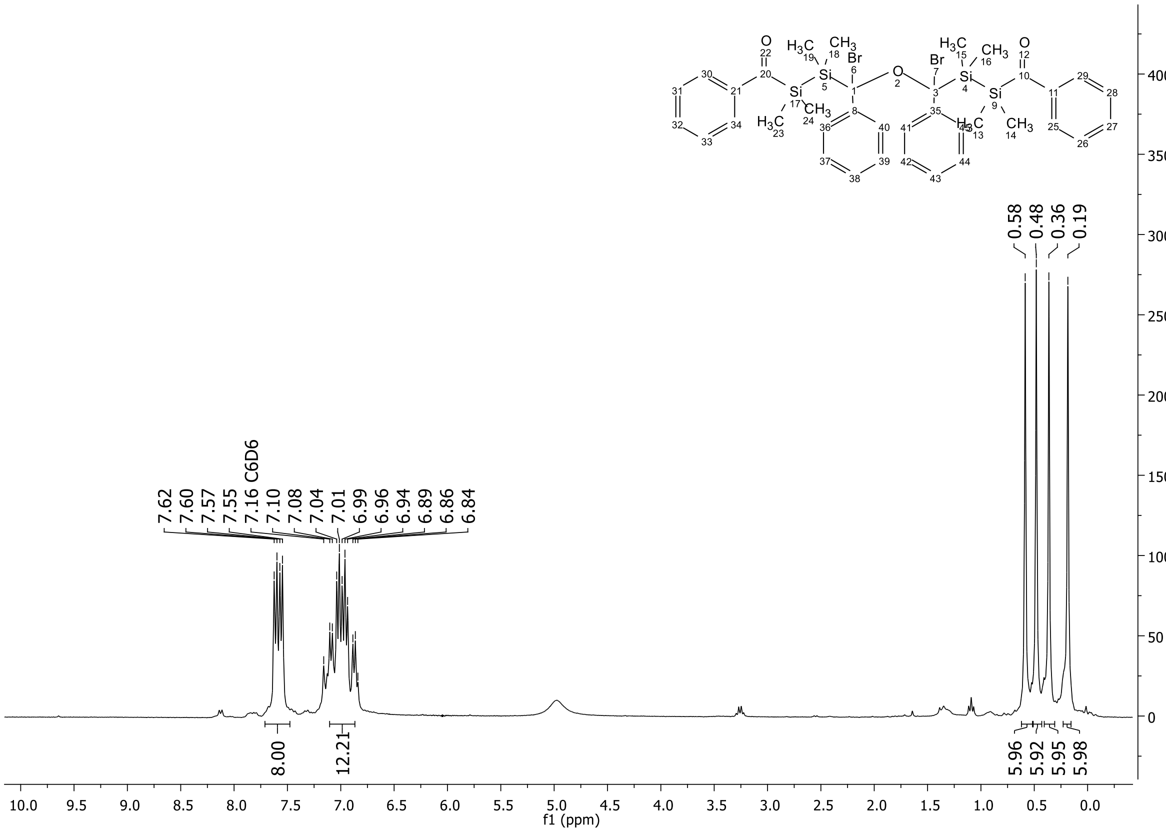

Figure S13: ^{1}H–NMR spectra (C_6D_6) of ((oxybis(bromo(phenyl)methylene))bis(1,1,2,2–tetramethyldisilane–2,1–diyl))bis(phenylmethanone) (**4b**)

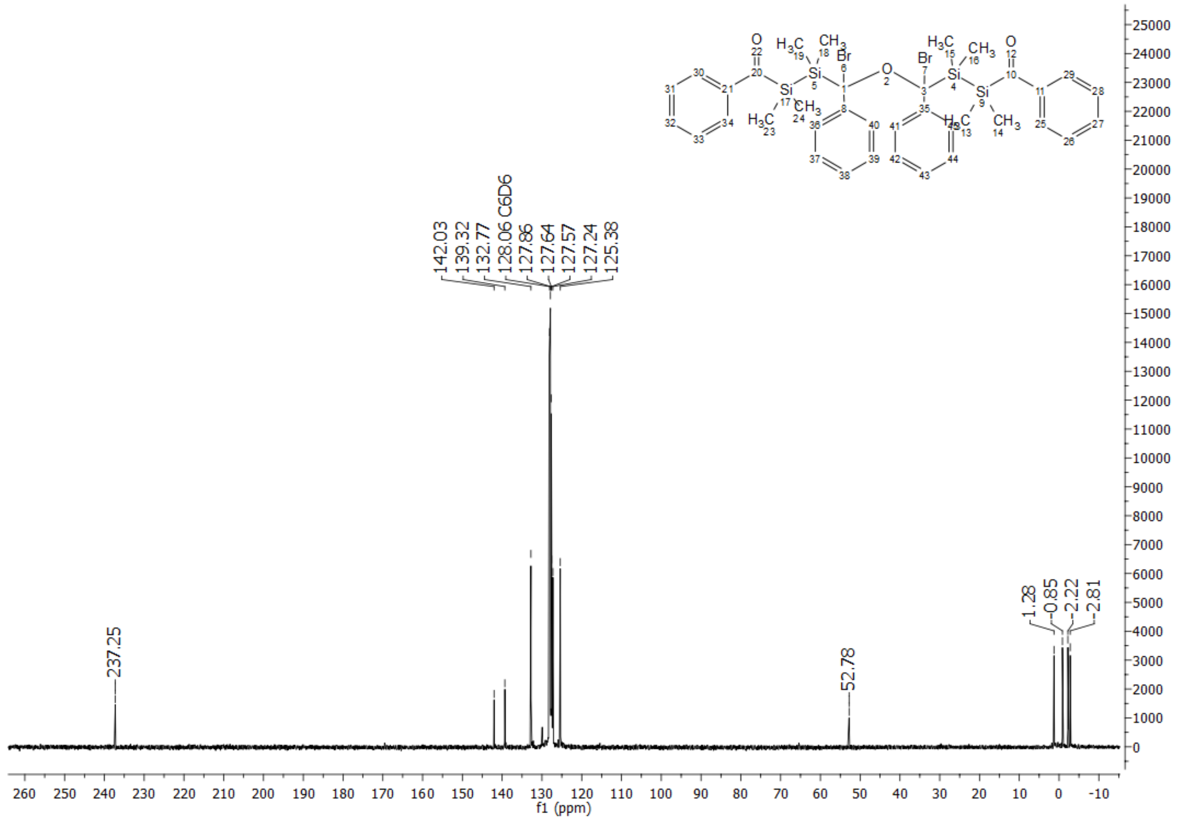

Figure S14: ^{13}C−NMR spectra (C_6D_6) of ((oxybis(bromo(phenyl)methylene))bis(1,1,2,2−tetramethyldisilane−2,1−diyl))bis(phenylmethanone) (**4b**)

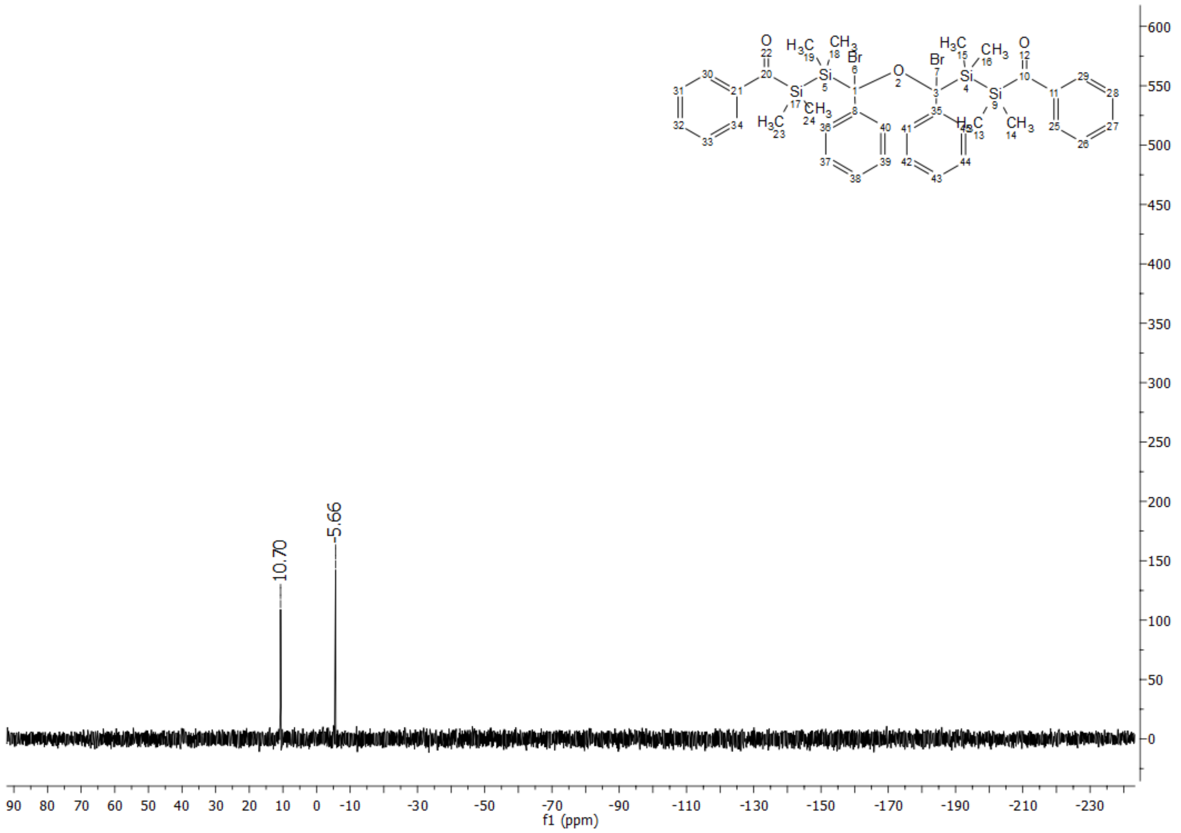

Figure S15: ^{29}Si−NMR spectra ($CDCl_3$) of ((oxybis(bromo(phenyl)methylene))bis(1,1,2,2−tetramethyldisilane−2,1−diyl))bis(phenylmethanone) (**4b**)

1.6. (1,1,2,2–tetraphenyldisilane–1,2–diyl)bis(phenylmethanone) (4c)

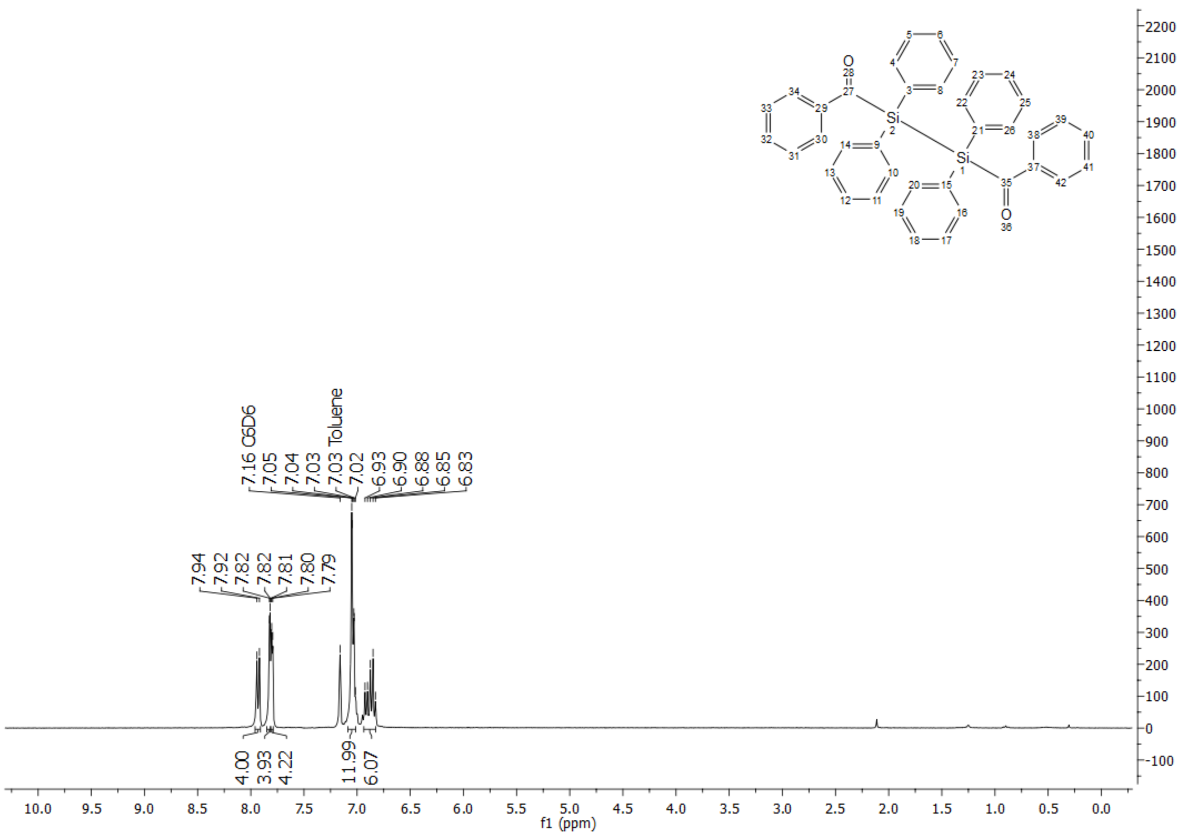

Figure S16: ^{1}H–NMR spectra (C_6D_6) of (1,1,2,2–tetraphenyldisilane–1,2–diyl)bis(phenylmethanone) (**4c**)

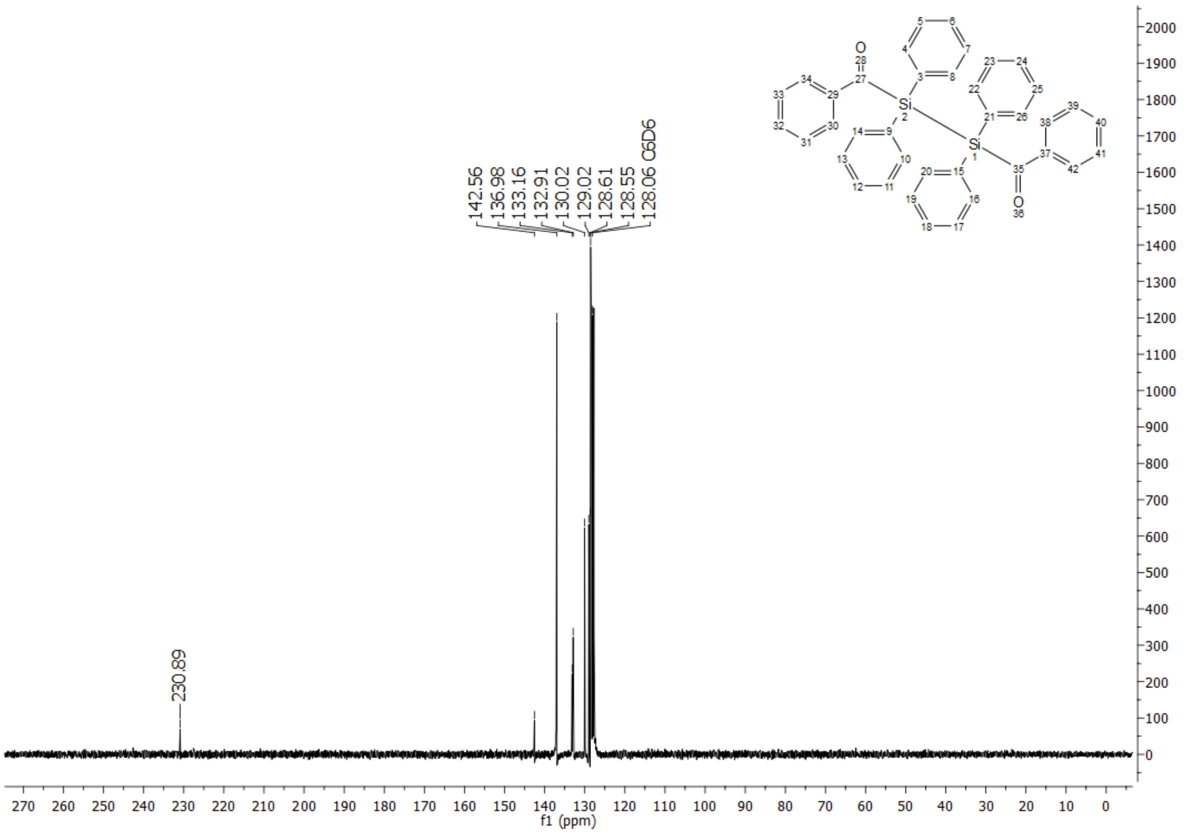

Figure S17: ^{13}C–NMR spectra (C_6D_6) of (1,1,2,2–tetraphenyldisilane–1,2–diyl)bis(phenylmethanone) (**4c**)

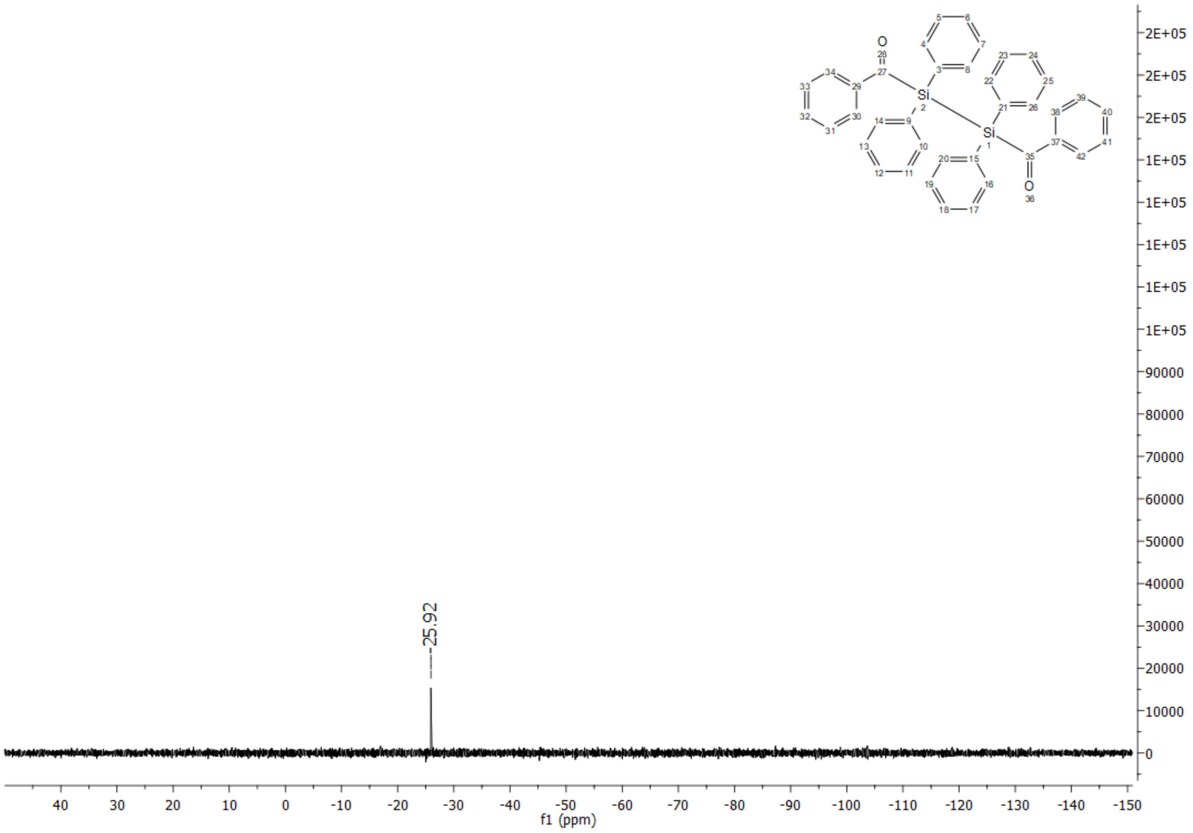

Figure S18: ^{29}Si–NMR spectra (C_6D_6) of (1,1,2,2–tetraphenyldisilane–1,2–diyl)bis(phenylmethanone) (**4c**)

1.7. 1,1,2,2–tetramethyl–1,2–bis(2–phenyl–1,3–dithian–2–yl)disilane (5a)

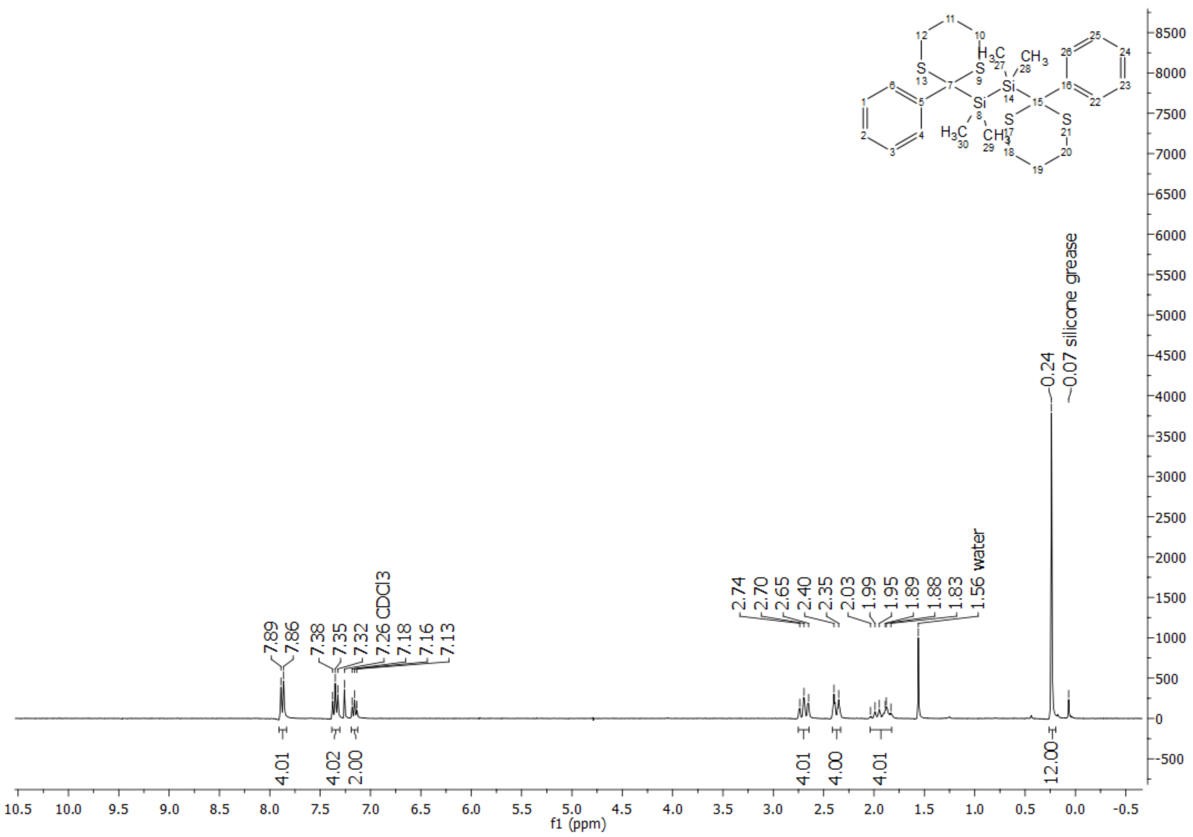

Figure S19: ^{1}H−NMR spectra ($CDCl_3$) of 1,1,2,2−tetramethyl−1,2−bis(2−phenyl−1,3−dithian−2−yl)disilane (**5a**)

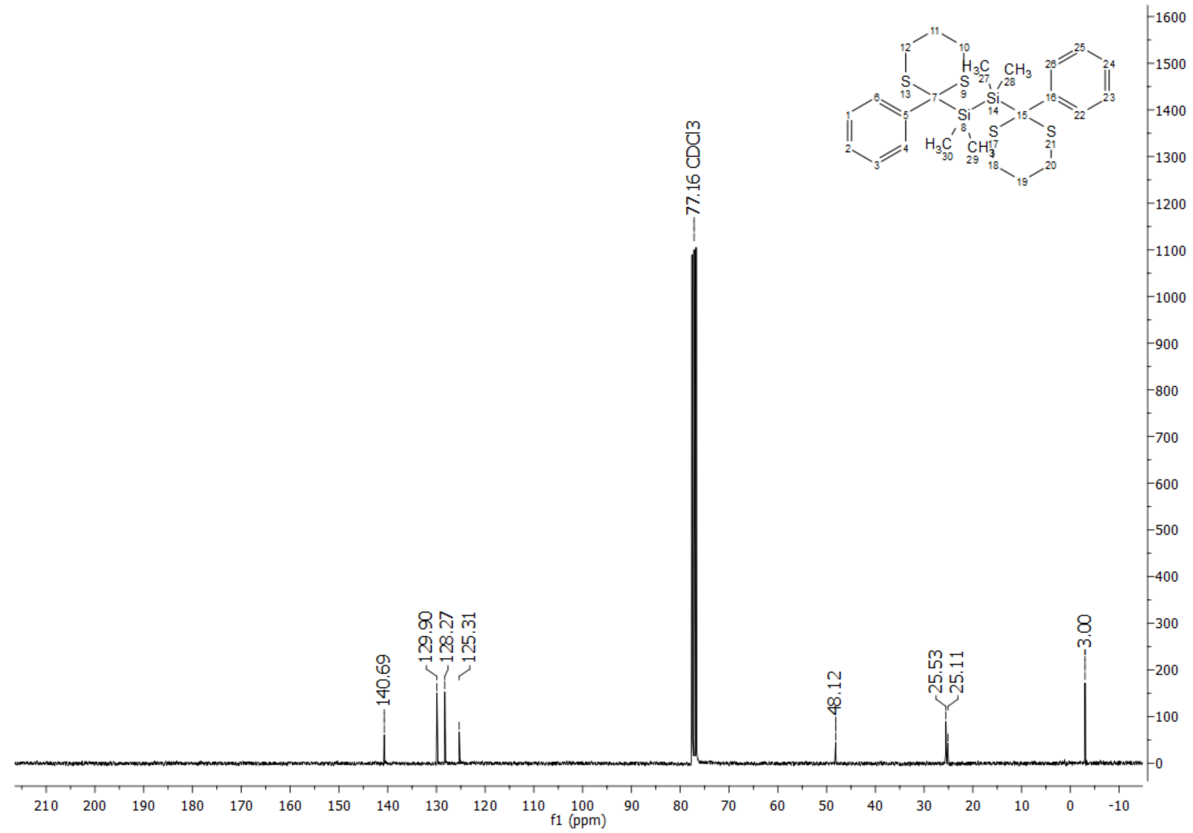

Figure S20: ^{13}C−NMR spectra ($CDCl_3$) of 1,1,2,2−tetramethyl−1,2−bis(2−phenyl−1,3−dithian−2−yl)disilane (**5a**)

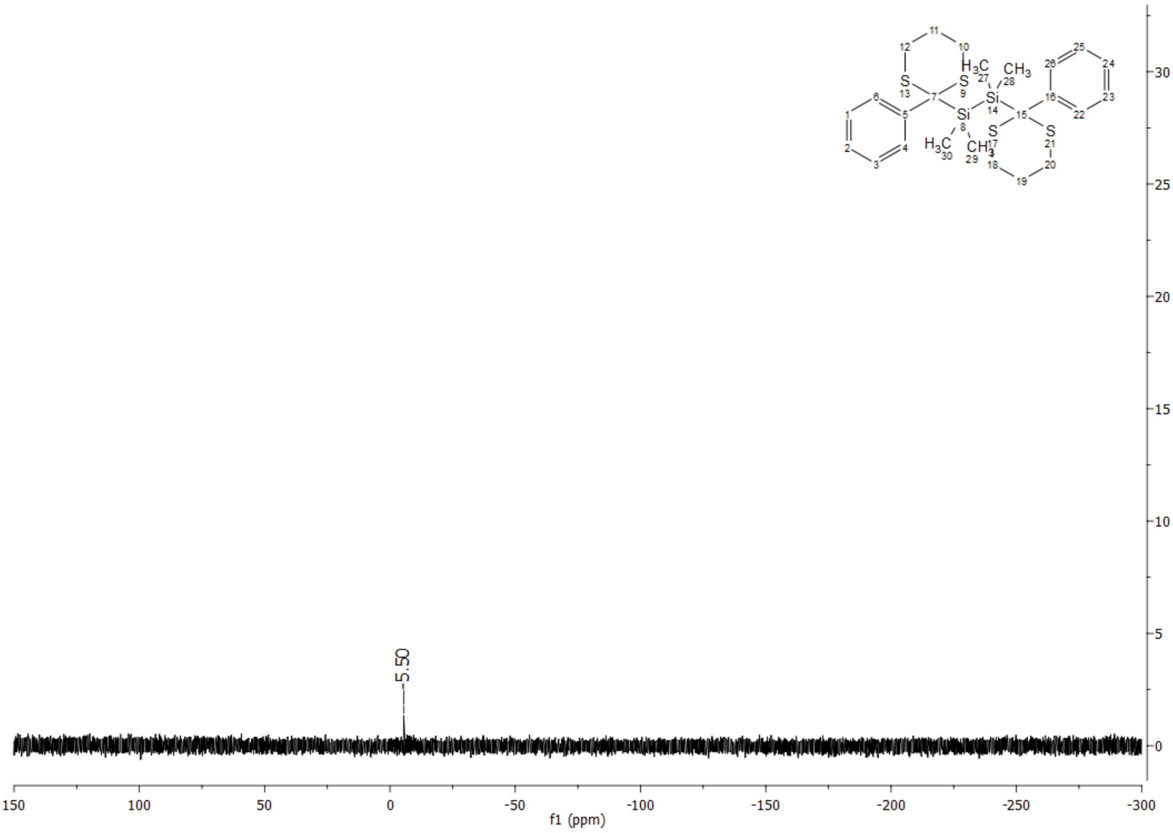

Figure S21: ^{29}Si−NMR spectra (C_6D_6) of 1,1,2,2−tetramethyl−1,2−bis(2−phenyl−1,3−dithian−2−yl)disilane (**5a**)

1.8. 1,1,2,2−tetraethyl−1,2−bis(2−phenyl−1,3−dithian−2−yl)disilane (5b)

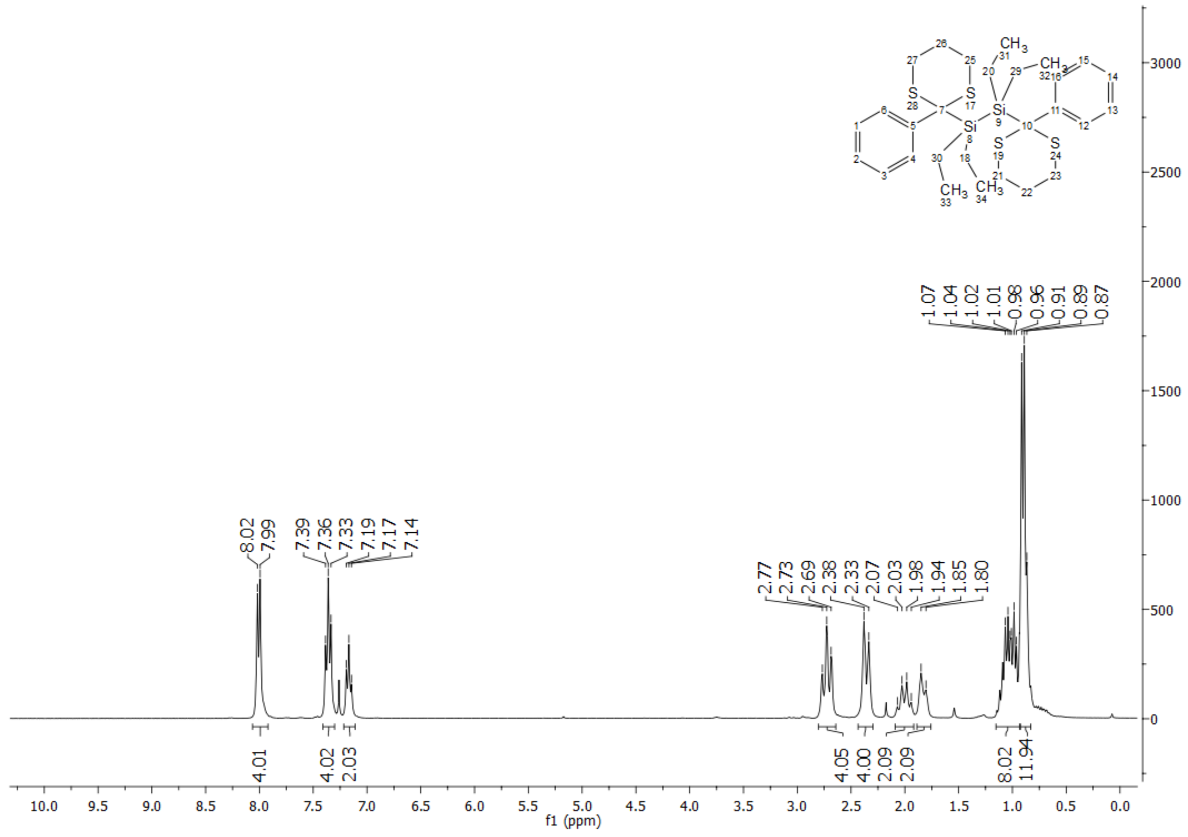

Figure S22: ^{1}H−NMR spectra ($CDCl_3$) of tetraethyl−bis−(2−phenyl−1,3−dithian−2−yl)disilane (**5b**)

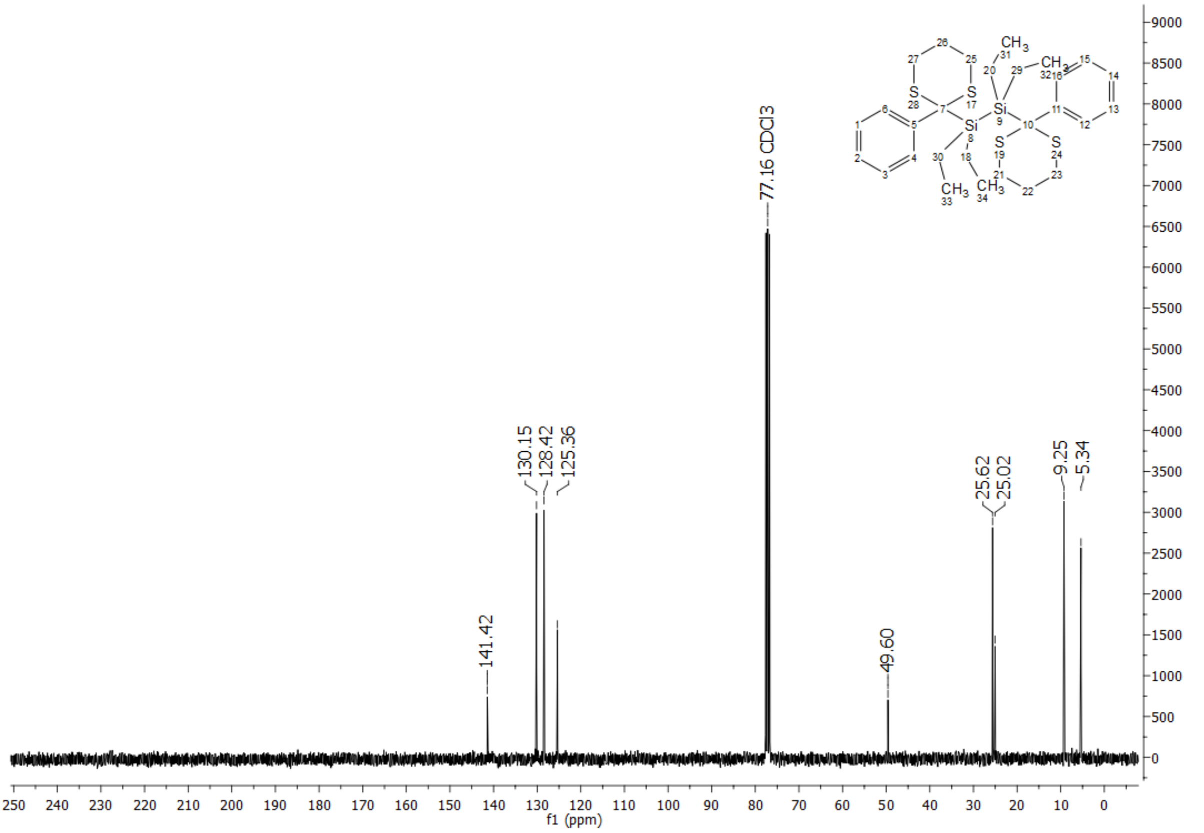

Figure S23: ^{13}C−NMR spectra ($CDCl_3$) of tetraethyl−bis−(2−phenyl−1,3−dithian−2−yl)disilane (**5b**)

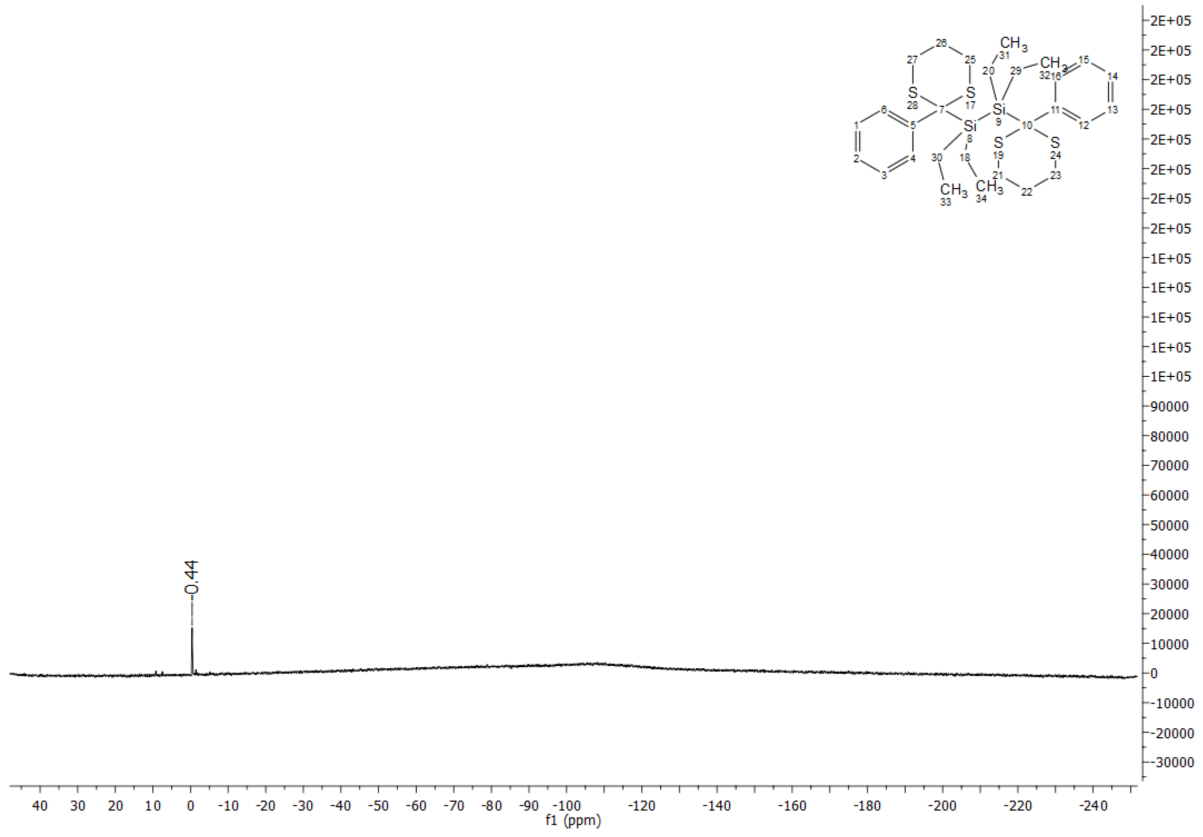

Figure S24: ^{29}Si−NMR spectra ($CDCl_3$) of tetraethyl−bis−(2−phenyl−1,3−dithian−2−yl)disilane (**5b**)

1.9. 2,2,3,3–tetramethyl–1,1,4,4–tetraphenyl–1,4–bis(2–phenyl–1,3–dithian–2–yl)tetrasilane (5c)

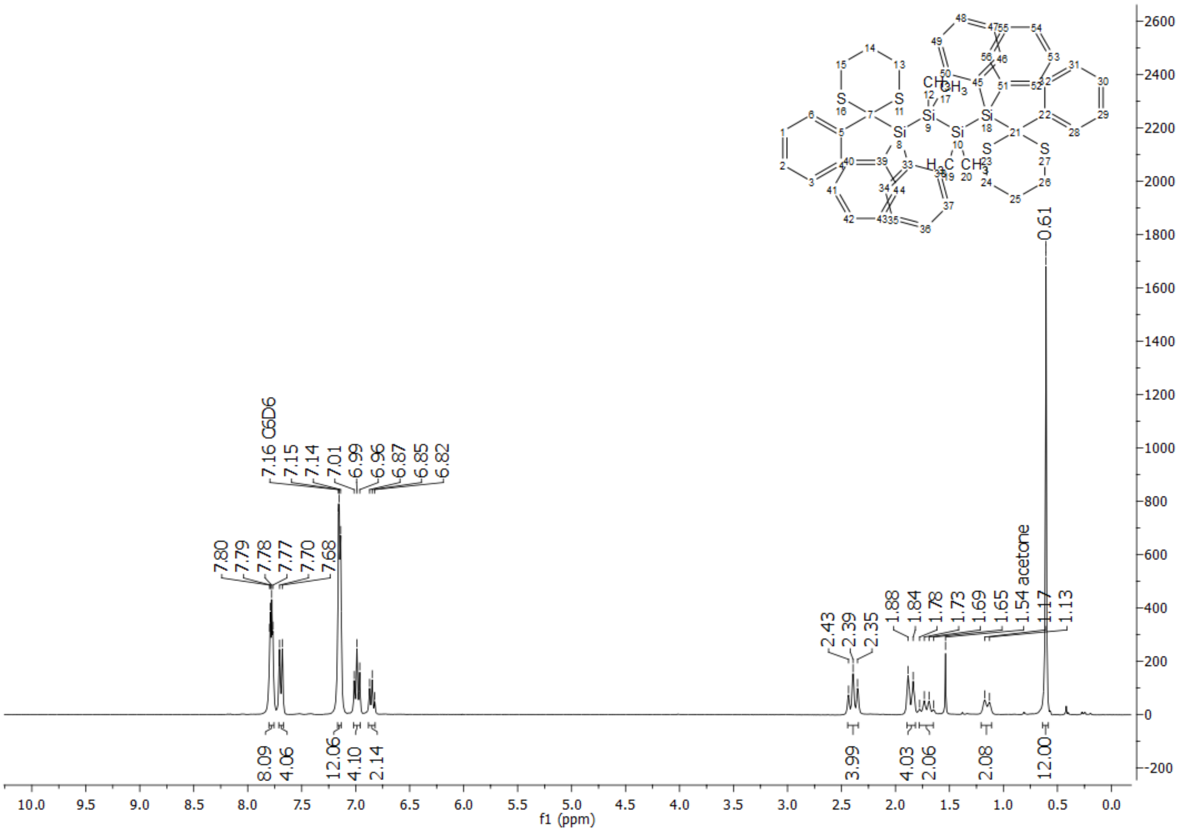

Figure S25: ^{1}H–NMR spectra (C_6D_6) of 2,2,3,3–tetramethyl–1,1,4,4–tetraphenyl–1,4–bis(2–phenyl–1,3–dithian–2–yl)tetrasilane (**5c**)

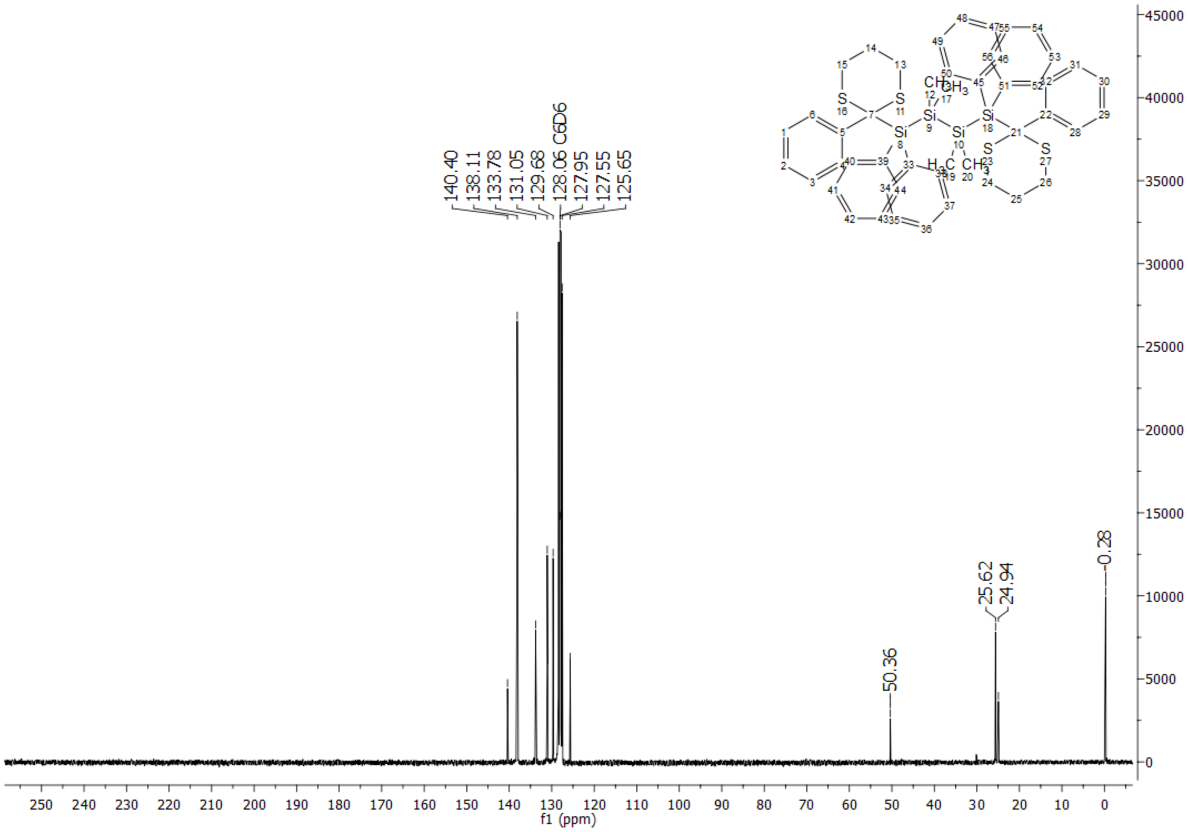

Figure S26: ^{13}C–NMR spectra (C_6D_6) of 2,2,3,3–tetramethyl–1,1,4,4–tetraphenyl–1,4–bis(2–phenyl–1,3–dithian–2–yl)tetrasilane (**5c**)

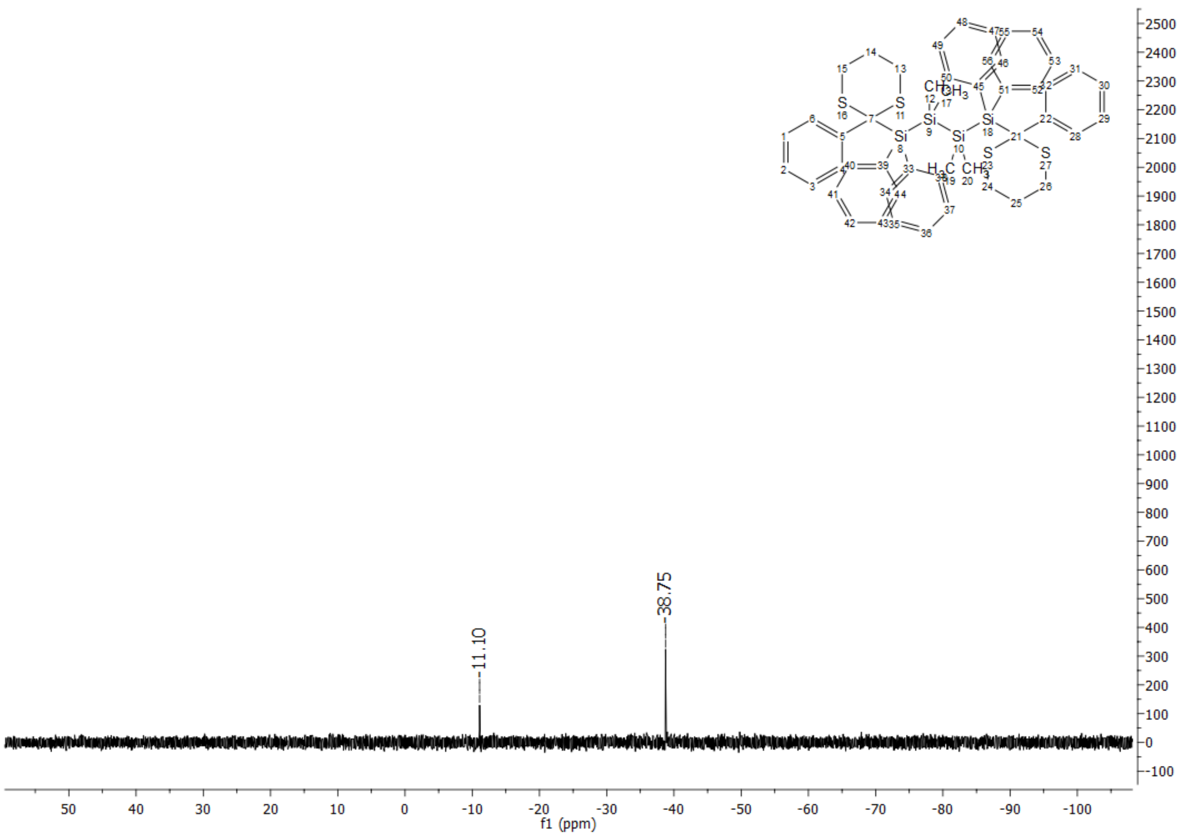

Figure S27: ^{29}Si−NMR spectra (C_6D_6) of 2,2,3,3−tetramethyl−1,1,4,4−tetraphenyl−1,4−bis(2−phenyl−1,3−dithian−2−yl)tetrasilane (**5c**)

1.10. (1,1,2,2−tetraethyldisilane−1,2−diyl)bis(phenylmethanone) (6a)

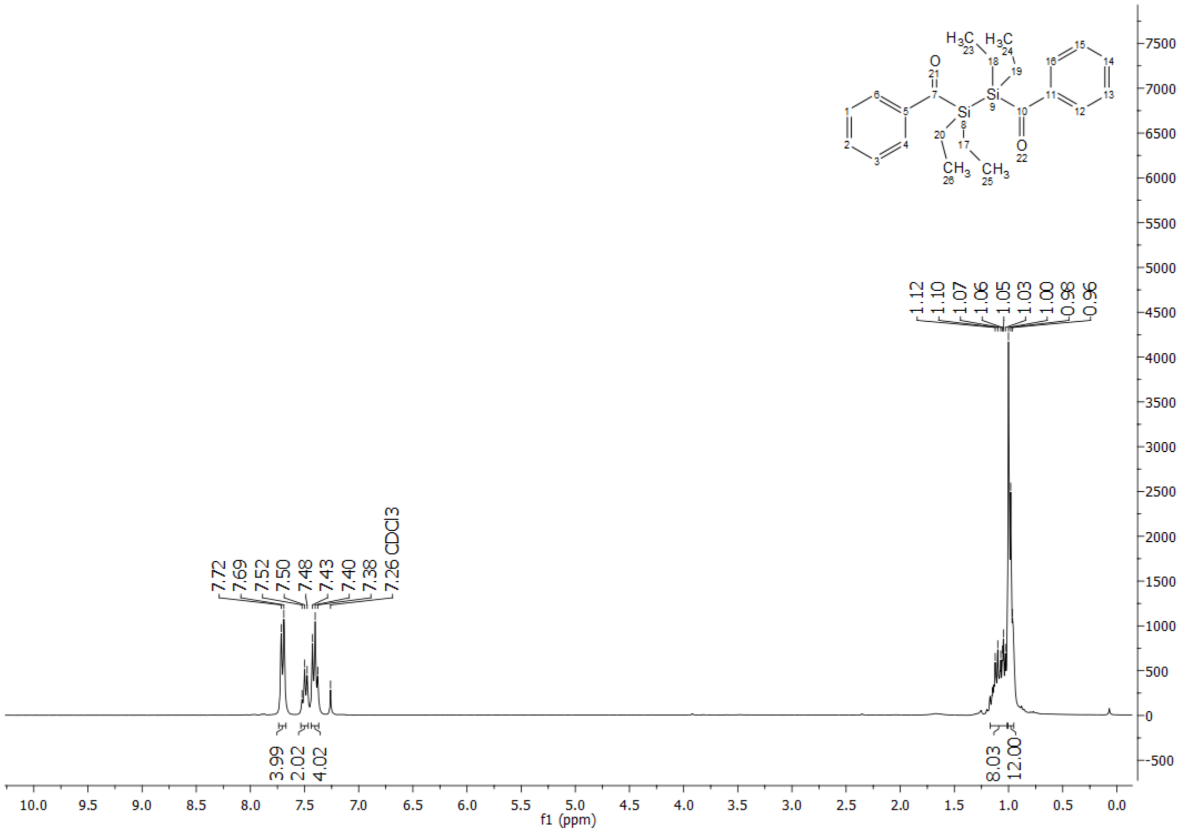

Figure S28: ^{1}H−NMR spectra ($CDCl_3$) of (1,1,2,2−tetraethyldisilane−1,2−diyl)bis(phenylmethanone) (**6a**)

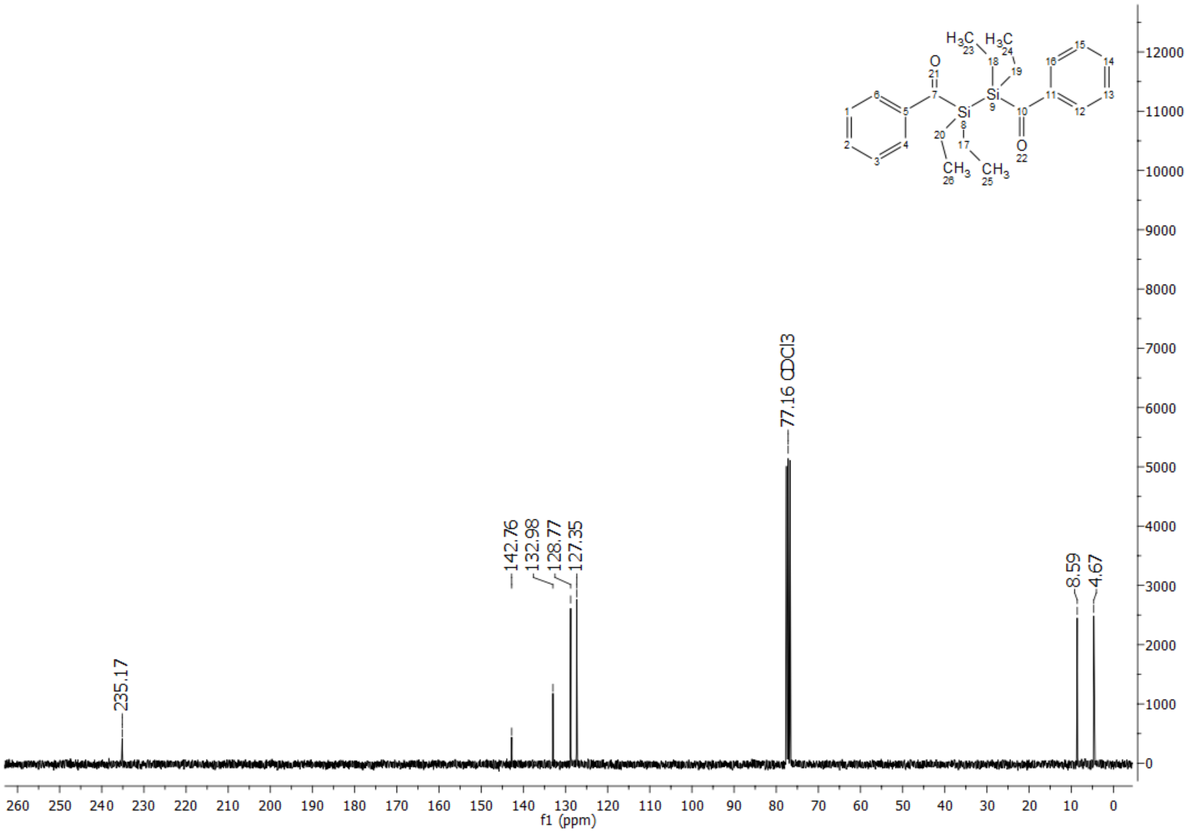

Figure S29: ^{13}C–NMR spectra ($CDCl_3$) of (1,1,2,2–tetraethyldisilane–1,2–diyl)bis(phenylmethanone) (**6a**)

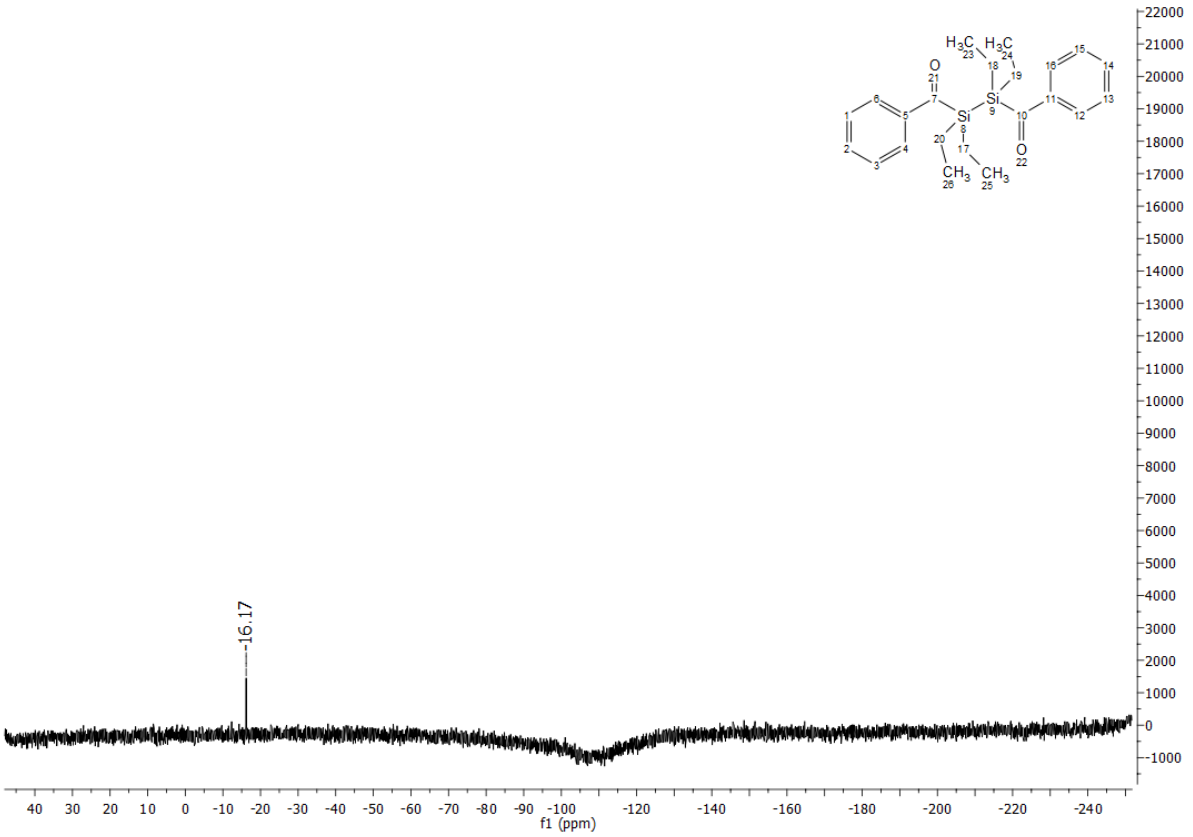

Figure S30: ^{29}Si–NMR spectra (C_6D_6) of (1,1,2,2–tetraethyldisilane–1,2–diyl)bis(phenylmethanone) (**6a**)

1.11. (2,2,3,3−tetramethyl−1,1,4,4−tetraphenyltetrasilane−1,4−diyl)bis(phenylmethanone) (6b)

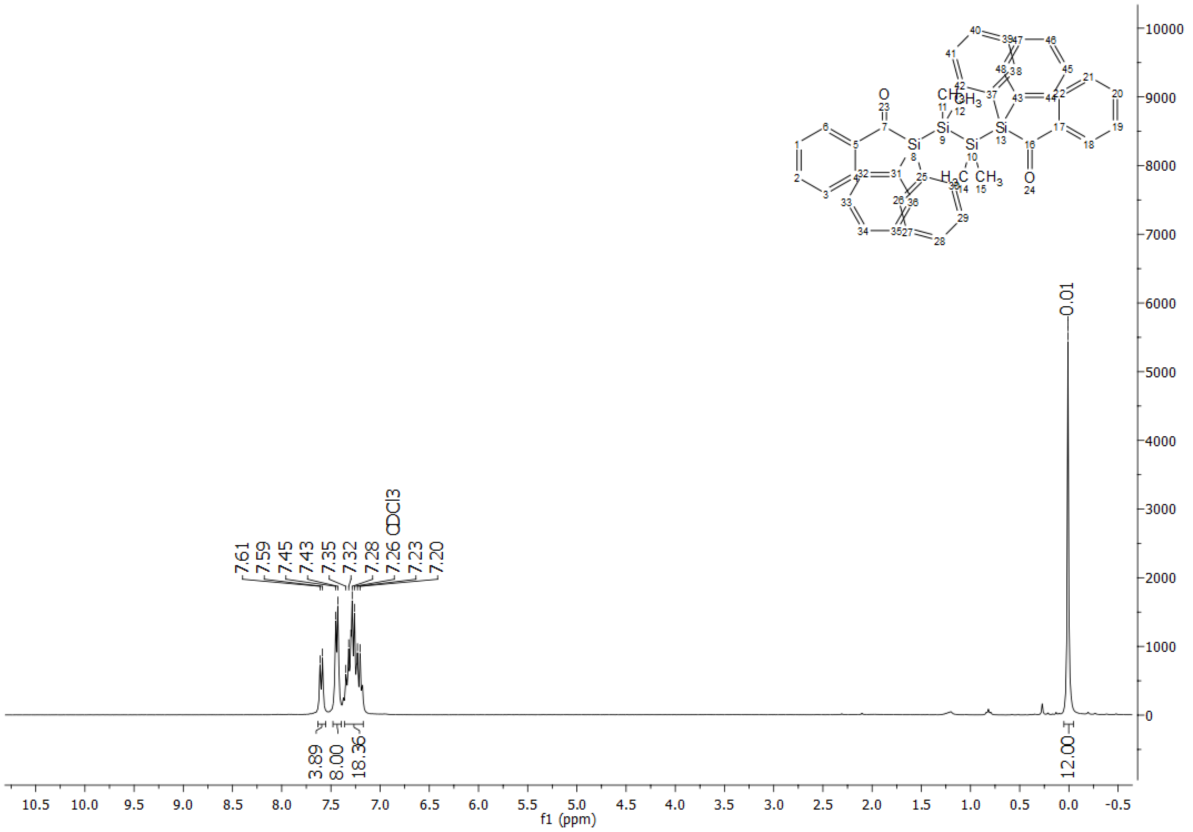

Figure S31: ^{1}H−NMR spectra ($CDCl_3$) of (2,2,3,3−tetramethyl−1,1,4,4−tetraphenyltetrasilane−1,4−diyl)bis(phenylmethanone) (**6b**)

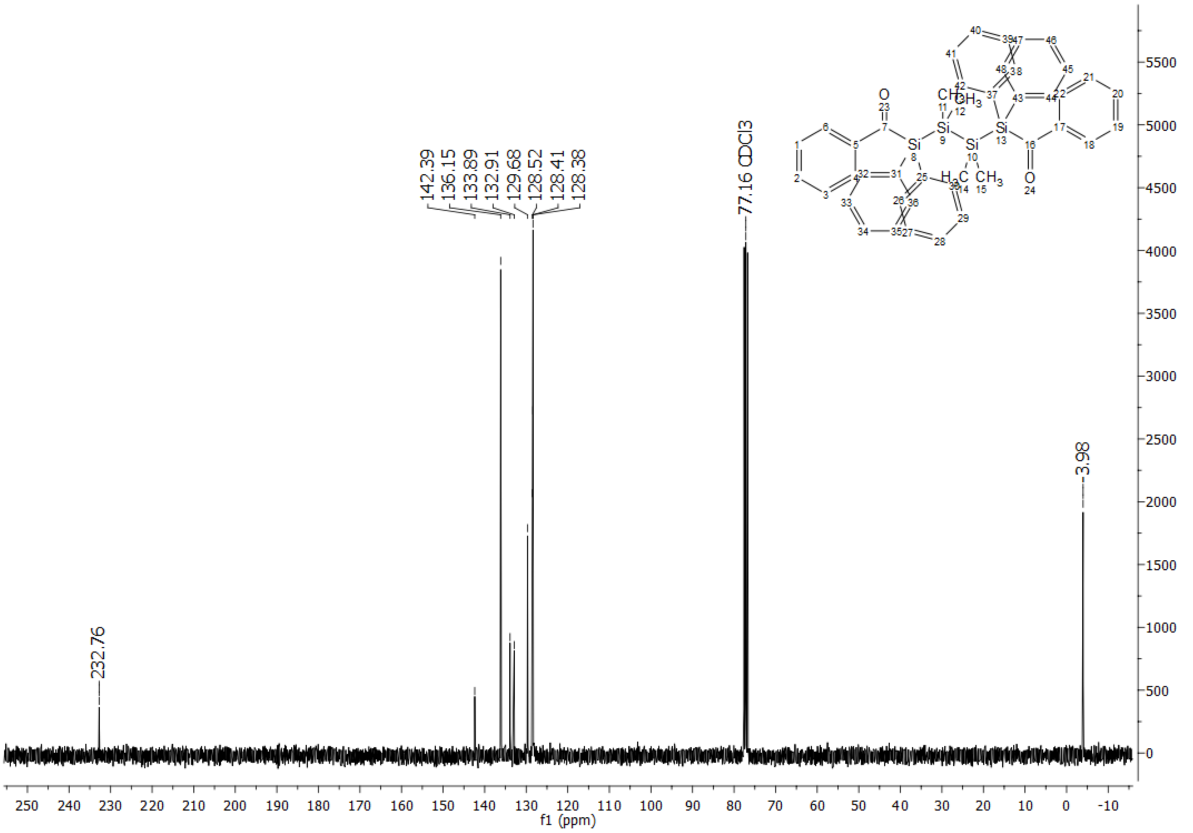

Figure S32: ^{13}C−NMR spectra ($CDCl_3$) of (2,2,3,3−tetramethyl−1,1,4,4−tetraphenyltetrasilane−1,4−diyl)bis(phenylmethanone) (**6b**)

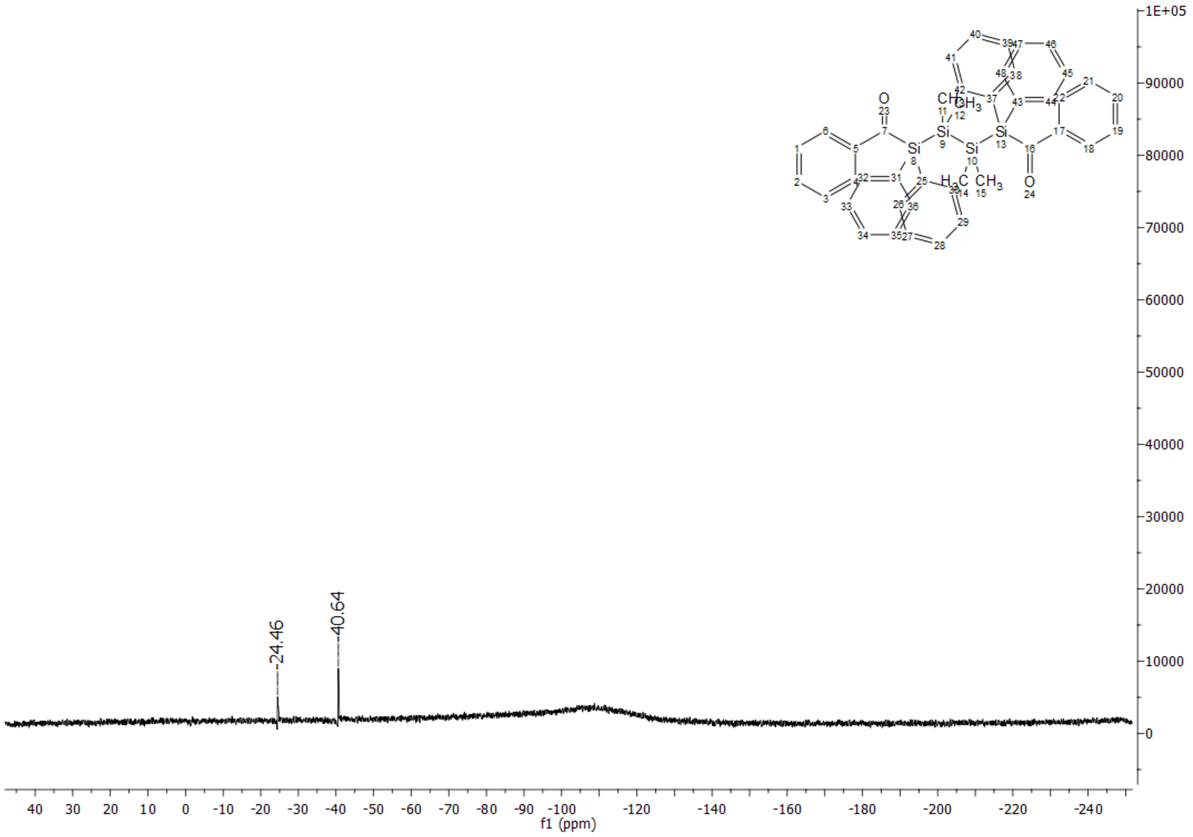

Figure S33: ^{29}Si−NMR spectra ($CDCl_3$) of (2,2,3,3−tetramethyl−1,1,4,4−tetraphenyltetrasilane−1,4−diyl)bis(phenylmethanone) (**6b**)

1.12. (((fluorodimethylsilyl)(methoxy)(phenyl)methyl)dimethylsilyl)(phenyl)methanone (6c)

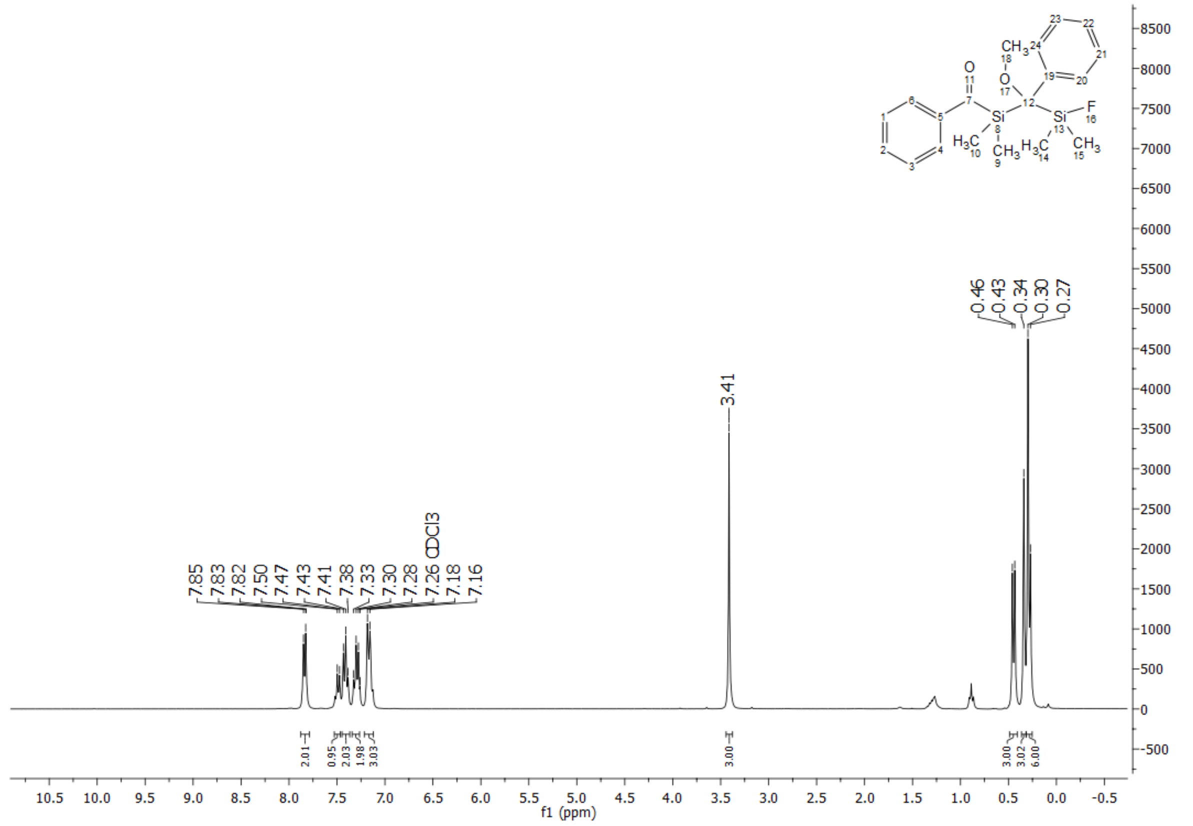

Figure S34: ^{1}H−NMR spectra ($CDCl_3$) of (((fluorodimethylsilyl)(methoxy)(phenyl)methyl)dimethylsilyl)(phenyl)methanone (**6c**)

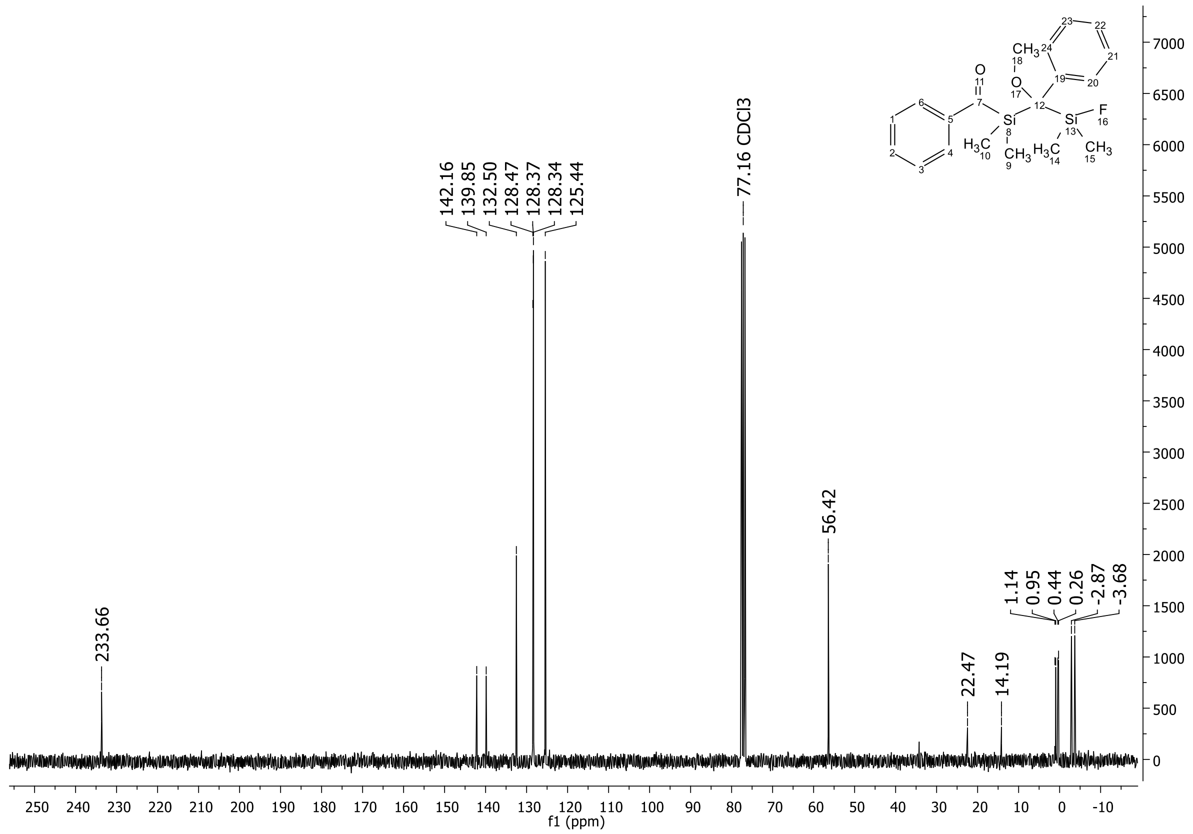

Figure S35: ^{13}C–NMR spectra ($CDCl_3$) of (((fluorodimethylsilyl)(methoxy)(phenyl)methyl)dimethylsilyl)(phenyl)methanone (**6c**)

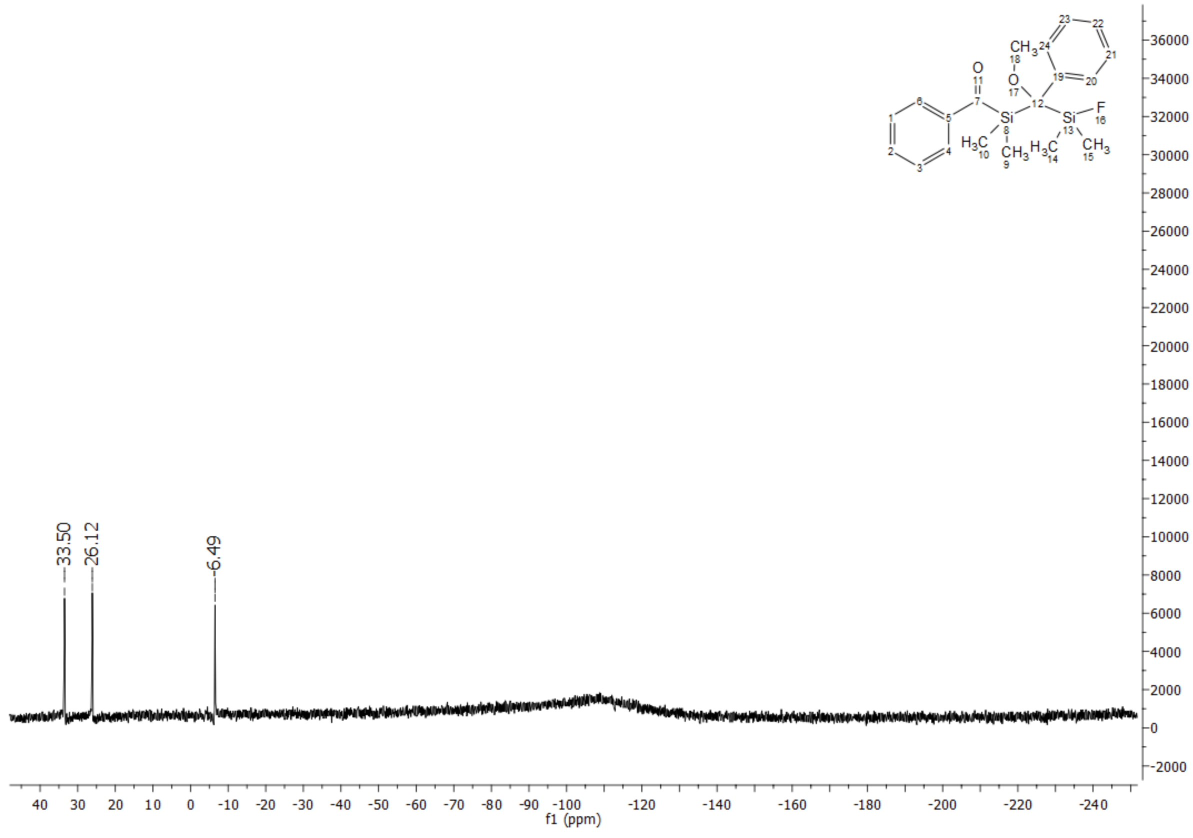

Figure S36: ^{29}Si–NMR spectra ($CDCl_3$) of (((fluorodimethylsilyl)(methoxy)(phenyl)methyl)dimethylsilyl)(phenyl)methanone (**6c**)

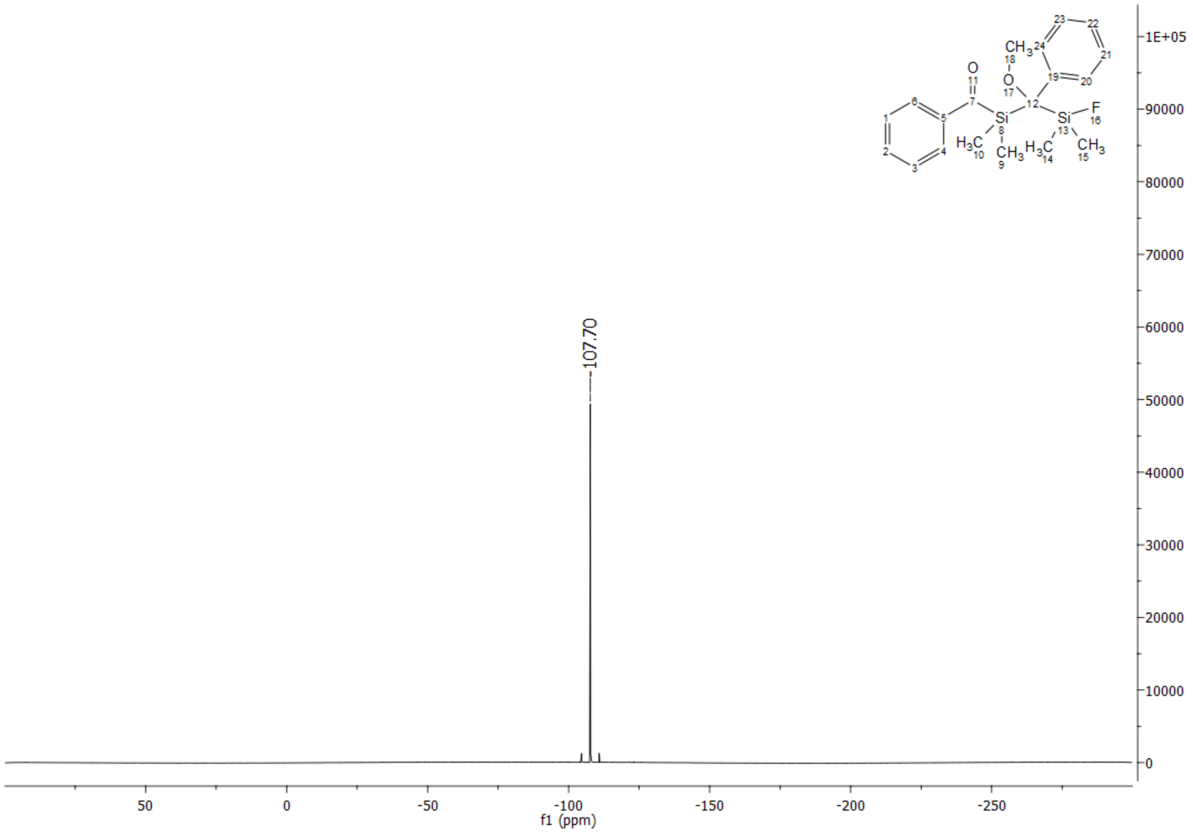

Figure S37: ^{19}F−NMR spectra (C_6D_6) of (((fluorodimethylsilyl)(methoxy)(phenyl)methyl)dimethylsilyl)(phenyl)methanone (**6c**)

1.13. phenyl(1,1,2,2−tetramethyl−2−(2−phenyl−1,3−dithian−2−yl)disilaneyl)methanone (6d)

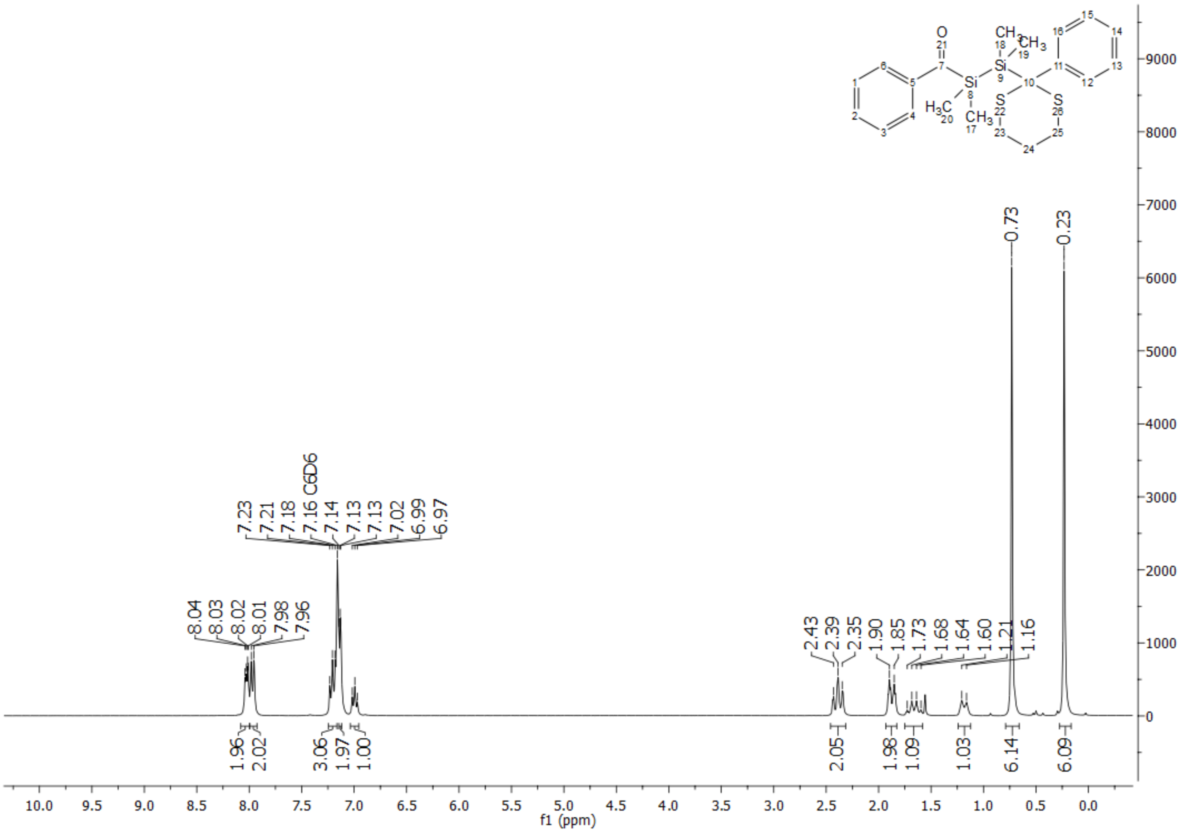

Figure S38: ^{1}H−NMR spectra (C_6D_6) of phenyl(1,1,2,2−tetramethyl−2−(2−phenyl−1,3−dithian−2−yl)disilaneyl)methanone (**6d**)

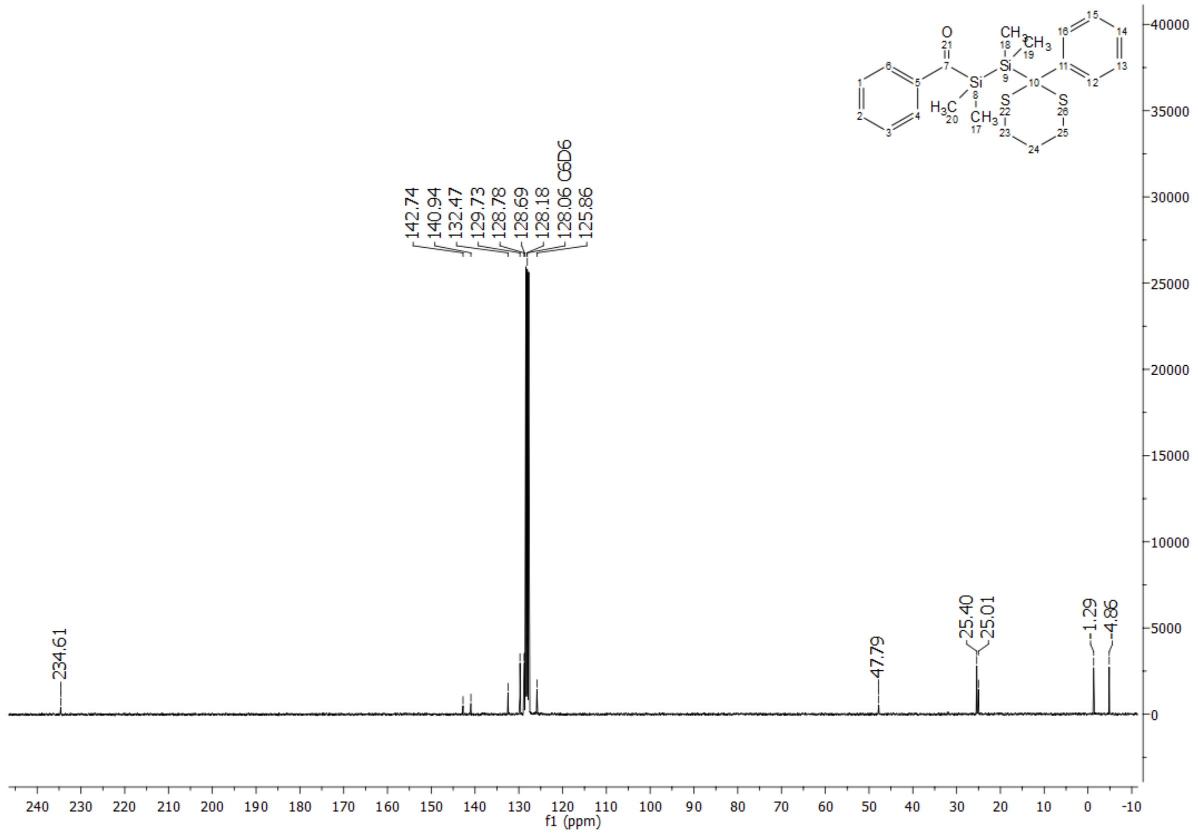

Figure S39: ^{13}C−NMR spectra (C_6D_6) of phenyl(1,1,2,2−tetramethyl−2−(2−phenyl−1,3−dithian−2−yl)disilaneyl)methanone (**6d**)

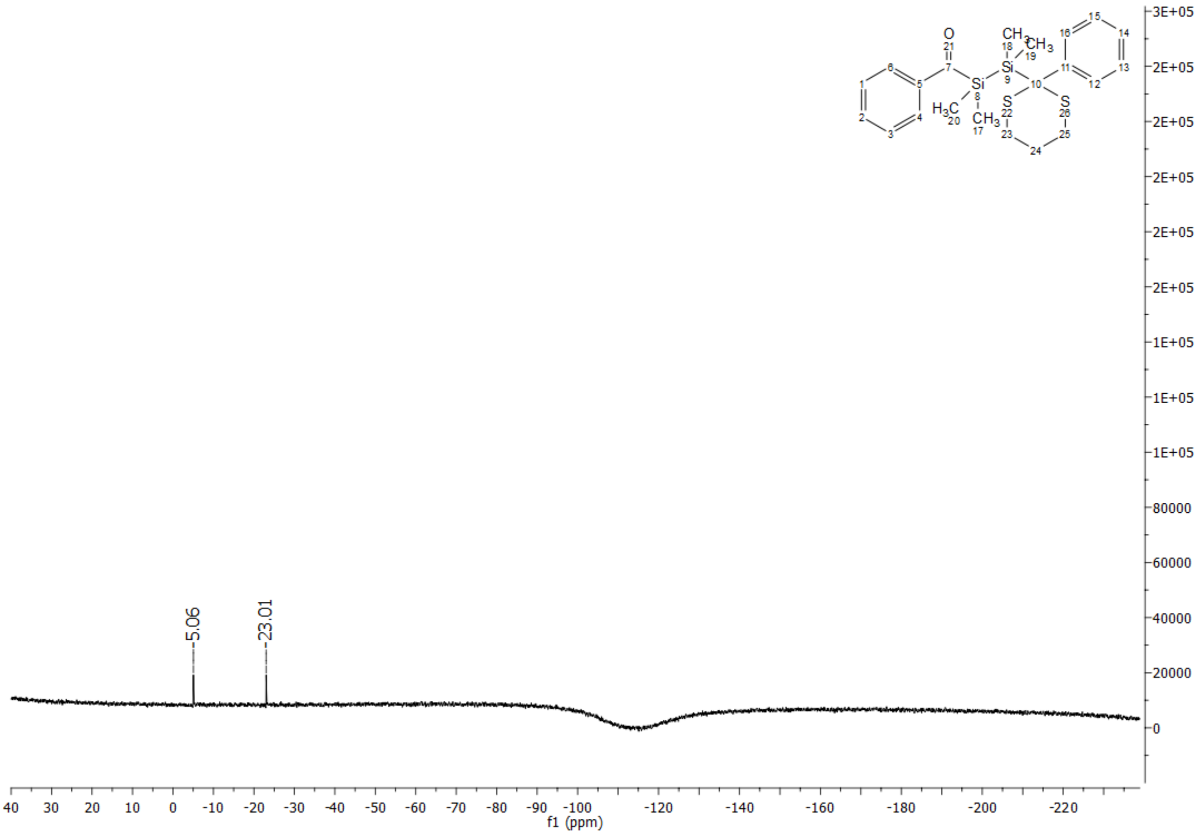

Figure S40: ^{29}Si−NMR spectra (C_6D_6) of phenyl(1,1,2,2−tetramethyl−2−(2−phenyl−1,3−dithian−2−yl)disilaneyl)methanone (**6d**)

2. X–ray Crystallography

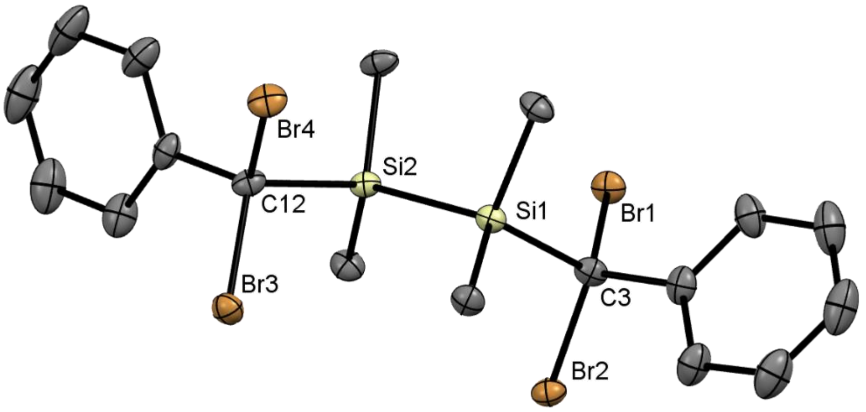

Figure S41: ORTEP representation for compound **3a**. Thermal ellipsoids are depicted at the 50% probability level. Hydrogen atoms are omitted for clarity. Selected bond lengths (Å) and bond angles (deg) with estimated standard deviations: Si(1)–Si(2) 2.374(14), Si(1)–C(3) 1.928(4), Si(2)–C(12) 1.939(4), C(3)–Br(1) 1.989(4), C(3)–Br(2) 1.980(4), C(3)–Si(1)–Si(2) 108.04(12), C(12)–Si(2)–Si(1) 109.72(12), Si(1)–C(3)–Br(1) 106.27(18), Si(1)–C(3)–Br(2) 108.85(18), Br(1)–C(3)–Br(2) 105.34(17).

Table S1: Crystallographic data of compounds **3a**, **3d**, **4c**, **4d** and **6c.**

Compound	3a	3d	4c	4d	6c
Formula	$C_{18}H_{22}Br_4Si_2$	$C_{31}H_{25}Br_{1.94}Cl_{1.06}Si_2$	$C_{38}H_{30}O_2Si_2 \cdot C_6H_6$	$C_{38}H_{31}BrOSi_2$	$C_{19}H_{25}FO_2Si_2$
M_r (g mol^{-1})	614.17	646.03	652.91	639.72	360.57
a (Å)	6.6811 (3)	10.2892 (4)	8.7215 (3)	19.4237 (12)	13.1131 (11)
b (Å)	13.5263 (5)	11.6856 (5)	9.6682 (3)	9.8684 (5)	7.6171 (8)
c (Å)	12.4593 (5)	23.2939 (10)	12.1097 (4)	32.7620 (2)	19.1920 (15)
α (°)	90	90	104.211 (1)	90	90
β (°)	104.510 (1)	98.676 (2)	103.492 (1)	91.723 (4)	97.682 (3)
γ (°)	90	90	111.486 (1)	90	90
V (Å3)	1090.04 (8)	2768.7 (2)	859.90 (5)	6277.0 (7)	1899.8 (3)
Z	2	4	1	8	2
Crystal size (mm)	0.35 × 0.29 × 0.19	0.30 × 0.26 × 0.21	0.20 × 0.16 × 0.15	0.17 × 0.13 × 0.08	0.24 × 0.21 × 0.16
Crystal habit	Block, colourless	Block, colourless	Block, yellow	Block, yellow	Block, yellow
Crystal system	Monoclinic	Monoclinic	Triclinic	Monoclinic	Monoclinic
Space group	*P*21	*P*21/*n*	*P*–1	P21/c	P21/c
d_{calc} (Mg m^{-3})	1.871	1.550	1.261	1.354	1.261
μ (mm^{-1})	7.49	3.04	0.14	1.42	0.20
T (K)	100	100	100	100	100
2θ range (°)	2.3–32.6	2.3–32.9	2.4–33.2	2.1–54.6	2.5–29.9
F (000)	596	1299	344	264	768
T_{min}, T_{max}	0.391, 0.747	0.472, 0.747	0.652, 0.747	0.802, 0.893	0.676, 0.746
R_{int}	0.093	0.085	0.042	0.088	0.056
No. of measured, and independent [I > 2σ(I)] reflections	88203, 8287	129447, 4872	47500, 3019	51302, 51302	90733, 5548
independent reflections	8287	4872	3019	51302	5548
No. of parameters, restraints	222, 1	345, 0	217, 0	758, 78	222, 0

Δ›max, Δ›min (e Å^{-3})	1.06, −0.70	0.52, −0.33	0.42, −0.33	1.16, −0.97	0.52, −0.46
R1, wR2 (all data)	R1 = 0.035<br>wR2 = 0.074	R1 = 0.026<br>wR2 = 0.060	R1 = 0.035<br>wR2 = 0.082	R1 = 0.121<br>wR2 = 0.262	R1 = 0.046<br>wR2 = 0.098
R1, wR2 (>2σ)	R1 = 0.030<br>wR2 = 0.073	R1 = 0.024<br>wR2 = 0.061	R1 = 0.033<br>wR2 = 0.080	R1 = 0.090<br>wR2 = 0.241	R1 = 0.036<br>wR2 = 0.091

3. Photolysis Experiments

Photolysis was performed by dissolving approximately 100 mg of each compound (**4b**,**c**) in benzene. An excess of methanol and a view drops of triethylamine as auxiliary base were added. Further, it was irradiated at a wavelength of 405 nm until photolysis was finished, approximately after 30 minutes. In the case of **4b**, the product mixture was separated from formed polymers by suspending it in *n*−pentane and transferring it via syringe filter into another flask. Removing of the solvent and analysis via NMR−spectroscopy gave rise to the formed photoproducts (as shown in S45−S50). However, isolation of any of these products despite several attempts of separation and purification was not possible for us.

3.1. NMR−Data of the Photolysis of Compound 4b

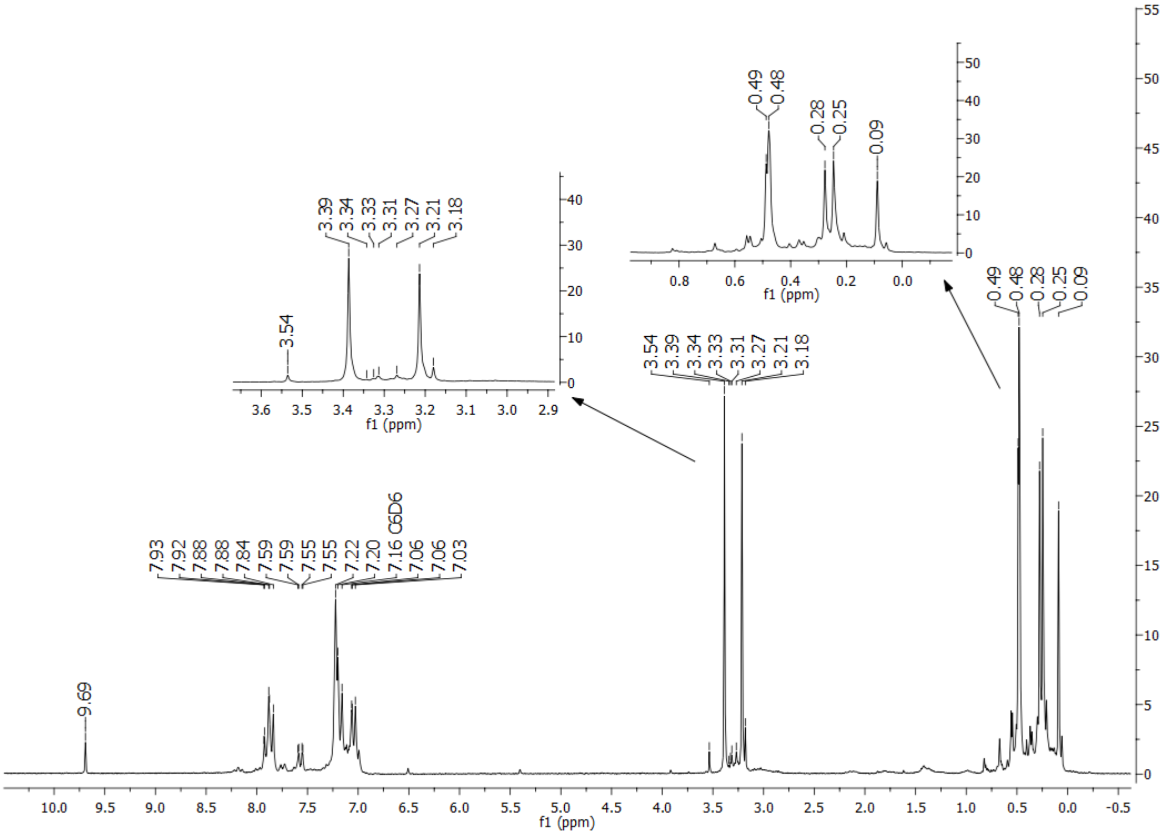

Figure S42: ^{1}H−NMR spectra (C_6D_6) after photolysis of compound **4b**

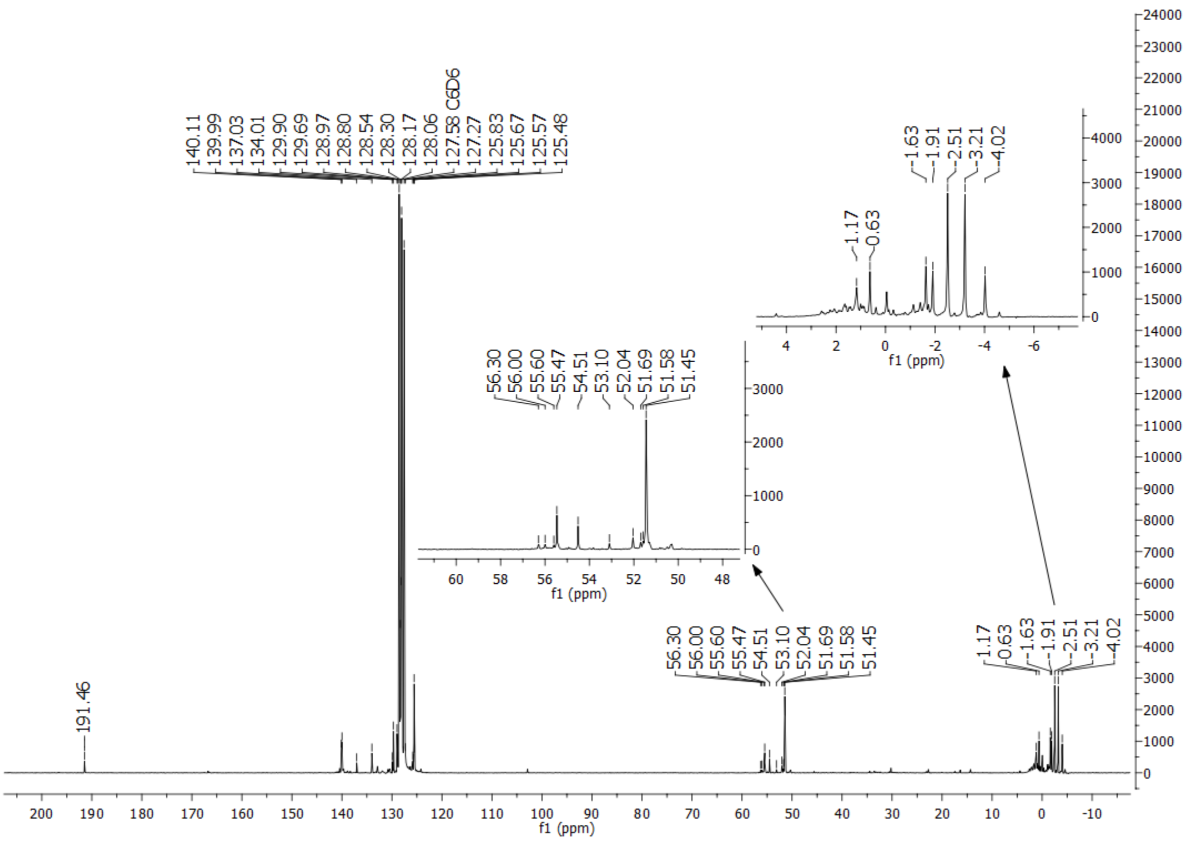

Figure S43: ^{13}C−NMR spectra (C_6D_6) after photolysis of compound **4b**

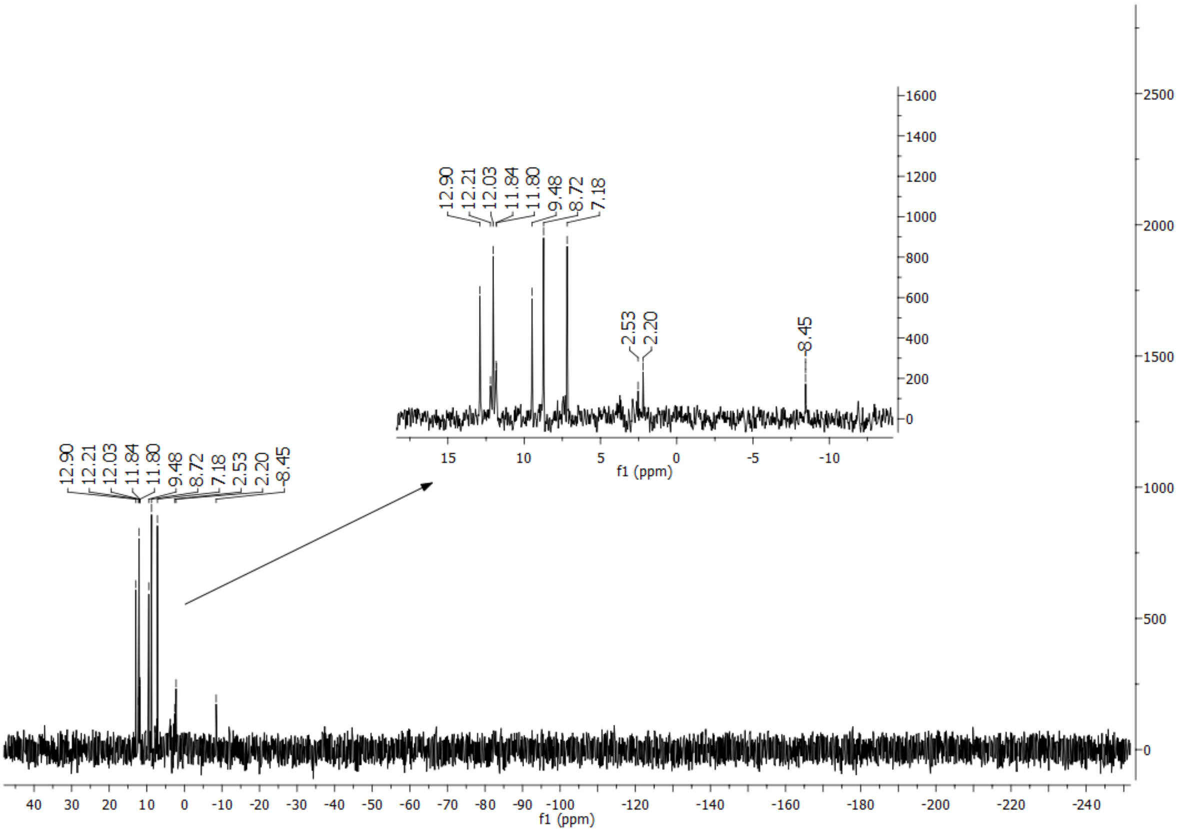

Figure S44: ^{29}Si−NMR spectra (C_6D_6) after photolysis of compound **4b**

3.1.1. NMR–Data of the Photolysis of Compound 4c

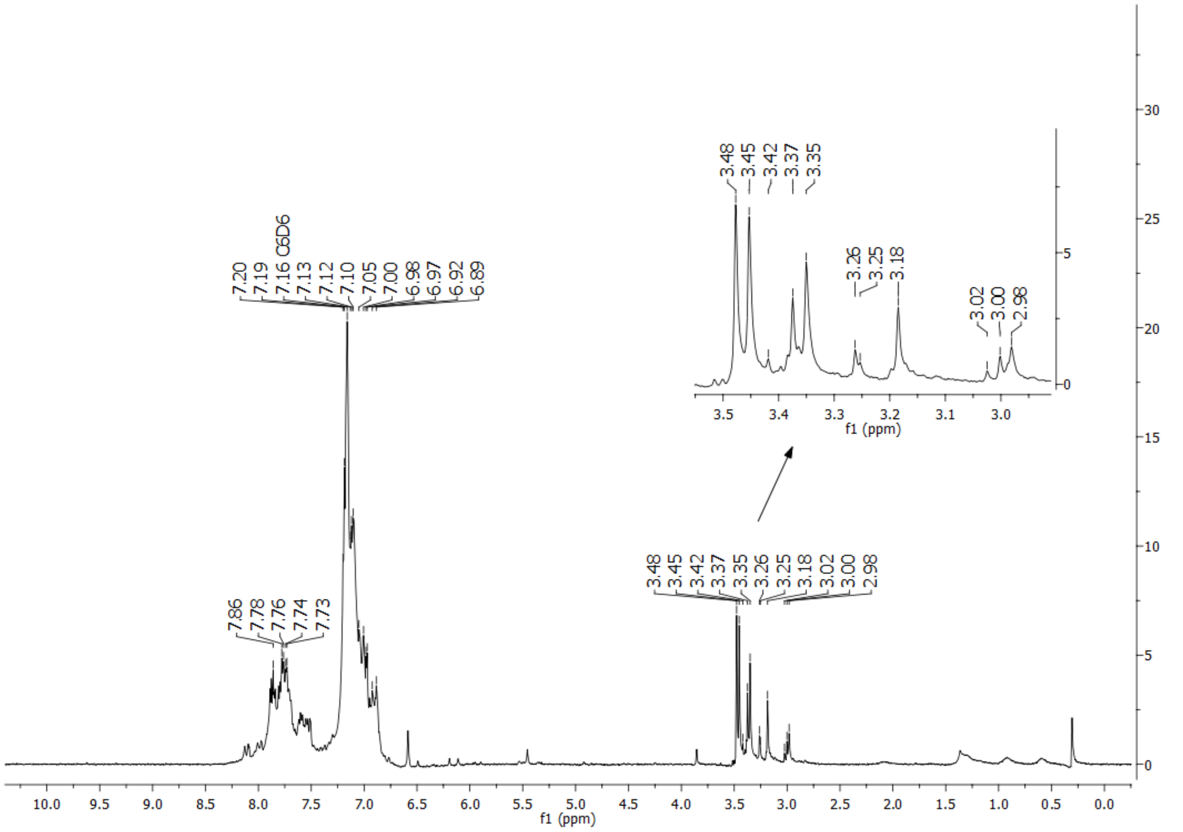

Figure S45: ^{1}H–NMR spectra (C_6D_6) after photolysis of compound **4c**

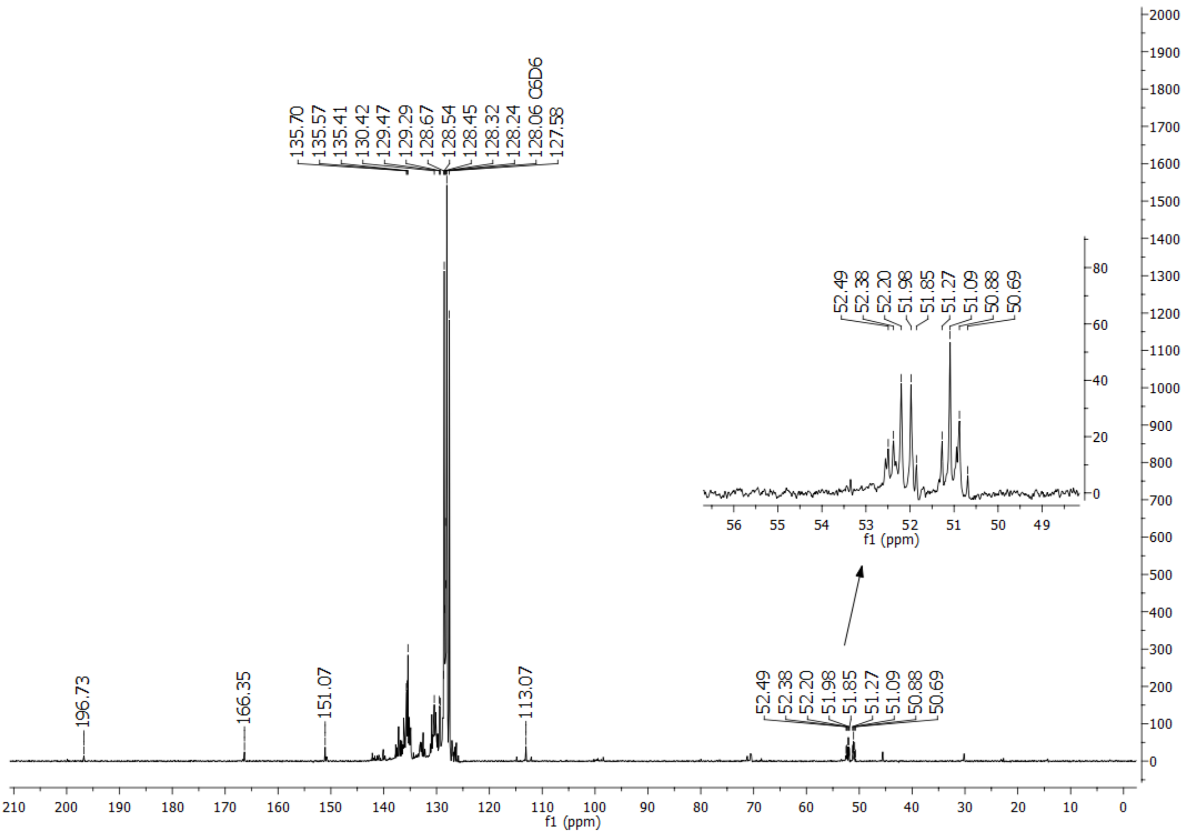

Figure S46: ^{13}C–NMR spectra (C_6D_6) after photolysis of compound **4c**

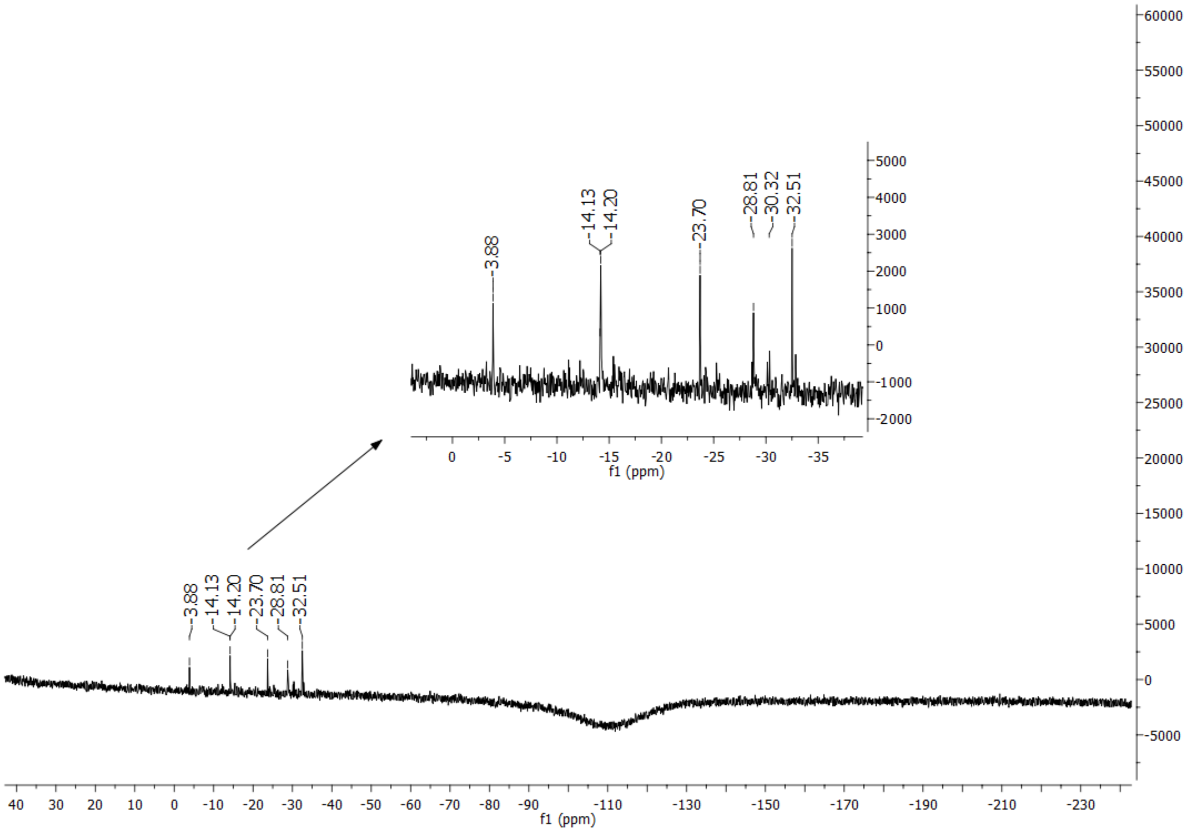

Figure S47: ^{29}Si−NMR spectra (C_6D_6) after photolysis of compound **4c**
